# long-read-tools.org: an interactive catalogue of analysis methods for long-read sequencing data

**DOI:** 10.1093/gigascience/giab003

**Published:** 2021-02-16

**Authors:** Shanika L Amarasinghe, Matthew E Ritchie, Quentin Gouil

**Affiliations:** Epigenetics and Development Division, The Walter and Eliza Hall Institute of Medical Research, 1G Royal Parade, Parkville, VIC 3052, Australia; Department of Medical Biology, The University of Melbourne, 1G Royal Parade, Parkville, VIC 3052, Australia; Epigenetics and Development Division, The Walter and Eliza Hall Institute of Medical Research, 1G Royal Parade, Parkville, VIC 3052, Australia; Department of Medical Biology, The University of Melbourne, 1G Royal Parade, Parkville, VIC 3052, Australia; School of Mathematics and Statistics, The University of Melbourne, 813 Swanston Street, Parkville, VIC 3010, Australia; Epigenetics and Development Division, The Walter and Eliza Hall Institute of Medical Research, 1G Royal Parade, Parkville, VIC 3052, Australia; Department of Medical Biology, The University of Melbourne, 1G Royal Parade, Parkville, VIC 3052, Australia

**Keywords:** database, long-read sequencing, data analysis, nanopore, PacBio

## Abstract

**Background:**

The data produced by long-read third-generation sequencers have unique characteristics compared to short-read sequencing data, often requiring tailored analysis tools for tasks ranging from quality control to downstream processing. The rapid growth in software that addresses these challenges for different genomics applications is difficult to keep track of, which makes it hard for users to choose the most appropriate tool for their analysis goal and for developers to identify areas of need and existing solutions to benchmark against.

**Findings:**

We describe the implementation of long-read-tools.org, an open-source database that organizes the rapidly expanding collection of long-read data analysis tools and allows its exploration through interactive browsing and filtering. The current database release contains 478 tools across 32 categories. Most tools are developed in Python, and the most frequent analysis tasks include base calling, *de novo* assembly, error correction, quality checking/filtering, and isoform detection, while long-read single-cell data analysis and transcriptomics are areas with the fewest tools available.

**Conclusion:**

Continued growth in the application of long-read sequencing in genomics research positions the long-read-tools.org database as an essential resource that allows researchers to keep abreast of both established and emerging software to help guide the selection of the most relevant tool for their analysis needs.

## Background

Long-read sequencing technologies facilitate versatile exploration of genomes owing to their ability to generate reads spanning several thousand base pairs [1]. Long reads can be *de novo* assembled or mapped to a reference to identify complicated structural variants and novel or complete transcripts that may otherwise be difficult to distinguish with short-read sequencing [2–4]. Improvements in throughput, error, and cost reduction, as well as increased interest in tool development for downstream data analyses [5], all contribute to the broadening adoption of long-read data across research fields.

To keep up with the rapid growth in software for long-read analysis, we collated and categorized existing long-read analysis tools at long-read-tools.org. This database enables easy navigation of the available software, allowing users to filter by specific tasks to identify methods that suit their analysis objectives.

## Findings

### Data collection, database design, and implementation

The long-read-tools.org database is specifically designed to catalogue analysis tools for long reads generated from genuine (Pacific Biosciences [PacBio] and Oxford Nanopore Technologies [ONT]) and synthetic (e.g., Hi-C, 10x, Bionano Genomics) long-read technologies. Up-to-date data are collected from various sources including publications, preprints, social media posts, mining public (GitHub, PyPI, Anaconda, CRAN, Bioconductor) and private repositories, and via the tool submissions form accessible from the Submit tab.

The data collected in the form of a csv file are processed within the R environment [6]. In this csv file, each tool is categorized with a TRUE or FALSE value on the basis of its functionality and technology(ies) in focus. Available details of the tools such as the description, publication status, tool licence, and programming language are retrieved and stored. Furthermore, the total number of citations for each tool is retrieved via rcrossref (v1.0.0) [7] and stored, while the number of citations from the past year is obtained through the citecorp R package (v0.3.0) [8] from the COCI database [9]. Both citation metrics may serve as an indication of a tool’s popularity. Information on arXiv preprints is retrieved through the arXiv package (v0.5.19) [10]. Multiple JSON files are generated during the processing step to populate the website. If publicly available, a tool’s source code is checked to assess the current status of its code base (e.g., actively maintained or deprecated).

Several analysis-style plots are created to be displayed on the database as well. The original csv input is processed to extract details such as the number of tools across time, the distribution of tools across categories, publication status, and the main programming platforms used in tool development to summarize the contents of the database. The plots are created in the R environment using several main packages such as ggplot2 (v3.3.2) [11] and plotly (v4.9.2) [12].

### Database usage

The long-read-tools.org website consists of several tabs, the first of which is the landing page (Home), which provides a summary of the database. The second Table tab contains the primary table with information that can be filtered using the search bar on the right. This tab can be used to view and download the required details of the complete database or a set of tools of interest.

**Figure 1:**
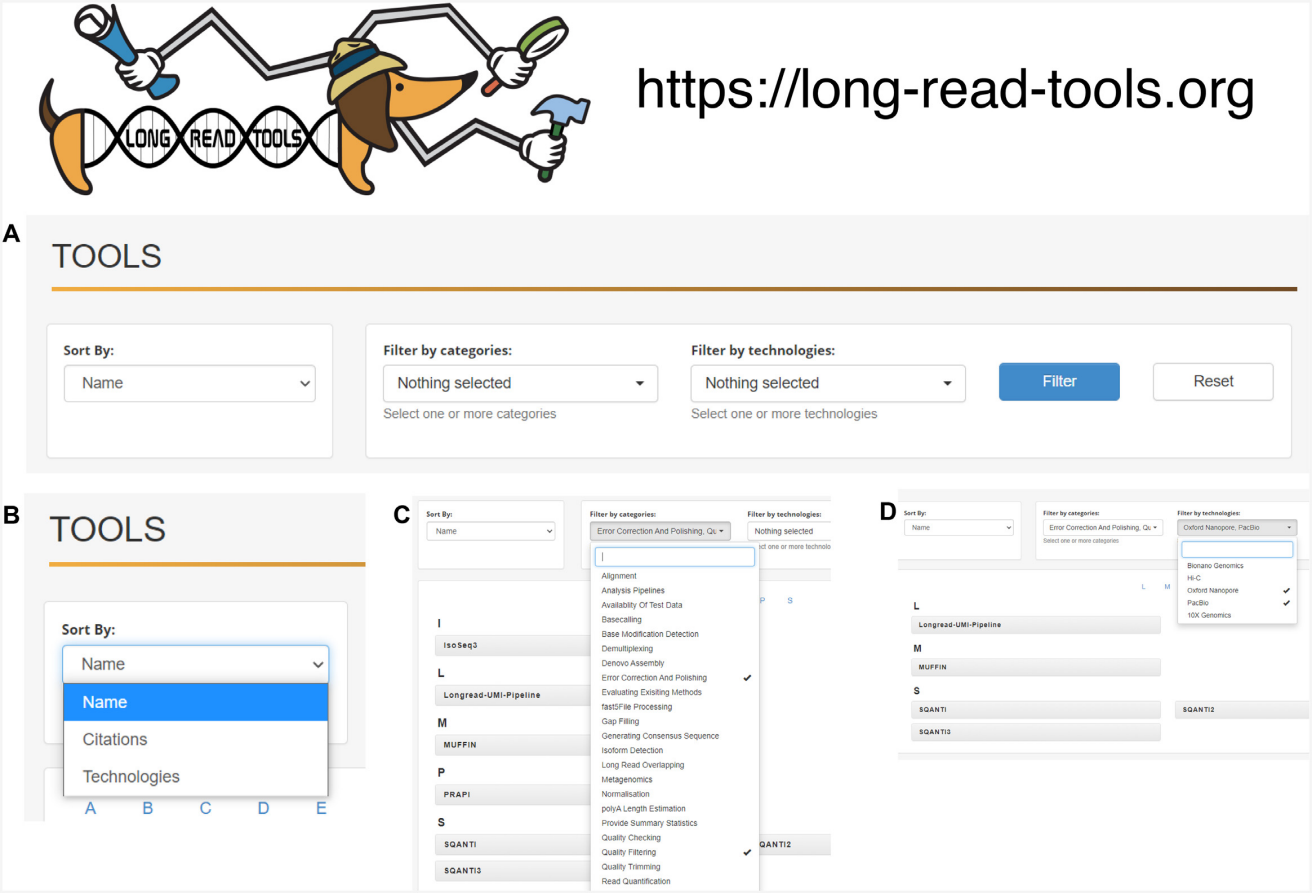
Example use of the Tools tab from long-read-tools.org. A. The custom toolbar for the page. B. Drop-down “Sort By" menu. C. Drop-down “Filter by categories" menu, which allows users to select multiple options by clicking on an item or typing the word in the text box. D. Drop-down “Filter by technologies" menu, which allows users to select multiple options by clicking on an item or typing the word in the text box. When multiple categories or technologies are selected, the website returns the intersection, not the union; i.e., a tool has to satisfy all the requirements to be reported.

Next is the Tools tab (Fig. 1A), which is the most important section of the database. This tab contains individual details on each software package (e.g., name, description, publication information, number of citations, location of the source code) and is intuitive to navigate.

If a user needs to sort through software tools by name, number of citations, or technology, one of these options can be selected from the drop-down menu in the left-hand corner, which will reorder the tools according to the selected parameter (Fig. 1B). This sort function can be used on its own or together with the filtering drop-down menus in the middle and the right-hand side of the page.

The filtering options allow the user to select multiple items from each of the filtering criteria (i.e., categories and technology) and will report the intersection. The union would be obtained by separate individual searches. For example, if the user wants to identify tools that can do both “error correction and polishing” and “quality filtering,” either typing them in the keyword box or clicking on the category item and pressing the filter option will show the filtered subset of tools (Fig. 1C). Only 7 tools match these criteria; all are pipelines rather than software dedicated to a unique task, as expected for the intersection of error correction and quality filtering functionalities. Of note, SQANTI1 and 2 are superseded by SQANTI3 [13], which is indicated when accessing the tools’ details. The user can subset these findings further on the basis of their preferred technology. Selecting Oxford Nanopore and PacBio returns the tools that are confirmed to work with both, thus removing PRAPI and IsoSeq3, which are specialized for PacBio data (Fig. 1D). However we note that a tool that has only been tested on 1 technology, and is thus annotated only with 1, may well be applicable to another given the similarities in data characteristics between long-read platforms.

**Figure 2:**
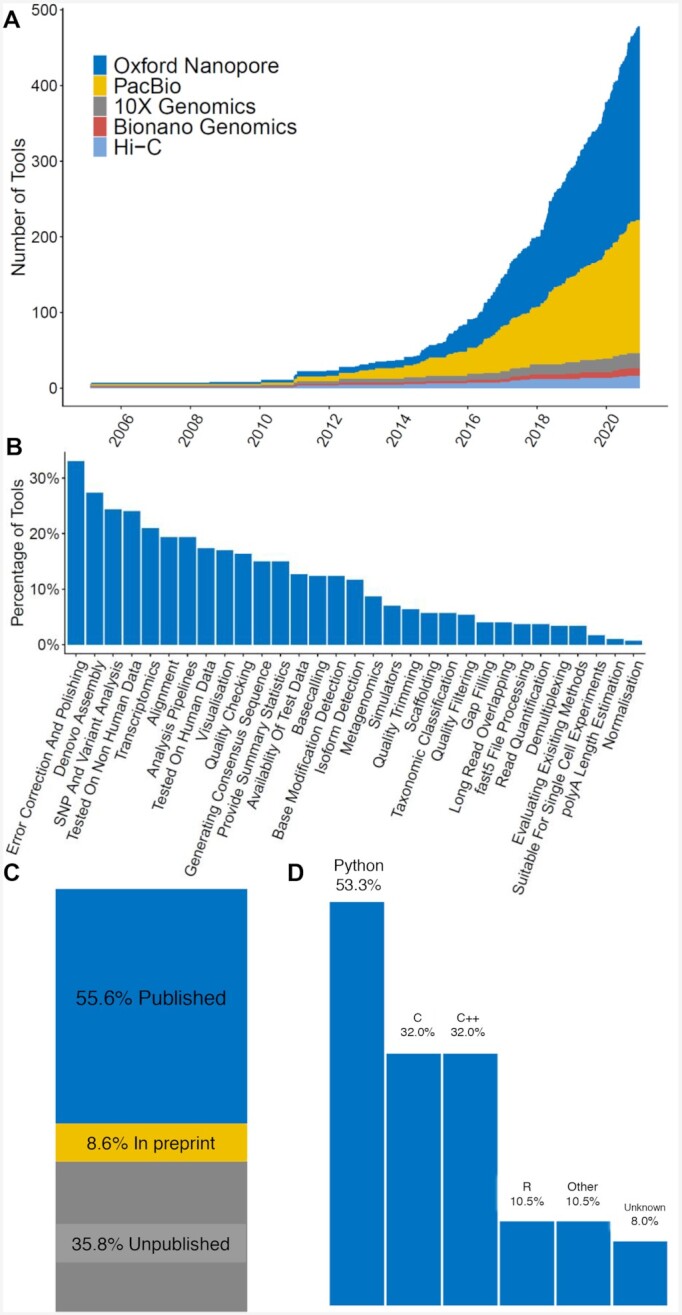
Summary statistics from long-read-tools.org. A. The number of tools released over time stratified by the long-read technologies they serve. B. The data analysis categories covered by the catalogued tools (ordered from most to least frequent). C. Publication status of the catalogued tools. D. The programming platforms used by the catalogued tools (ordered from most to least frequent). All languages making up ≥10% of a tool’s code are reported. These summary plots are available from the Statistics tab of the database website and can be easily exported for reuse. SNP: single-nucleotide polymorphism.

The Statistics tab contains summary plots obtained from an analysis of the information contained within the database (Fig. 2, e.g., growth in tool development over time, the distribution of tools across analysis tasks, publication status, summary of the programming languages they use).

The Submit tab is where the user can provide new information to the database if they have a tool to submit or modify.

The final tabs (Updates, FAQ and Contact Us) provide a summary of the social media activity of @long_read_tools (Twitter), answers to frequently asked questions, and a form to contact the database creators to ask general questions, respectively.

### Database statistics

long-read-tools.org contained 478 tools at the time of manuscript submission (Fig. 2A). These include 229, 155, 20, 15, and 10 tools that can handle ONT, PacBio, 10x, Hi-C, and Bionano Genomics data, respectively.

Tools began to appear in publications from the year 2005, although these were not targeted to long-read sequence analysis at that time. Tools focused on short-read alignment such as Gmap [14], SOAPdenovo [15], and STAR [16] have made alterations to their algorithms to support error-prone long-read sequence alignments. Nevertheless, short-read aligners have also been tested for their ability to work with long reads [17].

Tools specifically focused on long-read sequence analysis became available from 2012, following the commercial release of the PacBio RS sequencer in 2011 (see, e.g., PBcR [18,19] and LSC [20]). The ONT MinION was commercially released in 2014, and Poretools was published in the same year [21].

Available tools are categorized into 32 different functions (Fig. 2B). Of these, “error correction and polishing” and “*de novo* assembly” are the most common. On the other hand, “polyA length estimation,” “suitable for single cell experiments,” and “normalization” have the fewest tools, which highlights areas for further research and tool development.

It is also exciting to see that the majority of the tools have been published in either a peer-reviewed journal or on a preprint server (Fig. 2C). Moreover, tools written in Python outnumber tools implemented in other programming languages (Fig. 2D).

**Figure 3:**
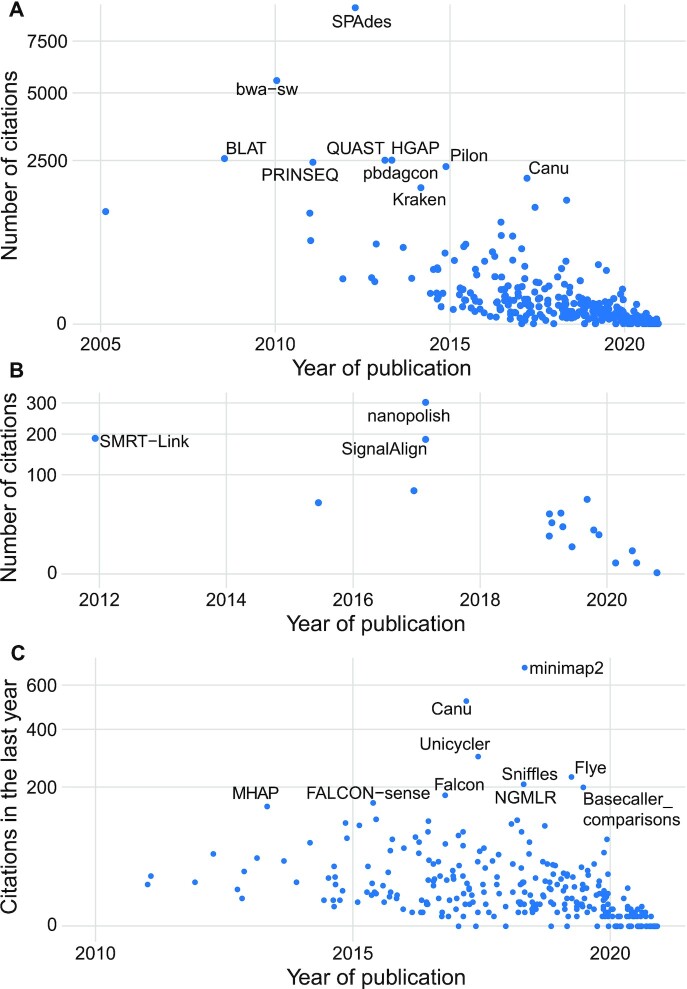
Popularity of the tools from long-read-tools.org based on publication citations. A. Across the entire database. B. For base modification detection. C. Across the entire database for citations in the past year. Each panel shows the year of publication on the x-axis and the square root of the number of citations on the y-axis. If the input set of tools is >50, the 10 most cited tools are labeled, otherwise the 3 most cited tools are labeled.

In terms of the number of citations, SPAdes [22] and bwa-sw [23] lead the pack (Fig. 3A). However, it should be noted that these tools existed before long-read technologies were popular, and most of these citations will therefore not reflect their popularity in long-read data analysis. The number of citations provides a more accurate indicator of usage for the tools that are unique to long-read analyses (e.g., nanopolish [24], SMRT-Link [25], and SignalAlign [26]) in “base modification detection” (Fig. 3B). To better capture the popularity of tools in a rapidly moving field, we also report the number of citations in the past year (Fig. 3C). For instance it can be observed that the Flye assembler [27] has been highly cited in the past 12 months despite its recent publication date (April 2019).

## Summary and Future Work

long-read-tools.org is an up-to-date, user-friendly catalogue that allows efficient searching of software by analysis category. It provides a comprehensive resource for new users to quickly and easily identify the relevant tools for their long-read data type and desired application. Our database illustrates the main areas of focus for existing tools, as well as the lack of software available in other areas (e.g., transcriptomics).

Other bioinformatic fields have experienced a similar growth in the number of available tools, prompting efforts to collate and organize them. These efforts vary from simple spreadsheets that list resources for the analysis of genomic repeats [28], through clickable lists of single-cell data analysis tools hosted on GitHub [29], all the way to dedicated websites offering search functions and statistics, such as scRNA-tools [30], which indexes tools for single-cell transcriptomics.

For long-read data, the long-read-catalog GitHub page [31] collects 40 tools for the analysis of ONT and PacBio data but it has not been updated in the past year. The Bioinformatics-Workflow-Frameworks-Platforms Google Sheets [32] list, among many other things, 84 tools relating to ONT data and 82 applicable to PacBio data. long-read-tools.org is both more comprehensive and easier to navigate than these databases.

We intend to keep increasing the breadth and depth of long-read-tools.org, but this should not come at the cost of making the database overwhelming to browse. Tutorials such as the “Long-read, long reach Bioinformatics Tutorials" website [33] are helpful in understanding how multiple tools fit into an analysis pipeline. Therefore we are focusing current efforts on facilitating the identification of best practices, validated workflows, and each tool’s relative strengths and weaknesses. Four additional entries are already available at tool submission and will be progressively populated: Underlying Algorithms, Underlying Assumptions, Strengths and Weaknesses, and Overall Performance. Furthermore a Tutorials tab highlighting common validated workflows and a Benchmarks tab featuring benchmarking studies and their results are in development.

## Availability of Source Code and Requirements

- Project name: long-read-tools.org database
- Project home page: long-read-tools.org
- Source code availability: https://github.com/shaniAmare/long_read_tools
- Operating system(s): Platform independent
- Programming language(s): R/JavaScript/html
- Other requirements: Accessible via any modern web browser
- License: MIT
- SciCrunch RRID:SCR_019116
- Biotools ID: biotools:long-read-tools

long-read-tools.org is a community effort, and we encourage researchers to contribute relevant tools, benchmarks, tutorials, and improvements to the database via the Submit tab.

## Data Availability

An archival copy of the code is available via the *GigaScience* database GigaDB [34].

## Abbreviations

10x: 10x Genomics; JSON: JavaScript Object Notation; ONT: Oxford Nanopore Technologies; PacBio: Pacific Biosciences;

## Competing Interests

The authors declare that they have no competing interests.

## Funding

This work was supported by funding from the Chan Zuckerberg Initiative DAF, an advised fund of Silicon Valley Community Foundation (grant No. 2019-002443 to MER), a fellowship from the Australian National Health and Medical Research Council (NHMRC, grant No. GNT1104924 to MER), Victorian State Government Operational Infrastructure Support, and Australian Government NHMRC IRIISS.

## Authors’ Contributions

S.L.A. structured the database; developed, implemented, and populated it; and wrote the manuscript. M.E.R. guided the research and wrote the manuscript. Q.G. structured the database, populated and validated entries, and wrote the manuscript. All authors read and approved the final manuscript.

## Supplementary Material

giab003_GIGA-D-20-00269_Original_Submission

giab003_GIGA-D-20-00269_Revision_1

giab003_GIGA-D-20-00269_Revision_2

giab003_GIGA-D-20-00269_Revision_3

giab003_Response_to_Reviewer_Comments_Original_Submission

giab003_Response_to_Reviewer_Comments_Revision_1

giab003_Response_to_Reviewer_Comments_Revision_2

giab003_Reviewer_1_Report_Original_Submission Pierre Morisse, Ph.D -- 9/11/2020 Reviewed

giab003_Reviewer_2_Report_Original_Submission Glennis Logsdon, Ph.D. -- 9/29/2020 Reviewed
