# Supplementary material for: long-read-tools.org: an interactive catalogue of analysis methods for long-read sequencing data
Source: Gigascience. 2021 Feb 16;10(2):giab003. doi: 10.1093/gigascience/giab003 (PMC7931822; doi:10.1093/gigascience/giab003)
Supplement: giab003_GIGA-D-20-00269_Original_Submission [file giab003_giga-d-20-00269_original_submission.pdf]

GigaScience

long-read-tools.org: an interactive catalogue of analysis methods for long-read sequencing data

--Manuscript Draft--

**Manuscript Number:**	GIGA-D-20-00269
**Full Title:**	long-read-tools.org: an interactive catalogue of analysis methods for long-read sequencing data
**Article Type:**	Technical Note
**Funding Information:**	Chan Zuckerberg Initiative — Dr Matthew E Ritchie<br>Silicon Valley Community Foundation (2019-002443) — Dr Matthew E Ritchie<br>National Health and Medical Research Council (GNT1104924) — Dr Matthew E Ritchie<br>State Government of Victoria — Dr Matthew E Ritchie<br>National Health and Medical Research Council (IRIISS) — Dr Matthew E Ritchie
**Abstract:**	Background<br><br>The data produced by long-read third-generation sequencers have unique characteristics compared to short-read sequencing data, often requiring tailored analysis tools for tasks ranging from quality control to downstream processing. The rapid growth in software that address these challenges for different genomics applications are difficult to keep track of, which makes it hard for users to choose the most appropriate tool for their analysis goal, and for developers to identify areas of need and existing solutions to benchmark against.<br>Findings<br><br>We describe the implementation of long-read-tools.org, an open-source database that organises the rapidly expanding collection of long-read data analysis tools and allows its exploration through interactive browsing and filtering. The current database release contains 448 tools across 320 categories. Most tools are developed in Python and the most frequent analysis tasks include basecalling, de novo assembly, error-correction, quality checking/filtering, and isoform detection, while long-read single-cell data analysis and transcriptomics are areas with the fewest tools available.<br>Conclusion<br><br>Continued growth in the application of long-read sequencing in genomics research positions the long-read-tools.org database as an essential resource that allows researchers to keep abreast of both established and emerging software to help guide the selection of the most relevant tool for their analysis needs.
**Corresponding Author:**	Quentin Gouil<br>Walter and Eliza Hall Institute of Medical Research<br>Parkville, VIC AUSTRALIA
**Corresponding Author Secondary Information:**	
**Corresponding Author's Institution:**	Walter and Eliza Hall Institute of Medical Research
**Corresponding Author's Secondary Institution:**	
**First Author:**	Shanika L Amarasinghe
**First Author Secondary Information:**	
**Order of Authors:**	Shanika L Amarasinghe

Powered by Editorial Manager® and ProduXion Manager® from Aries Systems Corporation

	Matthew E Ritchie
	Quentin Gouil
**Order of Authors Secondary Information:**	
**Additional Information:**	
**Question**	**Response**
Are you submitting this manuscript to a special series or article collection?	No
**Experimental design and statistics**<br><br>Full details of the experimental design and statistical methods used should be given in the Methods section, as detailed in our Minimum Standards Reporting Checklist. Information essential to interpreting the data presented should be made available in the figure legends.<br><br>Have you included all the information requested in your manuscript?	No
If not, please give reasons for any omissions below.<br><br>as follow-up to "**Experimental design and statistics**<br><br>Full details of the experimental design and statistical methods used should be given in the Methods section, as detailed in our Minimum Standards Reporting Checklist. Information essential to interpreting the data presented should be made available in the figure legends.<br><br>Have you included all the information requested in your manuscript?<br><br>"	no statistics applicable
**Resources**<br><br>A description of all resources used, including antibodies, cell lines, animals and software tools, with enough	No

information to allow them to be uniquely identified, should be included in the Methods section. Authors are strongly encouraged to cite Research Resource Identifiers (RRIDs) for antibodies, model organisms and tools, where possible.<br><br>Have you included the information requested as detailed in our Minimum Standards Reporting Checklist?	
If not, please give reasons for any omissions below.<br><br>as follow-up to "**Resources**<br><br>A description of all resources used, including antibodies, cell lines, animals and software tools, with enough information to allow them to be uniquely identified, should be included in the Methods section. Authors are strongly encouraged to cite Research Resource Identifiers (RRIDs) for antibodies, model organisms and tools, where possible.<br><br>Have you included the information requested as detailed in our Minimum Standards Reporting Checklist?<br><br>"	not applicable
**Availability of data and materials**<br><br>All datasets and code on which the conclusions of the paper rely must be either included in your submission or deposited in publicly available repositories (where available and ethically appropriate), referencing such data using a unique identifier in the references and in the "Availability of Data and Materials" section of your manuscript.<br><br>Have you have met the above requirement as detailed in our Minimum	Yes

Powered by Editorial Manager® and ProduXion Manager® from Aries Systems Corporation

Standards Reporting Checklist?	

Powered by Editorial Manager® and ProduXion Manager® from Aries Systems Corporation

Click here to access/download;LaTeX - Main Document (TeX file);main.tex

```
This is pdfTeX, Version 3.14159265-2.6-1.40.19 (TeX Live 2018/W32TeX)
(preloaded format=pdflatex 2018.7.12)  4 SEP 2020 05:35
entering extended mode
 restricted \write18 enabled.
 %&-line parsing enabled.
**main.tex
(./main.tex
LaTeX2e <2018-04-01> patch level 5

! LaTeX Error: File `oup-contemporary.cls' not found.

Type X to quit or <RETURN> to proceed,
or enter new name. (Default extension: cls)

Enter file name: 
! Emergency stop.
<read *> 
         
l.2 ^^M
       
*** (cannot \read from terminal in nonstop modes)


Here is how much of TeX's memory you used:
 10 strings out of 492646
 215 string characters out of 6133325
 56709 words of memory out of 5000000
 3994 multiletter control sequences out of 15000+600000
 3640 words of font info for 14 fonts, out of 8000000 for 9000
 1141 hyphenation exceptions out of 8191
 10i,0n,8p,61b,8s stack positions out of 5000i,500n,10000p,200000b,80000s
!  ==> Fatal error occurred, no output PDF file produced!
```

Reference PDF Click here to access/download;Manuscript;long_read_tools.pdf

GigaScience, 2020, 1–5

doi: xx.xxxx/xxxx
Manuscript in Preparation
Technical Note

TECHNICAL NOTE

long-read-tools.org: an interactive catalogue of analysis methods for long-read sequencing data

Shanika L. Amarasinghe [1,2*], Matthew E. Ritchie [1,2,3] and Quentin Gouil [1,2*]

[1]Epigenetics and Development Division, The Walter and Eliza Hall Institute of Medical Research, 1G Royal Parade, Parkville, Victoria 3052, Australia and [2]Department of Medical Biology, The University of Melbourne, Parkville, Victoria 3010, Australia and [3]School of Mathematics and Statistics, The University of Melbourne, Parkville, Victoria 3010, Australia

*amarasinghe.s@wehi.edu.au; gouil.q@wehi.edu.au

Abstract

Background The data produced by long-read third-generation sequencers have unique characteristics compared to short-read sequencing data, often requiring tailored analysis tools for tasks ranging from quality control to downstream processing. The rapid growth in software that address these challenges for different genomics applications are difficult to keep track of, which makes it hard for users to choose the most appropriate tool for their analysis goal, and for developers to identify areas of need and existing solutions to benchmark against.
Findings We describe the implementation of long-read-tools.org, an open-source database that organises the rapidly expanding collection of long-read data analysis tools and allows its exploration through interactive browsing and filtering. The current database release contains 448 tools across 320 categories. Most tools are developed in Python and the most frequent analysis tasks include basecalling, *de novo* assembly, error-correction, quality checking/filtering, and isoform detection, while long-read single-cell data analysis and transcriptomics are areas with the fewest tools available.
Conclusion Continued growth in the application of long-read sequencing in genomics research positions the long-read-tools.org database as an essential resource that allows researchers to keep abreast of both established and emerging software to help guide the selection of the most relevant tool for their analysis needs.

Key words: database; long-read sequencing; data analysis; nanopore; PacBio

Background

Long-read sequencing technologies facilitate versatile exploration of genomes owing to their ability to generate reads spanning several thousand base pairs [1]. Long reads can be *de novo* assembled or mapped to a reference to identify complicated structural variants and novel or complete transcripts that may otherwise be difficult to distinguish with short-read sequencing [2, 3, 4]. Improvements in throughput, error and cost reduction as well as increased interest in tool development for downstream data analyses [5] all contribute to the broadening adoption of long-read data across research fields.

To keep up with the rapid growth in software for long-read analysis, we collated and categorised existing long-read analysis tools at long-read-tools.org. This database enables easy navigation of the available software, allowing users to filter by specific tasks to identify methods that suit their analysis objectives.

Findings

Compiled on: September 4, 2020.
Draft manuscript prepared by the author.

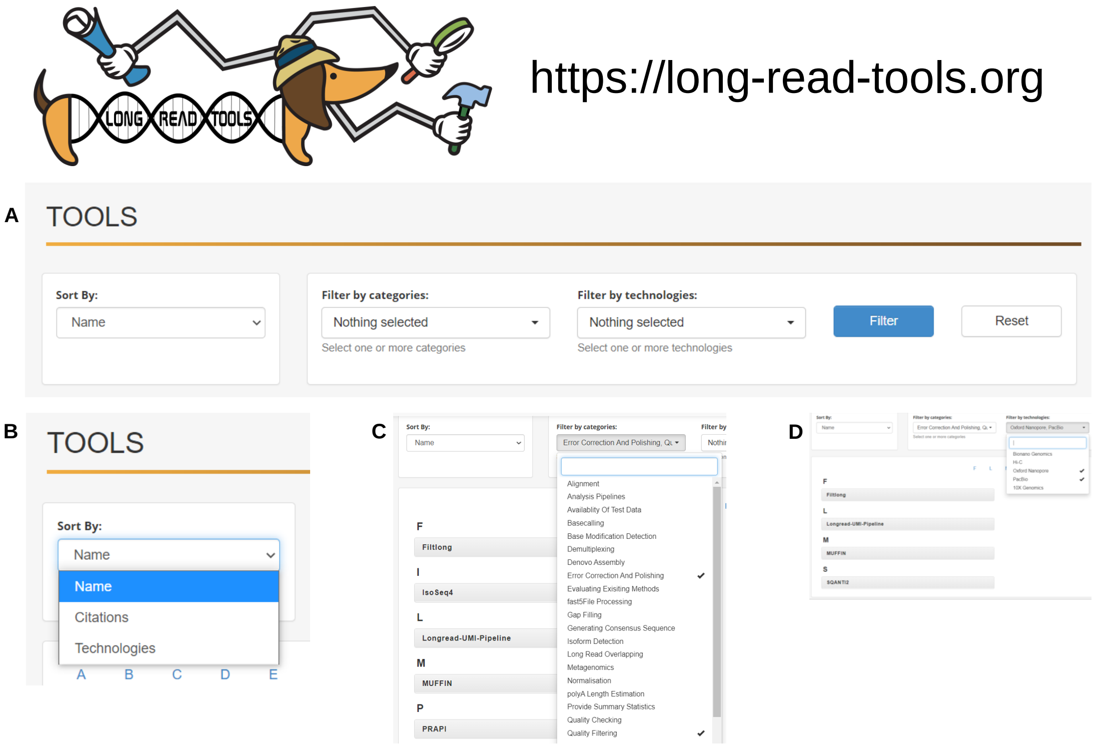

Figure 1. Example use of the `Tools` tab from long-read-tools.org A. The custom toolbar for the page. B. Drop-down *Sort By* menu. C. Drop down *Filter by categories* menu which allows users to select multiple options by clicking on an item or typing the word in the text box. D. Drop-down *Filter by technologies* menu which allows users to select multiple options by clicking on an item or typing the word in the text box.

Data Collection, Database Design & Implementation

The long-read-tools.org database is specifically designed to catalogue analysis tools for long-reads generated from genuine (PacBio and ONT) and synthetic (e.g. Hi-C, 10x, Bionano Genomics) long-read technologies. Up-to-date data is collected from various sources including publications, preprints, social media posts, mining public (GitHub, PyPI, Anaconda, CRAN, Bioconductor) and private repositories and via the tool submissions form accessible from the `Submit` tab.

The data collected in the form of a `.csv` file is processed within the R environment [6]. In this `.csv` file, each tool is categorised with a `TRUE` or `FALSE` value based on its functionality and technology(ies) in focus. Available details of the tools such as the description, publication status, tool licence and programming language are retrieved and stored. Furthermore, the number of citations for each tool is also retrieved via rcrossref (v.1.0.0) [7] and stored, serving as an indication of the popularity of the tool. Information on arXiv preprints is retrieved through the arXiv package (v.0.5.19) [8]. Multiple `JSON` files are generated during the processing step to populate the website. If publicly available, a tool's source code is checked to assess the current status of its code base (e.g. actively maintained or deprecated).

Several analysis-style plots are created to be displayed on the database as well. The original `.csv` input is processed to extract details such as the number of tools across time, the distribution of tools across categories, publication status and the main programming platforms used in tools development to summarise the contents of the database. The plots are created in the R environment using several main packages such as `ggplot2` (v.3.3.2) [9] and `plotly` (v.4.9.2) [10].

Database Usage

The long-read-tools.org website consists of several tabs, the first of which is the landing page (`Home`) that provides a summary of the database. The second `Table` tab contains the primary table with information that can be filtered using the search bar on the right. This tab can be used to view and download the required details of the complete database or a set of tools of interest.

Next is the `Tools` tab (Figure 1A), which is the most important section of the database. This tab contains individual details on each software package (e.g. name, description, publication information, number of citations, location of the source code, etc.) and is intuitive to navigate.

If a user requires to sort through software tools by name, number of citations, or technology, one of these options can be selected from the drop-down menu in the left hand corner, which will re-order the tools according to the selected parameter (Figure 1B). This sort function can be used on its own or together with the filtering drop down menus in the middle and the right hand side of the page.

The filtering options allow the user to select multiple items from each of the filtering criteria (i.e. categories and technology). For example, if the user wants to identify tools that can do both "error correction and polishing" and "quality filtering", either typing them in the keyword box or clicking on the category item and pressing the filter option will show the filtered subset of tools (Figure 1C). The user can subset these findings further based on their preferred technology (Figure 1D). For instance, it is clear that all the resulting tools from the above use case can work with PacBio data while only a subset work with ONT data.

The `Statistics` tab contains summary plots obtained from an analysis of the information contained within the database (Figure 2, e.g. growth in tool development over time, the distri-

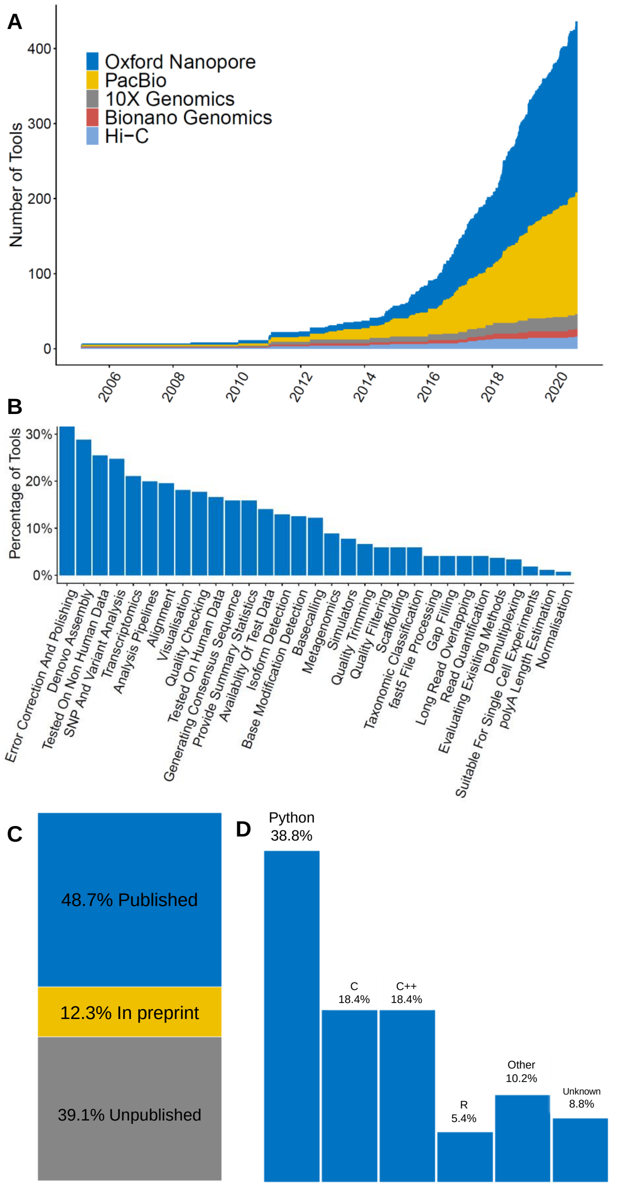

Figure 2. Summary statistics from long-read-tools.org A. The number of tools released over time stratified by the long-read technologies they serve. B. The data analysis categories covered by the catalogued tools (ordered from most to least frequent). C. Publication status of the catalogued tools. D. The programming platforms used by the catalogued tools (ordered from most to least frequent). These summary plots are available from the `Statistics` tab of the database website and can be easily exported for reuse.

bution of tools across analysis tasks, publication status, summary of the programming languages they use, etc.).

The `Submit` tab is where the user can provide new information to the database if they have a tool to submit or modify.

The final tabs (`Updates`, `FAQs` and `Contact Us`) provide a summary of the social media activity of *@long_read_tools* (Twitter), answers to frequently asked questions and a form to contact the database creators to ask general questions, respectively.

Database Statistics

Long-read-tools.org contains 448 tools at the time of manuscript submission (Figure 2A). These include 215, 144, 19, 15 and 10 tools that can handle ONT, PacBio, 10x, Hi-C, Bionano Genomics data, respectively.

Tools began to appear in publications from the year 2005, although these were not targeted to long-read sequence analysis at that time. Tools focused on short-read alignment such as *Gmap* [11], *Soap-denovo* [12] and *STAR* [13] have made alterations to their algorithms in order to support error-prone long-read sequence alignments. Nevertheless, short-read aligners have also been tested for their ability to work with long reads [14].

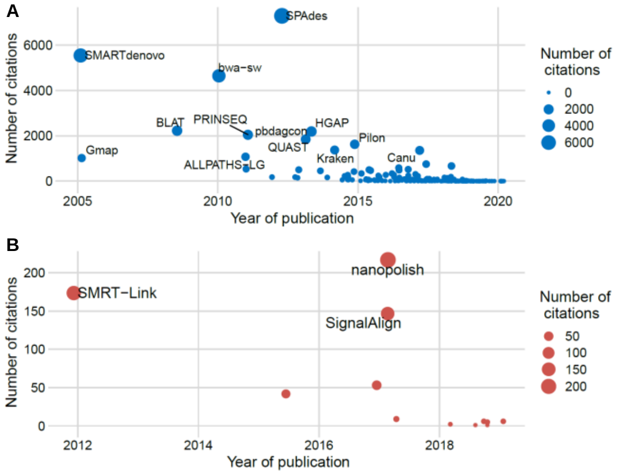

Figure 3. Popularity of the tools from long-read-tools.org based on publication citations A. Across the entire database. B. For *base modification detection*.

Tools specifically focused on long-read sequence analysis became available around 2013, coinciding with the commercial release of PacBio Biosciences and Oxford Nanopore Technologies [15] sequencing platforms.

Available tools are categorised into 32 different functions (Figure 2B). Of these, "error correction and polishing" and "*de novo* assembly" are the most common. On the other hand, "polyA length estimation", "suitable for single cell experiments" and "normalisation" have the lowest number of tools, which highlights areas for further research and tool development.

It is also exciting to see the majority of the tools have been published in either a peer-reviewed journal or on a preprint server (Figure 2C). Moreover, the number of tools written in Python outnumber tools implemented in other programming languages (Figure 2D).

In terms of the number of citations, *SPAdes* [16], *SMARTDenovo* [17] and *bwa-sw* [18] lead the pack (Figure 3A). However, it should be noted that these tools have been in existence before long-read technologies were popular, and most of these citations will therefore not reflect their popularity in long-read data analysis. The number of citations provides a more accurate indicator of usage for the tools that are unique to long-read analyses (e.g. *nanopolish* [19], *SMRT-Link* [20] and `SignalAlign` [21] in "base modification detection" (Figure 3B).

Summary and Future Work

Long-read-tools.org is an an up-to-date, user-friendly catalogue that allows efficient searching of software by analysis category. It provides a comprehensive resource for new users to quickly and easily identify the relevant tools for their long-read data type and desired application. Our database illustrates the main areas of focus for existing tools, as well as the lack of software available in other areas (e.g. transcriptomics).

Other bioinformatics fields have experienced a similar growth in the number of available tools, prompting efforts to collate and organise them. These efforts vary from simple spreadsheets that list resources for the analysis of genomic re-

peats [22], through clickable lists of single-cell data analysis tools hosted on GitHub [23], all the way to dedicated websites offering search functions and statistics, such as scRNA-tools [24] that indexes tools for single-cell transcriptomics.

For long-read data, the long-read-catalog GitHub page [25] collects 40 tools for the analysis of ONT and PacBio data but it has not been updated in the last year. The Bioinformatics-Workflow-Frameworks-Platforms Google Sheets [26] list, among many other things, 84 tools relating to ONT data and 82 applicable to PacBio data. long-read-tools.orgis both more comprehensive and easier to navigate than these databases.

We intend to keep increasing the breadth and depth of long-read-tools.org, but this should not come at the cost of making the database overwhelming to browse. Tutorials such as the Long-read, long reach Bioinformatics Tutorials website [27] are helpful in understanding how multiple tools fit into an analysis pipeline. Therefore we are focusing current efforts on facilitating the identification of best practices, validated workflows, and each tool's relative strengths and weaknesses. Four additional entries are already available at tool submission and will be progressively populated: Underlying Algorithms, Underlying Assumptions, Strengths and Weaknesses, and Overall Performance. Furthermore a `Quick-start` tab highlighting common validated workflows and a `Benchmarks` tab featuring benchmarking studies and their results are in development.

Availability of Source Code and Requirements

- Project name: Long-read-tools.org database
- Project home page: long-read-tools.org
- Source code availability: https://github.com/shaniAmare/long_read_tools
- Operating system(s): Platform independent
- Programming language(s): R/JavaScript/html
- Other requirements: Accessible via any modern web browser
- License: MIT

long-read-tools.org is a community effort, and we encourage researchers to contribute relevant tools and improvements to the database via the `Submit` tab.

Declarations

List of Abbreviations

ONT : Oxford Nanopore Technologies
PacBio : Pacific Biosciences
Hi-C : An extension of chromosomal interactions using chromosome conformation capture (3C)
10x : 10x Genomics

Competing Interests

The authors declare that they have no competing interests.

Funding

This work was supported by funding from the Chan Zuckerberg Initiative DAF, an advised fund of Silicon Valley Community Foundation (grant number 2019-002443 to MER), a fellowship from the Australian National Health and Medical Research Council (NHMRC, grant number GNT1104924 to MER), Victorian State Government Operational Infrastructure Support and Australian Government NHMRC IRIISS.

Authors' Contributions

SLA structured the database, developed, implemented and populated it and wrote the manuscript. MER guided the research and wrote the manuscript. QG validated the database entries, guided its population and wrote the manuscript. All authors read and approved the final manuscript.

Acknowledgements

We thank Dr. Luke Zappia, the main developer of the scRNA-tools.org database that this work builds upon, for his support in the initial stages of this project, Ms. Xueyi Dong and Mr. Shian Su for providing constructive feedback on the database, Mr. Sujith S. Waduge, Mr. Isuru Palliyaguru and Mr. Jithendra Sirimanne for their guidance in making the JavaScript underlying the database visualisation more reproducible and user-friendly and Ms. Tamara Mclennan and Ms. Ellen Conti for creating the database logo.

References

1. Logsdon GA, Vollger MR, Eichler EE. Long-read human genome sequencing and its applications. Nature Reviews Genetics 2020 jun;p. 1–18. https://doi.org/10.1038/s41576-020-0236-x.
2. Sakamoto Y, Sereewattanawoot S, Suzuki A. A new era of long-read sequencing for cancer genomics. Journal of Human Genetics 2020 jan;65(1):3–10. https://doi.org/10.1038/s10038-019-0658-5.
3. Ho SS, Urban AE, Mills RE. Structural variation in the sequencing era. Nature Reviews Genetics 2020 mar;21(3):171–189. https://doi.org/10.1038/s41576-019-0180-9.
4. Mitsuhashi S, Matsumoto N. Long-read sequencing for rare human genetic diseases. Journal of Human Genetics 2020 jan;65(1):11–19. https://doi.org/10.1038/s10038-019-0671-8.
5. Pollard MO, Gurdasani D, Mentzer AJ, Porter T, Sandhu MS. Long reads: their purpose and place. Human molecular genetics 2018 aug;27(R2):R234–R241. https://doi.org/10.1093/hmg/ddy177.
6. R Development Core Team. R: A Language and Environment for Statistical Computing. Vienna, Austria: R Foundation for Statistical Computing; 2012, http://www.R-project.org, ISBN 3-900051-07-0, http://www.R-project.org.
7. Chamberlain S, Zhu H, Jahn N, Boettiger C, Ram K. rcrossref: Client for Various 'CrossRef' 'APIs'; 2020, https://CRAN.R-project.org/package=rcrossref, r package version 1.0.0.
8. Ram K, Broman K. aRxiv: Interface to the arXiv API; 2019, https://CRAN.R-project.org/package=aRxiv, r package version 0.5.19.
9. Wickham H. ggplot2: Elegant Graphics for Data Analysis. Springer-Verlag New York; 2016. https://ggplot2.tidyverse.org.
10. Sievert C. Interactive Web-Based Data Visualization with R, plotly, and shiny. Chapman and Hall/CRC; 2020. https://plotly-r.com.
11. Wu TD, Watanabe CK. GMAP: a genomic mapping and alignment program for mRNA and EST sequences. Bioinformatics 2005 may;21(9):1859–1875. https://doi.org/10.1093/bioinformatics/bti310.
12. Luo R, Liu B, Xie Y, Li Z, Huang W, Yuan J, et al. SOAPdenovo2: an empirically improved memory-efficient short-

read de novo assembler. GigaScience 2012 dec;1(1):18. https://doi.org/10.1186/2047-217X-1-18.

13. Dobin A, Davis CA, Schlesinger F, Drenkow J, Zaleski C, Jha S, et al. STAR: ultrafast universal RNA-seq aligner. Bioinformatics 2013 jan;29(1):15–21. https://doi.org/10.1093/bioinformatics/bts635.
14. Krizanovic K, Echchiki A, Roux J, Sikic M. Evaluation of tools for long read RNA-seq splice-aware alignment. bioRxiv 2017 apr;p. 126656. https://doi.org/10.1101/126656.
15. Amarasinghe SL, Su S, Dong X, Zappia L, Ritchie ME, Gouil Q. Opportunities and challenges in long-read sequencing data analysis. Genome Biology 2020 feb;21(1):1–16. https://doi.org/10.1186/s13059-020-1935-5.
16. Bankevich A, Nurk S, Antipov D, Gurevich AA, Dvorkin M, Kulikov AS, et al. SPAdes: A New Genome Assembly Algorithm and Its Applications to Single-Cell Sequencing. Journal of Computational Biology 2012 may;19(5):455–477. https://doi.org/10.1089/cmb.2012.0021.
17. Ruan J, ruanjue/smartdenovo: Ultra-fast de novo assembler using long noisy reads;. https://github.com/ruanjue/smartdenovo, accessed on 26/08/2020.
18. Li H, Durbin R. Fast and accurate long-read alignment with Burrows-Wheeler transform. Bioinformatics 2010 jan;26(5):589–595. https://doi.org/10.1093/bioinformatics/btp698.
19. Loman NJ, Quick J, Simpson JT. A complete bacterial genome assembled de novo using only nanopore sequencing data. Nature Methods 2015 jul;12(8):733–735. https://github.com/jts/nanopolish/.
20. Ardui S, Ameur A, Vermeesch JR, Hestand MS. Single molecule real-time (SMRT) sequencing comes of age: Applications and utilities for medical diagnostics. Nucleic Acids Research 2018;46(5):2159–2168. https://doi.org/10.1093/nar/gky066.
21. Rand AC, Jain M, Eizenga JM, Musselman-Brown A, Olsen HE, Akeson M, et al. Mapping DNA methylation with high-throughput nanopore sequencing. Nature Methods 2017 feb;14(4):411–413. https://doi.org/10.1038/nmeth.4189.
22. Repeat_Resources - Google Sheets;. https://docs.google.com/spreadsheets/d/1UBK70zExiL0gFVaIAILiGhflCGXAq_SF_lymaxTE1pY/edit#gid=1266138738, accessed on 26/08/2020.
23. Davis S, seandavi/awesome-single-cell: Community-curated list of software packages and data resources for single-cell, including RNA-seq, ATAC-seq, etc.;. https://github.com/seandavi/awesome-single-cell, accessed on 26/08/2020.
24. Zappia L, Phipson B, Oshlack A. Exploring the single-cell RNA-seq analysis landscape with the scRNA-tools database. PLOS Computational Biology 2018 jun;14(6):e1006245. https://doi.org/10.1371/journal.pcbi.1006245.
25. Molecular Microbiology and Infection Unit, University of Lisbon, B-UMMI/long-read-catalog: catalog for long-read sequencing tools;. https://github.com/B-UMMI/long-read-catalog, accessed on 26/08/2020.
26. Vilella A, Bioinformatics-Workflow-Frameworks-Platforms.v6.6.6 - Google Sheets;. https://docs.google.com/spreadsheets/d/1plkAsT_S3CzSeb7ivxyjRnHyrK3JclUCXeUMf_azraY/edit#gid=471877065, accessed on 26/08/2020.
27. Kahlke T, Long-read, long read bioinformatics tutorials;. https://timkahlke.github.io/LongRead_tutorials/, accessed on 26/08/2020.
