# Supplementary material for: long-read-tools.org: an interactive catalogue of analysis methods for long-read sequencing data
Source: Gigascience. 2021 Feb 16;10(2):giab003. doi: 10.1093/gigascience/giab003 (PMC7931822; doi:10.1093/gigascience/giab003)
Supplement: giab003_GIGA-D-20-00269_Revision_1 [file giab003_giga-d-20-00269_revision_1.pdf]

// GigaScience

long-read-tools.org: an interactive catalogue of analysis methods for long-read sequencing data

--Manuscript Draft--

**Manuscript Number:**	GIGA-D-20-00269R1
**Full Title:**	long-read-tools.org: an interactive catalogue of analysis methods for long-read sequencing data
**Article Type:**	Technical Note
**Funding Information:**	Chan Zuckerberg Initiative — Dr Matthew E Ritchie<br>Silicon Valley Community Foundation (2019-002443) — Dr Matthew E Ritchie<br>National Health and Medical Research Council (GNT1104924) — Dr Matthew E Ritchie<br>State Government of Victoria — Dr Matthew E Ritchie<br>National Health and Medical Research Council (IRIISS) — Dr Matthew E Ritchie
**Abstract:**	Background<br><br>The data produced by long-read third-generation sequencers have unique characteristics compared to short-read sequencing data, often requiring tailored analysis tools for tasks ranging from quality control to downstream processing. The rapid growth in software that address these challenges for different genomics applications are difficult to keep track of, which makes it hard for users to choose the most appropriate tool for their analysis goal, and for developers to identify areas of need and existing solutions to benchmark against.<br>Findings<br><br>We describe the implementation of long-read-tools.org, an open-source database that organises the rapidly expanding collection of long-read data analysis tools and allows its exploration through interactive browsing and filtering. The current database release contains 478 tools across 32 categories. Most tools are developed in Python and the most frequent analysis tasks include basecalling, de novo assembly, error-correction, quality checking/filtering, and isoform detection, while long-read single-cell data analysis and transcriptomics are areas with the fewest tools available.<br>Conclusion<br><br>Continued growth in the application of long-read sequencing in genomics research positions the long-read-tools.org database as an essential resource that allows researchers to keep abreast of both established and emerging software to help guide the selection of the most relevant tool for their analysis needs.
**Corresponding Author:**	Quentin Gouil<br>Walter and Eliza Hall Institute of Medical Research<br>Parkville, VIC AUSTRALIA
**Corresponding Author Secondary Information:**	
**Corresponding Author's Institution:**	Walter and Eliza Hall Institute of Medical Research
**Corresponding Author's Secondary Institution:**	
**First Author:**	Shanika L Amarasinghe
**First Author Secondary Information:**	
**Order of Authors:**	Shanika L Amarasinghe

	Matthew E Ritchie
	Quentin Gouil
**Order of Authors Secondary Information:**	
**Response to Reviewers:**	Response to editor<br><br>Dear Dr Zauner,<br><br>We are pleased to submit a revised version of our manuscript “long-read-tools.org: an interactive catalogue of analysis methods for long-read sequencing data”. We have addressed all the reviewers’ comments and made a few additional improvements to the manuscript and database, as outlined in the response to reviewers. The changes to the manuscript pdf are marked in red.<br>We hope that you find the revised work suitable for publication in Gigascience.<br><br>Kind regards,<br><br>Quentin Gouil<br>Senior postdoctoral Researcher<br>Epigenetics and Development Division<br>Walter and Eliza Hall Institute<br><br><br><br>Response to reviewers<br><br>We thank the reviewers for their helpful and encouraging comments. In addition to our in-line response to each point, here is a summary of the changes to the manuscript and database:<br>- we registered long-read-tools.org on bio.tools (biotools:long-read-tools) and SciCrunch (RRID:SCR_019116)<br>- the “Benchmarks” and “Tutorials” tabs are now live and we hope they will make long-read-tools even more useful and easy to navigate.<br>- Programming language and license extraction from GitHub repos are now automated, avoiding mistakes and ameliorating formatting consistency<br>- The database now reports recent citations (from the past year) as well as total citations, making it easier to track a tool’s popularity. We have updated figure 3 with this data.<br>- We audited all the entries in the database to update DOIs for preprinted work that is now published, check the validity of the links to the code, and annotate deprecated tools.<br>- Having added a few more tools, the database contains 478 tools as of Dec 20th 2020.<br>- All figures and statistics have been updated to reflect the state of the database as of Dec 20th 2020.<br><br><br>Reviewer #1:<br>1) When on the "Tools" tab, if I click on a tool, then on one of its categories, I'm redirected to the "Tools" tab, but the chosen category is not taken into account.<br>The complete list of tools is thus displayed, and forces the user to re-enter the desired category.<br>I believe being able to access the tools related to a given category when clicking it from the description of a tool would improve user-friendliness.<br><br>We have changed this behaviour so that clicking on a Category within a tool now displays all the tools that fit in the Category, rather than all the tools without filtering. Thank you for suggesting this improvement.<br><br>2) Platforms sometimes appear to be wrong.<br>For instance, CONSENT, which I developed, is listed as "Python", when most of its code is written in C++.<br>Actually, the GitHub repository contains a Python script in its "bin" folder, but GitHub

Powered by Editorial Manager® and ProduXion Manager® from Aries Systems Corporation

does report that most of the code is written in C++.
I'm not exactly sure how the platform is inferred (By analyzing the bin folder? The language distribution indicated on GitHub? Manually?), and if other tools are affected, but if it is automatically inferred, I believe it should be verified to keep the database as precise as possible.

Assigning a Language to a tool was until now a manual process during tool submission. This process was prone to errors, as exemplified by the misclassification of CONSENT. In other cases only one language was reported when two or more contributed significantly. Thank you for bringing our attention to this problem. Upon submission of a new tool, if a GitHub repository is provided we now automatically fetch a repository's language distribution via the GitHub API, reporting all languages that amount to at least 10% of the code (excluding code like TeX or Makefiles). We then manually inspect and resolve any differences between the submission and the auto-retrieved data. We now also use the same process to retrieve a tool's license. Applying this process to the existing database entries allowed us to correct or enrich the language annotations for 138 tools in addition to CONSENT, and we have updated Figure 2D accordingly.

3) The authors state that "For instance, it is clear that all the resulting tools from the above use case can work with PacBio data while only a subset work with ONT data." However, it does not appear to be entirely clear why the tools would work on PacBio but not on Nanopore.
How is the distinction made exactly? Is it according to the experiments performed in the paper related to the tool, e.g. if it is tested only on PacBio data?
The PacBio and Nanopore reads share a common ground (long sequences, high error-rates), and I don't exactly understand why a given tool, especially for error correction which is underlined in the example, would work on PacBio but not on ONT.
I do understand that some tools were only evaluated on PacBio and perform better on PacBio data, e.g. because of the lower error-rate of PacBio reads compared to ONT, but I don't see why they would not work at all on ONT data.

We have clarified this paragraph. The statement only referred to the specific example of filtering shown in Fig1 C and D, where of the 5 pipelines that could do error correction and quality filtering, 2 were designed specifically for PacBio and not ONT. We have also added a caveat that in general, a tool's annotation with a single technology does not preclude its working with another.
The paragraph now reads:
"For example, if the user wants to identify tools that can do both "error correction and polishing" and "quality filtering", either typing them in the keyword box or clicking on the category item and pressing the filter option will show the filtered subset of tools (Figure 1C). Only seven tools match these criteria; all are pipelines rather than software dedicated to a unique task, as expected for the intersection of error correction and quality filtering functionalities. Of note, SQANTI1 and 2 are superseded by SQANTI3 [13], which is indicated when accessing the tools' details. The user can subset these findings further based on their preferred technology. Selecting Oxford Nanopore and PacBio returns the tools that are confirmed to work with both, thus removing PRAPI and IsoSeq3 that are specialised for PacBio data (Figure 1D). However
we note that a tool that has only been tested on one technology, and is thus annotated only with one, may well be applicable to another given the similarities in data characteristics between long-read platforms."

4) The authors mention that "Tools specifically focused on long-read sequence analysis became available around 2013, coinciding with the commercial release of PacBio Biosciences and Oxford Nanopore Technologies [15] sequencing platforms." However, tools specifically designed for long-reads actually stated to emerge in 2012, see for instance error-correction tools PBcR (http://doi.org/10.1038/nbt.2280) and LSC (http://doi.org/10.1371/journal.pone.0046679).

We have corrected this inaccurate statement, thank you for bringing it to our attention. The text now reads:
"Tools specifically focused on long-read sequence analysis became available from 2012, following the commercial release of the PacBio RS sequencer in 2011 (see for example PBcR [18, 19] and LSC [20]). The ONT MinION was commercially released in

Powered by Editorial Manager® and ProduXion Manager® from Aries Systems Corporation

2014, and Poretools was published in the same year [21].”

Typos / Bibliography remarks :

1) "Moreover, the number of tools written in Python outnumber tools implemented in other programming languages (Figure 2D)."
I believe this sentence should be rewritten, e.g. simply "Moreover, tools written in Python outnumber tools implemented in other programming languages".

This has been corrected.

2) "long-read-tools.orgis both more comprehensive and easier to navigate than these databases."
A space is missing between "long-read-tools.org" and "is"

Fixed.

3) Citation 6 (R Development Core Team. R: A Language and Environment for Statistical Computing. Vienna, Austria: R Foundation for Statistical Computing; 2012, http://www.R-project.org, ISBN 3-900051-07-0, http://www.R-project.org.)
The link to the R project is reported two times. I believe one of the links could be omitted.

Fixed.

4) Citation 19 (Loman NJ, Quick J, Simpson JT. A complete bacterial genome assembled de novo using only nanopore sequencing data. Nature Methods 2015 jul;12(8):733-735.)
The mentioned citation has a DOI (https://doi.org/10.1038/nmeth.3444). I believe it should be mentioned, instead of or in addition to the GitHub page, in order to maintain consistency with other citations of published papers/softwares.

Fixed, thank you.

Level of interest:An article of importance in its field
Quality of written English: Good
Statistical review:No, the manuscript does not need to be seen by a statistician.

Reviewer #2:
1. The authors state that the data is collected from publications, preprints, social media posts, and public and private repositories. Is this a manual effort, or is there a database crawler that's implemented to identify new tools? If it is manual, how will the authors ensure that the database is continually updated to reflect the latest tools?

Updating the database is still a manual process. In time we hope to develop a base of regular contributors from the community of users to ensure the longevity of the project. This model has so far been successful for the scRNA-tools database, created 3 years ago and now maintained by a team of volunteers around the world.

2. While the number of citations is a helpful indicator of widely-used tools, it can often be dominated by those tools that have been around longer than others and, therefore, can be misleading for those who want to use the latest and most helpful tools. One suggestion is to create a second metric that is the "number of citations within the last six months" (or one year—whichever is most practical), which can give the user an idea of tools that are gaining popularity as opposed to those that are becoming outdated. Another way to implement this idea would be to generate an interactive plot of the number of citations for each tool over time.

Thank you for the suggestions, we now report the number of citations in the past year, obtained through the citecorp R package. We have updated Figure 3 to illustrate this functionality and added a description in the Database implementation section.

Powered by Editorial Manager® and ProduXion Manager® from Aries Systems Corporation

	3. In searching the database myself, I found a number of tools that are either misnamed or missing:<br><br>Misnamed: Isoseq4 (this should be Isoseq3)<br>Missing: HiFiasm, GraphAligner, MosaicFlye, TandemFlye, TandemTools, Winnowmap<br><br>Each of the missing tools are on the cutting edge of genome assembly and quality assessment and have gained popularity in recent months. This underscores the necessity for a database crawler that is more comprehensive than manual cataloging.<br><br>We have made the corrections and additions that were raised, thank you.<br><br>4. I also found an instance where the same category is listed twice for one tool. The tool "minoTour" has the following categories listed with it: "Alignment, Alignment, Quality Checking, SNP And Variant Analysis, Visualisation, Provide Summary Statistics, Analysis Pipelines, Metagenomics". There should only be one instance of "Alignment".<br><br>Thank you for pointing this out, it has been corrected.<br><br>5. Are there any types of tools that are excluded from the database? If so, the article and website should provide a description of those that are not included.<br><br>We do not currently exclude any tools. We debated whether tools that are introduced in preprints without a link to the code should be excluded, but they are very rare cases so we include them with a warning in the description.<br><br>6. Typo in the abstract: "320" should be "32".<br><br>Fixed.
**Additional Information:**	
**Question**	**Response**
Are you submitting this manuscript to a special series or article collection?	No
**Experimental design and statistics**<br><br>Full details of the experimental design and statistical methods used should be given in the Methods section, as detailed in our Minimum Standards Reporting Checklist. Information essential to interpreting the data presented should be made available in the figure legends.<br><br>Have you included all the information requested in your manuscript?	No
If not, please give reasons for any omissions below.<br><br>as follow-up to "**Experimental design and statistics**	no statistics applicable

Full details of the experimental design and statistical methods used should be given in the Methods section, as detailed in our Minimum Standards Reporting Checklist. Information essential to interpreting the data presented should be made available in the figure legends.<br><br>Have you included all the information requested in your manuscript?<br><br>"	
**Resources**<br><br>A description of all resources used, including antibodies, cell lines, animals and software tools, with enough information to allow them to be uniquely identified, should be included in the Methods section. Authors are strongly encouraged to cite Research Resource Identifiers (RRIDs) for antibodies, model organisms and tools, where possible.<br><br>Have you included the information requested as detailed in our Minimum Standards Reporting Checklist?	No
If not, please give reasons for any omissions below.<br><br>as follow-up to "**Resources**<br><br>A description of all resources used, including antibodies, cell lines, animals and software tools, with enough information to allow them to be uniquely identified, should be included in the Methods section. Authors are strongly encouraged to cite Research Resource Identifiers (RRIDs) for antibodies, model organisms and tools, where possible.<br><br>Have you included the information	not applicable

requested as detailed in our Minimum Standards Reporting Checklist?<br><br>"	
**Availability of data and materials**<br><br>All datasets and code on which the conclusions of the paper rely must be either included in your submission or deposited in publicly available repositories (where available and ethically appropriate), referencing such data using a unique identifier in the references and in the "Availability of Data and Materials" section of your manuscript.<br><br>Have you have met the above requirement as detailed in our Minimum Standards Reporting Checklist?	Yes

LaTeX - Main Document (TeX file) Click here to access/download;LaTeX - Main Document (TeX file);main.tex

```
This is pdfTeX, Version 3.14159265-2.6-1.40.21 (TeX Live 2020/W32TeX)
(preloaded format=pdflatex 2020.5.12)  21 DEC 2020 22:14
entering extended mode
 restricted \write18 enabled.
 %&-line parsing enabled.
**main.tex
(./main.tex
LaTeX2e <2020-02-02> patch level 5
L3 programming layer <2020-05-05>

! LaTeX Error: File `oup-contemporary.cls' not found.

Type X to quit or <RETURN> to proceed,
or enter new name. (Default extension: cls)

Enter file name:
! Emergency stop.
<read *>

l.2 ^^M

*** (cannot \read from terminal in nonstop modes)


Here is how much of TeX's memory you used:
 22 strings out of 480681
 490 string characters out of 5908536
 236875 words of memory out of 5000000
 15943 multiletter control sequences out of 15000+600000
 532338 words of font info for 24 fonts, out of 8000000 for 9000
 1141 hyphenation exceptions out of 8191
 14i,0n,17p,90b,10s stack positions out of
5000i,500n,10000p,200000b,80000s
!  ==> Fatal error occurred, no output PDF file produced!
```

Manuscript

Click here to access/download;Manuscript;long_read_tools_org_GigaScienc

OXFORD (GIGA)^n SCIENCE

GigaScience, 2020, 1–5

doi: xx.xxxx/xxxx
Manuscript in Preparation
Technical Note

TECHNICAL NOTE

long-read-tools.org: an interactive catalogue of analysis methods for long-read sequencing data

Shanika L. Amarasinghe [1,2*], Matthew E. Ritchie [1,2,3] and Quentin Gouil [1,2*]

[1]Epigenetics and Development Division, The Walter and Eliza Hall Institute of Medical Research, 1G Royal Parade, Parkville, Victoria 3052, Australia and [2]Department of Medical Biology, The University of Melbourne, Parkville, Victoria 3010, Australia and [3]School of Mathematics and Statistics, The University of Melbourne, Parkville, Victoria 3010, Australia

*amarasinghe.s@wehi.edu.au; gouil.q@wehi.edu.au

Abstract

Background The data produced by long-read third-generation sequencers have unique characteristics compared to short-read sequencing data, often requiring tailored analysis tools for tasks ranging from quality control to downstream processing. The rapid growth in software that address these challenges for different genomics applications are difficult to keep track of, which makes it hard for users to choose the most appropriate tool for their analysis goal, and for developers to identify areas of need and existing solutions to benchmark against.
Findings We describe the implementation of long-read-tools.org, an open-source database that organises the rapidly expanding collection of long-read data analysis tools and allows its exploration through interactive browsing and filtering. The current database release contains 478 tools across 32 categories. Most tools are developed in Python and the most frequent analysis tasks include basecalling, *de novo* assembly, error-correction, quality checking/filtering, and isoform detection, while long-read single-cell data analysis and transcriptomics are areas with the fewest tools available.
Conclusion Continued growth in the application of long-read sequencing in genomics research positions the long-read-tools.org database as an essential resource that allows researchers to keep abreast of both established and emerging software to help guide the selection of the most relevant tool for their analysis needs.

Key words: database; long-read sequencing; data analysis; nanopore; PacBio

Background

Long-read sequencing technologies facilitate versatile exploration of genomes owing to their ability to generate reads spanning several thousand base pairs [1]. Long reads can be *de novo* assembled or mapped to a reference to identify complicated structural variants and novel or complete transcripts that may otherwise be difficult to distinguish with short-read sequencing [2, 3, 4]. Improvements in throughput, error and cost reduction as well as increased interest in tool development for downstream data analyses [5] all contribute to the broadening adoption of long-read data across research fields.

To keep up with the rapid growth in software for long-read analysis, we collated and categorised existing long-read analysis tools at long-read-tools.org. This database enables easy navigation of the available software, allowing users to filter by specific tasks to identify methods that suit their analysis objectives.

Findings

Compiled on: December 22, 2020.
Draft manuscript prepared by the author.

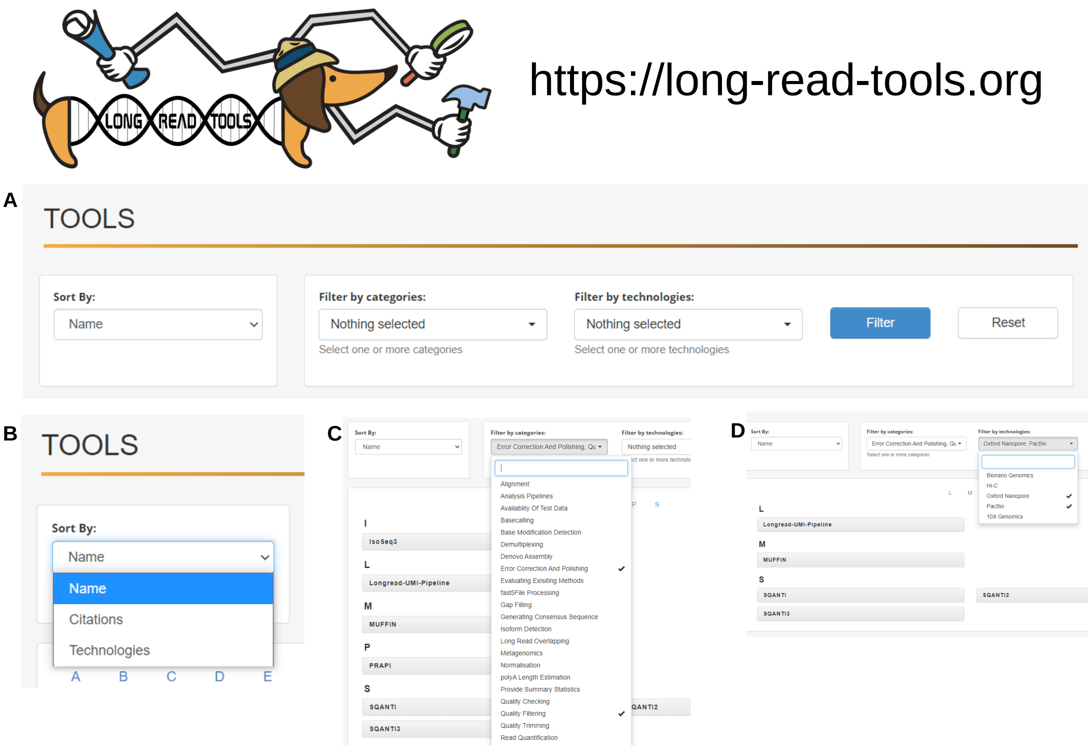

Figure 1. Example use of the `Tools` **tab from long-read-tools.org** A. The custom toolbar for the page. B. Drop-down *Sort By* menu. C. Drop-down *Filter by categories* menu which allows users to select multiple options by clicking on an item or typing the word in the text box. D. Drop-down *Filter by technologies* menu which allows users to select multiple options by clicking on an item or typing the word in the text box. When multiple categories or technologies are selected, the website returns the intersection, not the union, i.e. a tool has to satisfy all the requirements to be reported.

Data Collection, Database Design & Implementation

The long-read-tools.org database is specifically designed to catalogue analysis tools for long-reads generated from genuine (PacBio and ONT) and synthetic (e.g. Hi-C, 10x, Bionano Genomics) long-read technologies. Up-to-date data is collected from various sources including publications, preprints, social media posts, mining public (GitHub, PyPI, Anaconda, CRAN, Bioconductor) and private repositories and via the tool submissions form accessible from the `Submit` tab.

The data collected in the form of a `.csv` file is processed within the R environment [6]. In this `.csv` file, each tool is categorised with a `TRUE` or `FALSE` value based on its functionality and technology(ies) in focus. Available details of the tools such as the description, publication status, tool licence and programming language are retrieved and stored. Furthermore, the total number of citations for each tool is retrieved via rcrossref (v1.0.0) [7] and stored, while the number of citations from the past year is obtained through the citecorp R package (v0.3.0) [8] from the COCI database [9]. Both citation metrics may serve as an indication of a tool's popularity. Information on arXiv preprints is retrieved through the arXiv package (v0.5.19) [10]. Multiple `JSON` files are generated during the processing step to populate the website. If publicly available, a tool's source code is checked to assess the current status of its code base (e.g. actively maintained or deprecated).

Several analysis-style plots are created to be displayed on the database as well. The original `.csv` input is processed to extract details such as the number of tools across time, the distribution of tools across categories, publication status and the main programming platforms used in tools development to summarise the contents of the database. The plots are created in the R environment using several main packages such as `ggplot2` (v3.3.2) [11] and `plotly` (v4.9.2) [12].

Database Usage

The long-read-tools.org website consists of several tabs, the first of which is the landing page (`Home`) that provides a summary of the database. The second `Table` tab contains the primary table with information that can be filtered using the search bar on the right. This tab can be used to view and download the required details of the complete database or a set of tools of interest.

Next is the `Tools` tab (Figure 1A), which is the most important section of the database. This tab contains individual details on each software package (e.g. name, description, publication information, number of citations, location of the source code, etc.) and is intuitive to navigate.

If a user requires to sort through software tools by name, number of citations, or technology, one of these options can be selected from the drop-down menu in the left hand corner, which will re-order the tools according to the selected parameter (Figure 1B). This sort function can be used on its own or together with the filtering drop down menus in the middle and the right hand side of the page.

The filtering options allow the user to select multiple items from each of the filtering criteria (i.e. categories and technology) and will report the intersection. The union would be obtained by separate individual searches. For example, if the user wants to identify tools that can do both "error correction and polishing" and "quality filtering", either typing them in the keyword box or clicking on the category item and pressing the filter option will show the filtered subset of tools (Figure 1C). Only seven tools match these criteria; all are pipelines rather than software dedicated to a unique task, as expected for the intersection of error correction and quality filtering functionalities. Of note, *SQANTI1* and *2* are superseded by *SQANTI3* [13], which is indicated when accessing the tools' details. The user

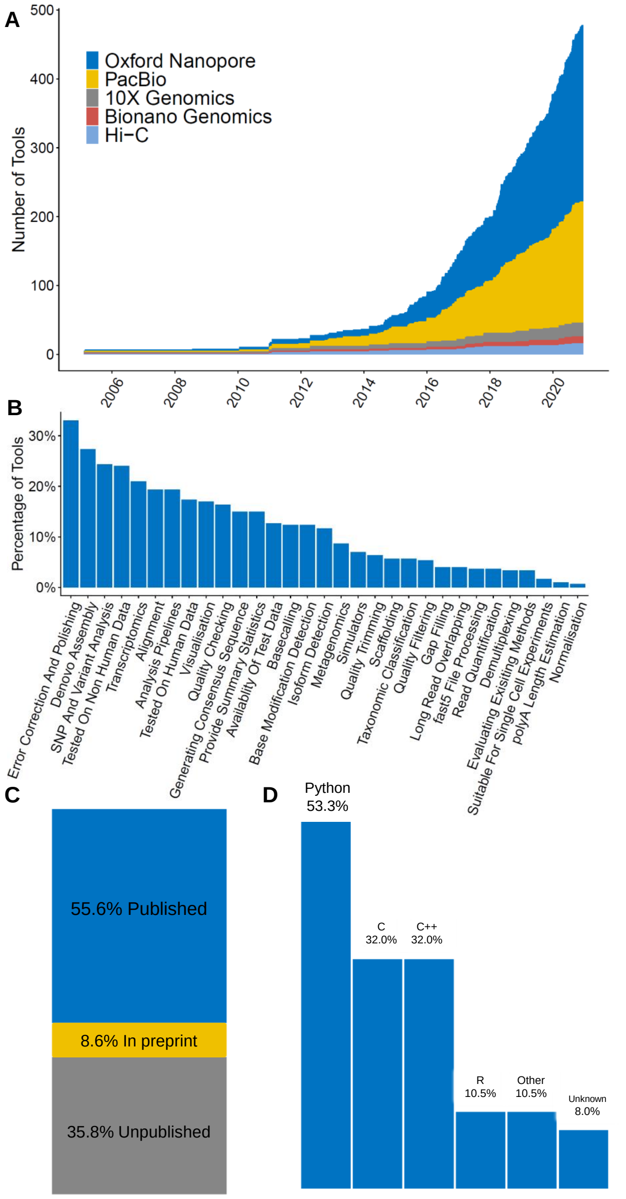

Figure 2. Summary statistics from long-read-tools.org A. The number of tools released over time stratified by the long-read technologies they serve. B. The data analysis categories covered by the catalogued tools (ordered from most to least frequent). C. Publication status of the catalogued tools. D. The programming platforms used by the catalogued tools (ordered from most to least frequent). All languages making up at least 10% of a tool's code are reported. These summary plots are available from the `Statistics` tab of the database website and can be easily exported for reuse.

can subset these findings further based on their preferred technology. Selecting Oxford Nanopore and PacBio returns the tools that are confirmed to work with both, thus removing *PRAPI* and *IsoSeq3* that are specialised for PacBio data (Figure 1D). However we note that a tool that has only been tested on one technology, and is thus annotated only with one, may well be applicable to another given the similarities in data characteristics between long-read platforms.

The `Statistics` tab contains summary plots obtained from an analysis of the information contained within the database (Figure 2, e.g. growth in tool development over time, the distribution of tools across analysis tasks, publication status, summary of the programming languages they use, etc.).

The `Submit` tab is where the user can provide new information to the database if they have a tool to submit or modify.

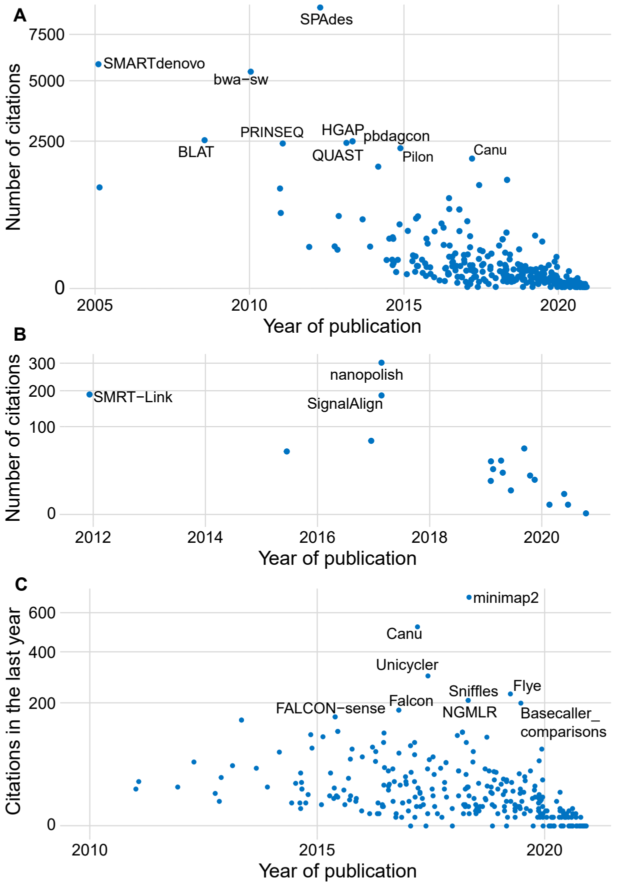

Figure 3. Popularity of the tools from long-read-tools.org based on publication citations A. Across the entire database. B. For *base modification detection*. C. Across the entire database for citations in the last year. Each panel shows the year of publication on the x-axis and the square root of the number of citations on the y-axis. If the input set of tools is larger than 50, the 10 most cited tools are labeled, otherwise the 3 most cited tools are labeled

The final tabs (`Updates`, `FAQs` and `Contact Us`) provide a summary of the social media activity of *@long_read_tools* (Twitter), answers to frequently asked questions and a form to contact the database creators to ask general questions, respectively.

Database Statistics

Long-read-tools.org contains 478 tools at the time of manuscript submission (Figure 2A). These include 229, 155, 20, 15 and 10 tools that can handle ONT, PacBio, 10x, Hi-C, Bionano Genomics data, respectively.

Tools began to appear in publications from the year 2005, although these were not targeted to long-read sequence analysis at that time. Tools focused on short-read alignment such as *Gmap* [14], *Soap-denovo* [15] and *STAR* [16] have made alterations to their algorithms in order to support error-prone long-read sequence alignments. Nevertheless, short-read aligners have also been tested for their ability to work with long reads [17].

Tools specifically focused on long-read sequence analysis became available from 2012, following the commercial release of the PacBio RS sequencer in 2011 (see for example PBcR [18, 19] and LSC [20]). The ONT MinION was commercially released in 2014, and Poretools was published in the same year [21].

Available tools are categorised into 32 different functions (Figure 2B). Of these, "error correction and polishing" and "*de novo* assembly" are the most common. On the other hand,

"polyA length estimation", "suitable for single cell experiments" and "normalisation" have the lowest number of tools, which highlights areas for further research and tool development.

It is also exciting to see the majority of the tools have been published in either a peer-reviewed journal or on a preprint server (Figure 2C). Moreover, tools written in Python outnumber tools implemented in other programming languages (Figure 2D).

In terms of the number of citations, *SPAdes* [22], *SMARTDenovo* [23] and *bwa-sw* [24] lead the pack (Figure 3A). However, it should be noted that these tools existed before long-read technologies were popular, and most of these citations will therefore not reflect their popularity in long-read data analysis. The number of citations provides a more accurate indicator of usage for the tools that are unique to long-read analyses (e.g. *nanopolish* [25], *SMRT-Link* [26] and *SignalAlign* [27] in "base modification detection" (Figure 3B). To better capture the popularity of tools in a rapidly-moving field, we also report the number of citations in the past year (Figure 3C). For instance it can be observed that the *Flye* assembler [28] has been highly cited in the past 12 months despite its recent publication date (April 2019).

Summary and Future Work

Long-read-tools.org is an an up-to-date, user-friendly catalogue that allows efficient searching of software by analysis category. It provides a comprehensive resource for new users to quickly and easily identify the relevant tools for their long-read data type and desired application. Our database illustrates the main areas of focus for existing tools, as well as the lack of software available in other areas (e.g. transcriptomics).

Other bioinformatic fields have experienced a similar growth in the number of available tools, prompting efforts to collate and organise them. These efforts vary from simple spreadsheets that list resources for the analysis of genomic repeats [29], through clickable lists of single-cell data analysis tools hosted on GitHub [30], all the way to dedicated websites offering search functions and statistics, such as scRNA-tools [31] that indexes tools for single-cell transcriptomics.

For long-read data, the long-read-catalog GitHub page [32] collects 40 tools for the analysis of ONT and PacBio data but it has not been updated in the last year. The Bioinformatics-Workflow-Frameworks-Platforms Google Sheets [33] list, among many other things, 84 tools relating to ONT data and 82 applicable to PacBio data. long-read-tools.org is both more comprehensive and easier to navigate than these databases.

We intend to keep increasing the breadth and depth of long-read-tools.org, but this should not come at the cost of making the database overwhelming to browse. Tutorials such as the 'Long-read, long reach Bioinformatics Tutorials' website [34] are helpful in understanding how multiple tools fit into an analysis pipeline. Therefore we are focusing current efforts on facilitating the identification of best practices, validated workflows, and each tool's relative strengths and weaknesses. Four additional entries are already available at tool submission and will be progressively populated: Underlying Algorithms, Underlying Assumptions, Strengths and Weaknesses, and Overall Performance. Furthermore a `Tutorials` tab highlighting common validated workflows and a `Benchmarks` tab featuring benchmarking studies and their results are in development.

Availability of Source Code and Requirements

- Project name: Long-read-tools.org database
- Project home page: long-read-tools.org
- Source code availability: https://github.com/shaniAmare/long_read_tools
- Operating system(s): Platform independent
- Programming language(s): R/JavaScript/html
- Other requirements: Accessible via any modern web browser
- License: MIT
- SciCrunch RRID:SCR_019116
- Biotools ID: biotools:long-read-tools

long-read-tools.org is a community effort, and we encourage researchers to contribute relevant tools, benchmarks, tutorials and improvements to the database via the `Submit` tab.

Declarations

List of Abbreviations

ONT : Oxford Nanopore Technologies
PacBio : Pacific Biosciences
Hi-C : An extension of chromosomal interactions using chromosome conformation capture (3C)
10x : 10x Genomics

Competing Interests

The authors declare that they have no competing interests.

Funding

This work was supported by funding from the Chan Zuckerberg Initiative DAF, an advised fund of Silicon Valley Community Foundation (grant number 2019-002443 to MER), a fellowship from the Australian National Health and Medical Research Council (NHMRC, grant number GNT1104924 to MER), Victorian State Government Operational Infrastructure Support and Australian Government NHMRC IRIISS.

Authors' Contributions

SLA structured the database, developed, implemented and populated it and wrote the manuscript. MER guided the research and wrote the manuscript. QG structured the database, populated and validated entries, and wrote the manuscript. All authors read and approved the final manuscript.

Acknowledgements

We thank Dr Luke Zappia, the main developer of the scRNA-tools.org database that this work builds upon, for his support in the initial stages of this project, Ms Xueyi Dong and Mr Shian Su for providing constructive feedback on the database, Mr Sujith S. Waduge, Mr Isuru Palliyaguru and Mr Jithendra Sirimanne for their guidance in making the JavaScript underlying the database visualisation more reproducible and user-friendly and Ms Tamara Beck and Ms Ellen Conti for creating the database logo.

References

1. Logsdon GA, Vollger MR, Eichler EE. Long-read human genome sequencing and its applications. Nature Re-

views Genetics 2020 jun;p. 1–18. https://doi.org/10.1038/s41576-020-0236-x.

2. Sakamoto Y, Sereewattanawoot S, Suzuki A. A new era of long-read sequencing for cancer genomics. Journal of Human Genetics 2020 jan;65(1):3–10. https://doi.org/10.1038/s10038-019-0658-5.
3. Ho SS, Urban AE, Mills RE. Structural variation in the sequencing era. Nature Reviews Genetics 2020 mar;21(3):171–189. https://doi.org/10.1038/s41576-019-0180-9.
4. Mitsuhashi S, Matsumoto N. Long-read sequencing for rare human genetic diseases. Journal of Human Genetics 2020 jan;65(1):11–19. https://doi.org/10.1038/s10038-019-0671-8.
5. Pollard MO, Gurdasani D, Mentzer AJ, Porter T, Sandhu MS. Long reads: their purpose and place. Human molecular genetics 2018 aug;27(R2):R234–R241. https://doi.org/10.1093/hmg/ddy177.
6. R Development Core Team. R: A Language and Environment for Statistical Computing. Vienna, Austria: R Foundation for Statistical Computing; 2012, http://www.R-project.org, ISBN 3-900051-07-0.
7. Chamberlain S, Zhu H, Jahn N, Boettiger C, Ram K. rcrossref: Client for Various 'CrossRef' 'APIs'; 2020, https://CRAN.R-project.org/package=rcrossref, r package version 1.0.0.
8. Chamberlain S. citecorp: Client for the Open Citations Corpus; 2020, https://CRAN.R-project.org/package=citecorp, r package version 0.3.0.
9. Heibi I, Peroni S, Shotton D. Software review: COCI, the OpenCitations Index of Crossref open DOI-to-DOI citations. Scientometrics 2019 Nov;121(2):1213–1228. http://link.springer.com/10.1007/s11192-019-03217-6.
10. Ram K, Broman K. aRxiv: Interface to the arXiv API; 2019, https://CRAN.R-project.org/package=aRxiv, r package version 0.5.19.
11. Wickham H. ggplot2: Elegant Graphics for Data Analysis. Springer-Verlag New York; 2016. https://ggplot2.tidyverse.org.
12. Sievert C. Interactive Web-Based Data Visualization with R, plotly, and shiny. Chapman and Hall/CRC; 2020. https://plotly-r.com.
13. Tardaguila M, de la Fuente L, Marti C, Pereira C, Pardo-Palacios FJ, del Risco H, et al. SQANTI: extensive characterization of long-read transcript sequences for quality control in full-length transcriptome identification and quantification. Genome Research 2018 Mar;28(3):396–411. http://genome.cshlp.org/lookup/doi/10.1101/gr.222976.117.
14. Wu TD, Watanabe CK. GMAP: a genomic mapping and alignment program for mRNA and EST sequences. Bioinformatics 2005 may;21(9):1859–1875. https://doi.org/10.1093/bioinformatics/bti310.
15. Luo R, Liu B, Xie Y, Li Z, Huang W, Yuan J, et al. SOAPdenovo2: an empirically improved memory-efficient short-read de novo assembler. GigaScience 2012 dec;1(1):18. https://doi.org/10.1186/2047-217X-1-18.
16. Dobin A, Davis CA, Schlesinger F, Drenkow J, Zaleski C, Jha S, et al. STAR: ultrafast universal RNA-seq aligner. Bioinformatics 2013 jan;29(1):15–21. https://doi.org/10.1093/bioinformatics/bts635.
17. Krizanovic K, Echchiki A, Roux J, Sikic M. Evaluation of tools for long read RNA-seq splice-aware alignment. bioRxiv 2017 apr;p. 126656. https://doi.org/10.1101/126656.
18. Koren S, Schatz MC, Walenz BP, Martin J, Howard JT, Ganapathy G, et al. Hybrid error correction and de novo assembly of single-molecule sequencing reads. Nature Biotechnology 2012 jul;30(7):693–700. https://www.nature.com/articles/nbt.2280.
19. Berlin K, Koren S, Chin CS, Drake JP, Landolin JM, Phillippy AM. Assembling large genomes with single-molecule sequencing and locality-sensitive hashing. Nature Biotechnology 2015 jun;33(6):623–630. https://www.nature.com/articles/nbt.3238.
20. Au KF, Underwood JG, Lee L, Wong WH. Improving PacBio Long Read Accuracy by Short Read Alignment. PLoS ONE 2012 oct;7(10). https://doi.org/10.1371/journal.pone.0046679.
21. Loman NJ, Quinlan AR. Poretools: a toolkit for analyzing nanopore sequence data. Bioinformatics 2014 08;30(23):3399–3401. https://doi.org/10.1093/bioinformatics/btu555.
22. Bankevich A, Nurk S, Antipov D, Gurevich AA, Dvorkin M, Kulikov AS, et al. SPAdes: A New Genome Assembly Algorithm and Its Applications to Single-Cell Sequencing. Journal of Computational Biology 2012 may;19(5):455–477. https://doi.org/10.1089/cmb.2012.0021.
23. Jue R, SMARTdenovo: Ultra-fast de novo assembler using long noisy reads;. https://github.com/ruanjue/smartdenovo, accessed on 26/08/2020.
24. Li H, Durbin R. Fast and accurate long-read alignment with Burrows-Wheeler transform. Bioinformatics 2010 jan;26(5):589–595. https://doi.org/10.1093/bioinformatics/btp698.
25. Loman NJ, Quick J, Simpson JT. A complete bacterial genome assembled de novo using only nanopore sequencing data. Nature Methods 2015 jul;12(8):733–735. https://doi.org/10.1038/nmeth.3444.
26. Ardui S, Ameur A, Vermeesch JR, Hestand MS. Single molecule real-time (SMRT) sequencing comes of age: Applications and utilities for medical diagnostics. Nucleic Acids Research 2018;46(5):2159–2168. https://doi.org/10.1093/nar/gky066.
27. Rand AC, Jain M, Eizenga JM, Musselman-Brown A, Olsen HE, Akeson M, et al. Mapping DNA methylation with high-throughput nanopore sequencing. Nature Methods 2017 feb;14(4):411–413. https://doi.org/10.1038/nmeth.4189.
28. Kolmogorov M, Yuan J, Lin Y, Pevzner PA. Assembly of long, error-prone reads using repeat graphs. Nature Biotechnology 2019 May;37(5):540–546. http://www.nature.com/articles/s41587-019-0072-8.
29. Repeat_Resources - Google Sheets;. https://docs.google.com/spreadsheets/d/1UBK70zExiL0gFVaIAILiGhflCGXAq_SF_lymaxTE1pY/edit#gid=1266138738, accessed on 26/08/2020.
30. Davis S, seandavi/awesome-single-cell: Community-curated list of software packages and data resources for single-cell, including RNA-seq, ATAC-seq, etc.;. https://github.com/seandavi/awesome-single-cell, accessed on 26/08/2020.
31. Zappia L, Phipson B, Oshlack A. Exploring the single-cell RNA-seq analysis landscape with the scRNA-tools database. PLOS Computational Biology 2018 jun;14(6):e1006245. https://doi.org/10.1371/journal.pcbi.1006245.
32. Molecular Microbiology and Infection Unit, University of Lisbon, B-UMMI/long-read-catalog: catalog for long-read sequencing tools;. https://github.com/B-UMMI/long-read-catalog, accessed on 26/08/2020.
33. Vilella A, Bioinformatics-Workflow-Frameworks-Platforms.v6.6.6 - Google Sheets;. https://docs.google.com/spreadsheets/d/1plkAsT_S3CzSeb7ivxyjRnHyrK3JclUCXeUMf_azraY/edit#gid=471877065, accessed on 26/08/2020.
34. Kahlke T, Long-read, long read bioinformatics tutorials;. https://timkahlke.github.io/LongRead_tutorials/, accessed on 26/08/2020.
