# Supplementary material for: long-read-tools.org: an interactive catalogue of analysis methods for long-read sequencing data
Source: Gigascience. 2021 Feb 16;10(2):giab003. doi: 10.1093/gigascience/giab003 (PMC7931822; doi:10.1093/gigascience/giab003)
Supplement: giab003_GIGA-D-20-00269_Revision_3 [file giab003_giga-d-20-00269_revision_3.pdf]

# GigaScience

## long-read-tools.org: an interactive catalogue of analysis methods for long-read sequencing data

--Manuscript Draft--

| | |
|---|---|
| **Manuscript Number:** | GIGA-D-20-00269R3 |
| **Full Title:** | long-read-tools.org: an interactive catalogue of analysis methods for long-read sequencing data |
| **Article Type:** | Technical Note |
| **Funding Information:** | Chan Zuckerberg Initiative — Dr Matthew E Ritchie<br>Silicon Valley Community Foundation (2019-002443) — Dr Matthew E Ritchie<br>National Health and Medical Research Council (GNT1104924) — Dr Matthew E Ritchie<br>State Government of Victoria — Dr Matthew E Ritchie<br>National Health and Medical Research Council (IRIISS) — Dr Matthew E Ritchie |
| **Abstract:** | Background<br><br>The data produced by long-read third-generation sequencers have unique characteristics compared to short-read sequencing data, often requiring tailored analysis tools for tasks ranging from quality control to downstream processing. The rapid growth in software that address these challenges for different genomics applications are difficult to keep track of, which makes it hard for users to choose the most appropriate tool for their analysis goal, and for developers to identify areas of need and existing solutions to benchmark against.<br>Findings<br><br>We describe the implementation of long-read-tools.org, an open-source database that organises the rapidly expanding collection of long-read data analysis tools and allows its exploration through interactive browsing and filtering. The current database release contains 478 tools across 32 categories. Most tools are developed in Python and the most frequent analysis tasks include basecalling, de novo assembly, error-correction, quality checking/filtering, and isoform detection, while long-read single-cell data analysis and transcriptomics are areas with the fewest tools available.<br>Conclusion<br><br>Continued growth in the application of long-read sequencing in genomics research positions the long-read-tools.org database as an essential resource that allows researchers to keep abreast of both established and emerging software to help guide the selection of the most relevant tool for their analysis needs. |
| **Corresponding Author:** | Quentin Gouil<br>Walter and Eliza Hall Institute of Medical Research<br>Parkville, VIC AUSTRALIA |
| **Corresponding Author Secondary Information:** | |
| **Corresponding Author's Institution:** | Walter and Eliza Hall Institute of Medical Research |
| **Corresponding Author's Secondary Institution:** | |
| **First Author:** | Shanika L Amarasinghe |
| **First Author Secondary Information:** | |
| **Order of Authors:** | Shanika L Amarasinghe |
| | |

Powered by Editorial Manager® and ProduXion Manager® from Aries Systems Corporation

| | |
|---|---|
| | Matthew E Ritchie |
| | Quentin Gouil |
| **Order of Authors Secondary Information:** | |
| **Response to Reviewers:** | Dear Dr Zauner,<br><br>1) I'm often mystified by the Editorial Manager's LaTeX behaviour! It looks like the pdf compiled without errors but the log is shown rather than the output pdf. I have tried with a different filename, changing the type from main LaTeX file to Manuscript, uploading the full zipped folder,... I couldn't work it out. I have been using the Gigascience template so hopefully it will be straightforward for the production team. It should just be a bug in EM and not with the LaTeX document.<br>2) Ref 34 now shows the doi.<br>Apologies for the back and forth. All the best,<br>Quentin |
| **Additional Information:** | |
| **Question** | **Response** |
| Are you submitting this manuscript to a special series or article collection? | No |
| **Experimental design and statistics**<br><br>Full details of the experimental design and statistical methods used should be given in the Methods section, as detailed in our Minimum Standards Reporting Checklist. Information essential to interpreting the data presented should be made available in the figure legends.<br><br>Have you included all the information requested in your manuscript? | No |
| If not, please give reasons for any omissions below.<br><br>as follow-up to "**Experimental design and statistics**<br><br>Full details of the experimental design and statistical methods used should be given in the Methods section, as detailed in our Minimum Standards Reporting Checklist. Information essential to interpreting the data presented should be made available in the figure legends.<br><br>Have you included all the information | no statistics applicable |

| | |
|---|---|
| requested in your manuscript?<br><br>" | |
| **Resources**<br><br>A description of all resources used, including antibodies, cell lines, animals and software tools, with enough information to allow them to be uniquely identified, should be included in the Methods section. Authors are strongly encouraged to cite Research Resource Identifiers (RRIDs) for antibodies, model organisms and tools, where possible.<br><br>Have you included the information requested as detailed in our Minimum Standards Reporting Checklist? | No |
| If not, please give reasons for any omissions below.<br><br>as follow-up to "**Resources**<br><br>A description of all resources used, including antibodies, cell lines, animals and software tools, with enough information to allow them to be uniquely identified, should be included in the Methods section. Authors are strongly encouraged to cite Research Resource Identifiers (RRIDs) for antibodies, model organisms and tools, where possible.<br><br>Have you included the information requested as detailed in our Minimum Standards Reporting Checklist?<br><br>" | not applicable |
| **Availability of data and materials**<br><br>All datasets and code on which the conclusions of the paper rely must be either included in your submission or deposited in publicly available repositories | Yes |

| | |
|---|---|
| (where available and ethically appropriate), referencing such data using a unique identifier in the references and in the “Availability of Data and Materials” section of your manuscript.<br><br>Have you have met the above requirement as detailed in our Minimum Standards Reporting Checklist? | |

LaTeX manuscript with bbl
Click here to access/download;LaTeX - Main Document (TeX file);main.tex

```
This is pdfTeX, Version 3.14159265-2.6-1.40.21 (TeX Live 2020/W32TeX)
(preloaded format=pdflatex 2020.5.12)  12 JAN 2021 18:13
entering extended mode
 restricted \write18 enabled.
 %&-line parsing enabled.
**main.tex
(./main.tex
LaTeX2e <2020-02-02> patch level 5
L3 programming layer <2020-05-05> (./oup-contemporary.cls
Document Class: oup-contemporary 2017/06/28, v1.1
(c:/TeXLive/2020/texmf-dist/tex/latex/base/article.cls
Document Class: article 2019/12/20 v1.4l Standard LaTeX document class
(c:/TeXLive/2020/texmf-dist/tex/latex/base/size10.clo
File: size10.clo 2019/12/20 v1.4l Standard LaTeX file (size option)
)
\c@part=\count167
\c@section=\count168
\c@subsection=\count169
\c@subsubsection=\count170
\c@paragraph=\count171
\c@subparagraph=\count172
\c@figure=\count173
\c@table=\count174
\abovecaptionskip=\skip47
\belowcaptionskip=\skip48
\bibindent=\dimen134
) (c:/TeXLive/2020/texmf-dist/tex/latex/base/inputenc.sty
Package: inputenc 2018/08/11 v1.3c Input encoding file
\inpenc@prehook=\toks15
\inpenc@posthook=\toks16
) (c:/TeXLive/2020/texmf-dist/tex/latex/base/fontenc.sty
Package: fontenc 2020/02/11 v2.0o Standard LaTeX package
) (c:/TeXLive/2020/texmf-dist/tex/generic/iftex/ifpdf.sty
Package: ifpdf 2019/10/25 v3.4 ifpdf legacy package. Use iftex instead.
(c:/TeXLive/2020/texmf-dist/tex/generic/iftex/iftex.sty
Package: iftex 2020/03/06 v1.0d TeX engine tests
)) (c:/TeXLive/2020/texmf-dist/tex/latex/microtype/microtype.sty
Package: microtype 2019/11/18 v2.7d Micro-typographical refinements (RS)
(c:/TeXLive/2020/texmf-dist/tex/latex/graphics/keyval.sty
Package: keyval 2014/10/28 v1.15 key=value parser (DPC)
\KV@toks@=\toks17
)
\MT@toks=\toks18
\MT@count=\count175
LaTeX Info: Redefining \textls on input line 790.
\MT@outer@kern=\dimen135
LaTeX Info: Redefining \textmicrotypecontext on input line 1354.
\MT@listname@count=\count176
(c:/TeXLive/2020/texmf-dist/tex/latex/microtype/microtype-pdftex.def
File: microtype-pdftex.def 2019/11/18 v2.7d Definitions specific to
pdftex (RS)

LaTeX Info: Redefining \lsstyle on input line 914.
LaTeX Info: Redefining \lslig on input line 914.
```

```
\MT@outer@space=\skip49
)
Package microtype Info: Loading configuration file microtype.cfg.
(c:/TeXLive/2020/texmf-dist/tex/latex/microtype/microtype.cfg
File: microtype.cfg 2019/11/18 v2.7d microtype main configuration file
(RS)
)) (c:/TeXLive/2020/texmf-dist/tex/latex/euler/euler.sty
Package: euler 1995/03/05 v2.5
Package: `euler' v2.5 <1995/03/05> (FJ and FMi)
LaTeX Font Info:    Redeclaring symbol font `letters' on input line 35.
LaTeX Font Info:    Encoding `OML' has changed to `U' for symbol font
(Font)              `letters' in the math version `normal' on input line
35.
LaTeX Font Info:    Overwriting symbol font `letters' in version `normal'
(Font)                  OML/cmm/m/it --> U/eur/m/n on input line 35.
LaTeX Font Info:    Encoding `OML' has changed to `U' for symbol font
(Font)              `letters' in the math version `bold' on input line
35.
LaTeX Font Info:    Overwriting symbol font `letters' in version `bold'
(Font)                  OML/cmm/b/it --> U/eur/m/n on input line 35.
LaTeX Font Info:    Overwriting symbol font `letters' in version `bold'
(Font)                  U/eur/m/n --> U/eur/b/n on input line 36.
LaTeX Font Info:    Redeclaring math symbol \Gamma on input line 47.
LaTeX Font Info:    Redeclaring math symbol \Delta on input line 48.
LaTeX Font Info:    Redeclaring math symbol \Theta on input line 49.
LaTeX Font Info:    Redeclaring math symbol \Lambda on input line 50.
LaTeX Font Info:    Redeclaring math symbol \Xi on input line 51.
LaTeX Font Info:    Redeclaring math symbol \Pi on input line 52.
LaTeX Font Info:    Redeclaring math symbol \Sigma on input line 53.
LaTeX Font Info:    Redeclaring math symbol \Upsilon on input line 54.
LaTeX Font Info:    Redeclaring math symbol \Phi on input line 55.
LaTeX Font Info:    Redeclaring math symbol \Psi on input line 56.
LaTeX Font Info:    Redeclaring math symbol \Omega on input line 57.
\symEulerFraktur=\mathgroup4
LaTeX Font Info:    Overwriting symbol font `EulerFraktur' in version
`bold'
(Font)                  U/euf/m/n --> U/euf/b/n on input line 63.
LaTeX Info: Redefining \oldstylenums on input line 85.
\symEulerScript=\mathgroup5
LaTeX Font Info:    Overwriting symbol font `EulerScript' in version
`bold'
(Font)                  U/eus/m/n --> U/eus/b/n on input line 93.
LaTeX Font Info:    Redeclaring math symbol \aleph on input line 97.
LaTeX Font Info:    Redeclaring math symbol \Re on input line 98.
LaTeX Font Info:    Redeclaring math symbol \Im on input line 99.
LaTeX Font Info:    Redeclaring math delimiter \vert on input line 101.
LaTeX Font Info:    Redeclaring math delimiter \backslash on input line
103.
LaTeX Font Info:    Redeclaring math symbol \neg on input line 106.
LaTeX Font Info:    Redeclaring math symbol \wedge on input line 108.
LaTeX Font Info:    Redeclaring math symbol \vee on input line 110.
LaTeX Font Info:    Redeclaring math symbol \setminus on input line 112.
LaTeX Font Info:    Redeclaring math symbol \sim on input line 113.
LaTeX Font Info:    Redeclaring math symbol \mid on input line 114.
```

```
LaTeX Font Info:    Redeclaring math delimiter \arrowvert on input line
116.
LaTeX Font Info:    Redeclaring math symbol \mathsection on input line
117.
\symEulerExtension=\mathgroup6
LaTeX Font Info:    Redeclaring math symbol \coprod on input line 125.
LaTeX Font Info:    Redeclaring math symbol \prod on input line 125.
LaTeX Font Info:    Redeclaring math symbol \sum on input line 125.
LaTeX Font Info:    Redeclaring math symbol \intop on input line 130.
LaTeX Font Info:    Redeclaring math symbol \ointop on input line 131.
LaTeX Font Info:    Redeclaring math symbol \braceld on input line 132.
LaTeX Font Info:    Redeclaring math symbol \bracerd on input line 133.
LaTeX Font Info:    Redeclaring math symbol \bracelu on input line 134.
LaTeX Font Info:    Redeclaring math symbol \braceru on input line 135.
LaTeX Font Info:    Redeclaring math symbol \infty on input line 136.
LaTeX Font Info:    Redeclaring math symbol \nearrow on input line 153.
LaTeX Font Info:    Redeclaring math symbol \searrow on input line 154.
LaTeX Font Info:    Redeclaring math symbol \nwarrow on input line 155.
LaTeX Font Info:    Redeclaring math symbol \swarrow on input line 156.
LaTeX Font Info:    Redeclaring math symbol \Leftrightarrow on input line
157.
LaTeX Font Info:    Redeclaring math symbol \Leftarrow on input line 158.
LaTeX Font Info:    Redeclaring math symbol \Rightarrow on input line
159.
LaTeX Font Info:    Redeclaring math symbol \leftrightarrow on input line
160.
LaTeX Font Info:    Redeclaring math symbol \leftarrow on input line 161.
LaTeX Font Info:    Redeclaring math symbol \rightarrow on input line
163.
LaTeX Font Info:    Redeclaring math delimiter \uparrow on input line
166.
LaTeX Font Info:    Redeclaring math delimiter \downarrow on input line
168.
LaTeX Font Info:    Redeclaring math delimiter \updownarrow on input line
170.
LaTeX Font Info:    Redeclaring math delimiter \Uparrow on input line
172.
LaTeX Font Info:    Redeclaring math delimiter \Downarrow on input line
174.
LaTeX Font Info:    Redeclaring math delimiter \Updownarrow on input line
176.
LaTeX Font Info:    Redeclaring math symbol \leftharpoonup on input line
177.
LaTeX Font Info:    Redeclaring math symbol \leftharpoondown on input
line 178.

LaTeX Font Info:    Redeclaring math symbol \rightharpoonup on input line
179.
LaTeX Font Info:    Redeclaring math symbol \rightharpoondown on input
line 180
.
LaTeX Font Info:    Redeclaring math delimiter \lbrace on input line 182.
LaTeX Font Info:    Redeclaring math delimiter \rbrace on input line 184.
\symcmmigroup=\mathgroup7
```

```
LaTeX Font Info:    Overwriting symbol font `cmmigroup' in version `bold'
(Font)                  OML/cmm/m/it --> OML/cmm/b/it on input line 200.
LaTeX Font Info:    Redeclaring math accent \vec on input line 201.
LaTeX Font Info:    Redeclaring math symbol \triangleleft on input line
202.
LaTeX Font Info:    Redeclaring math symbol \triangleright on input line
203.
LaTeX Font Info:    Redeclaring math symbol \star on input line 204.
LaTeX Font Info:    Redeclaring math symbol \lhook on input line 205.
LaTeX Font Info:    Redeclaring math symbol \rhook on input line 206.
LaTeX Font Info:    Redeclaring math symbol \flat on input line 207.
LaTeX Font Info:    Redeclaring math symbol \natural on input line 208.
LaTeX Font Info:    Redeclaring math symbol \sharp on input line 209.
LaTeX Font Info:    Redeclaring math symbol \smile on input line 210.
LaTeX Font Info:    Redeclaring math symbol \frown on input line 211.
LaTeX Font Info:    Redeclaring math accent \grave on input line 245.
LaTeX Font Info:    Redeclaring math accent \acute on input line 246.
LaTeX Font Info:    Redeclaring math accent \tilde on input line 247.
LaTeX Font Info:    Redeclaring math accent \ddot on input line 248.
LaTeX Font Info:    Redeclaring math accent \check on input line 249.
LaTeX Font Info:    Redeclaring math accent \breve on input line 250.
LaTeX Font Info:    Redeclaring math accent \bar on input line 251.
LaTeX Font Info:    Redeclaring math accent \dot on input line 252.
LaTeX Font Info:    Redeclaring math accent \hat on input line 254.
) (c:/TeXLive/2020/texmf-dist/tex/latex/merriweather/merriweather.sty
Package: merriweather 2019/10/13 (Bob Tennent) Supports
Merriweather(Sans) font
s for all LaTeX engines.
(c:/TeXLive/2020/texmf-dist/tex/generic/iftex/ifxetex.sty
Package: ifxetex 2019/10/25 v0.7 ifxetex legacy package. Use iftex
instead.
) (c:/TeXLive/2020/texmf-dist/tex/generic/iftex/ifluatex.sty
Package: ifluatex 2019/10/25 v1.5 ifluatex legacy package. Use iftex
instead.
) (c:/TeXLive/2020/texmf-dist/tex/latex/base/textcomp.sty
Package: textcomp 2020/02/02 v2.0n Standard LaTeX package
) (c:/TeXLive/2020/texmf-dist/tex/latex/xkeyval/xkeyval.sty
Package: xkeyval 2014/12/03 v2.7a package option processing (HA)
(c:/TeXLive/2020/texmf-dist/tex/generic/xkeyval/xkeyval.tex
(c:/TeXLive/2020/te
xmf-dist/tex/generic/xkeyval/xkvutils.tex
\XKV@toks=\toks19
\XKV@tempa@toks=\toks20
)
\XKV@depth=\count177
File: xkeyval.tex 2014/12/03 v2.7a key=value parser (HA)
)) (c:/TeXLive/2020/texmf-dist/tex/latex/base/fontenc.sty
Package: fontenc 2020/02/11 v2.0o Standard LaTeX package
) (c:/TeXLive/2020/texmf-dist/tex/latex/fontaxes/fontaxes.sty
Package: fontaxes 2014/03/23 v1.0d Font selection axes
LaTeX Info: Redefining \upshape on input line 29.
LaTeX Info: Redefining \itshape on input line 31.
LaTeX Info: Redefining \slshape on input line 33.
LaTeX Info: Redefining \swshape on input line 35.
```

```
LaTeX Info: Redefining \scshape on input line 37.
LaTeX Info: Redefining \sscshape on input line 39.
LaTeX Info: Redefining \ulcshape on input line 41.
LaTeX Info: Redefining \textsw on input line 47.
LaTeX Info: Redefining \textssc on input line 48.
LaTeX Info: Redefining \textulc on input line 49.
)) (c:/TeXLive/2020/texmf-dist/tex/latex/mathastext/mathastext.sty
Package: mathastext 2019/11/16 v1.3w Use the text font in math mode (JFB)
\mst@exists@muskip=\muskip16
\mst@forall@muskip=\muskip17
\mst@prime@muskip=\muskip18
\mst@do@nonletters=\toks21
\mst@do@easynonletters=\toks22
\mst@do@az=\toks23
\mst@do@AZ=\toks24
\symmtoperatorfont=\mathgroup8
\symmtletterfont=\mathgroup9
** ! and ?
** punctuation: , . : ; and \colon
LaTeX Info: Redefining \relbar on input line 787.
LaTeX Info: Redefining \rightarrowfill on input line 790.
LaTeX Info: Redefining \leftarrowfill on input line 795.
** + and =
LaTeX Info: Redefining \Relbar on input line 886.
** adding = ; and + to \nfss@catcodes
** parentheses ( ) [ ] and slash /
** alldelims: < > \backslash \setminus | \vert \mid \{ and \}
LaTeX Font Info:    Redeclaring math delimiter \backslash on input line
932.
LaTeX Font Info:    Redeclaring math symbol \setminus on input line 944.
LaTeX Info: Redefining \models on input line 953.
** \# \mathdollar \% \&
** \imath and \jmath
LaTeX Font Info:    Overwriting math alphabet `\mathnormalbold' in
version `nor
mal'
(Font)                  T1/Merriweather-OsF/b/it --> T1/Merriweather-
OsF/b/it o
n input line 2140.
LaTeX Font Info:    Overwriting math alphabet `\mathnormalbold' in
version `bol
d'
(Font)                  T1/Merriweather-OsF/b/it --> T1/Merriweather-
OsF/b/it o
n input line 2140.
LaTeX Font Info:    Overwriting symbol font `mtletterfont' in version
`normal'
(Font)                  T1/Merriweather-OsF/m/it --> T1/Merriweather-
OsF/m/it o
n input line 2140.
LaTeX Font Info:    Overwriting symbol font `mtletterfont' in version
`bold'
(Font)                  T1/Merriweather-OsF/m/it --> T1/Merriweather-
OsF/b/it o
```

```
n input line 2140.
LaTeX Font Info:    Overwriting symbol font `mtoperatorfont' in version
`normal
'
(Font)                  T1/Merriweather-OsF/m/n --> T1/Merriweather-
OsF/m/n on
input line 2140.
LaTeX Font Info:    Overwriting symbol font `mtoperatorfont' in version
`bold'
(Font)                  T1/Merriweather-OsF/m/n --> T1/Merriweather-
OsF/b/n on
input line 2140.
LaTeX Font Info:    Overwriting math alphabet `\Mathbf' in version
`normal'
(Font)                  T1/Merriweather-OsF/b/n --> T1/Merriweather-
OsF/b/n on
input line 2140.
LaTeX Font Info:    Overwriting math alphabet `\Mathbf' in version `bold'
(Font)                  T1/Merriweather-OsF/b/n --> T1/Merriweather-
OsF/b/n on
input line 2140.
LaTeX Font Info:    Overwriting math alphabet `\Mathit' in version
`normal'
(Font)                  T1/Merriweather-OsF/m/it --> T1/Merriweather-
OsF/m/it o
n input line 2140.
LaTeX Font Info:    Overwriting math alphabet `\Mathit' in version `bold'
(Font)                  T1/Merriweather-OsF/m/it --> T1/Merriweather-
OsF/b/it o
n input line 2140.
LaTeX Font Info:    Overwriting math alphabet `\Mathsf' in version
`normal'
(Font)                  T1/MerriweatherSans-OsF/m/n -->
T1/MerriweatherSans-OsF
/m/n on input line 2140.
LaTeX Font Info:    Overwriting math alphabet `\Mathsf' in version `bold'
(Font)                  T1/MerriweatherSans-OsF/m/n -->
T1/MerriweatherSans-OsF
/b/n on input line 2140.
LaTeX Font Info:    Overwriting math alphabet `\Mathtt' in version
`normal'
(Font)                  T1/lmtt/m/n --> T1/lmtt/m/n on input line 2140.
LaTeX Font Info:    Overwriting math alphabet `\Mathtt' in version `bold'
(Font)                  T1/lmtt/m/n --> T1/lmtt/b/n on input line 2140.
** Latin letters in the normal (resp. bold) math versions are now
** set up to use the fonts T1/Merriweather-OsF/m(b)/it
** Other characters (digits, ...) and \log-like names will be
** typeset with the n shape.
** \hbar
** minus as endash
** \HUGE has been (re)-defined.
** mathastext has declared larger sizes for subscripts.
** To keep LaTeX defaults, use option `defaultmathsizes'.
) (c:/TeXLive/2020/texmf-dist/tex/latex/relsize/relsize.sty
```

```
Package: relsize 2013/03/29 ver 4.1
) (c:/TeXLive/2020/texmf-dist/tex/latex/ragged2e/ragged2e.sty
Package: ragged2e 2019/07/28 v2.2 ragged2e Package (MS)
(c:/TeXLive/2020/texmf-dist/tex/latex/ms/everysel.sty
Package: everysel 2011/10/28 v1.2 EverySelectfont Package (MS)
)
\CenteringLeftskip=\skip50
\RaggedLeftLeftskip=\skip51
\RaggedRightLeftskip=\skip52
\CenteringRightskip=\skip53
\RaggedLeftRightskip=\skip54
\RaggedRightRightskip=\skip55
\CenteringParfillskip=\skip56
\RaggedLeftParfillskip=\skip57
\RaggedRightParfillskip=\skip58
\JustifyingParfillskip=\skip59
\CenteringParindent=\skip60
\RaggedLeftParindent=\skip61
\RaggedRightParindent=\skip62
\JustifyingParindent=\skip63
) (c:/TeXLive/2020/texmf-dist/tex/latex/xcolor/xcolor.sty
Package: xcolor 2016/05/11 v2.12 LaTeX color extensions (UK)
(c:/TeXLive/2020/texmf-dist/tex/latex/graphics-cfg/color.cfg
File: color.cfg 2016/01/02 v1.6 sample color configuration
)
Package xcolor Info: Driver file: pdftex.def on input line 225.
(c:/TeXLive/2020/texmf-dist/tex/latex/graphics-def/pdftex.def
File: pdftex.def 2018/01/08 v1.0l Graphics/color driver for pdftex
)
Package xcolor Info: Model `cmy' substituted by `cmy0' on input line
1348.
Package xcolor Info: Model `hsb' substituted by `rgb' on input line 1352.
Package xcolor Info: Model `RGB' extended on input line 1364.
Package xcolor Info: Model `HTML' substituted by `rgb' on input line
1366.
Package xcolor Info: Model `Hsb' substituted by `hsb' on input line 1367.
Package xcolor Info: Model `tHsb' substituted by `hsb' on input line
1368.
Package xcolor Info: Model `HSB' substituted by `hsb' on input line 1369.
Package xcolor Info: Model `Gray' substituted by `gray' on input line
1370.
Package xcolor Info: Model `wave' substituted by `hsb' on input line
1371.
) (c:/TeXLive/2020/texmf-dist/tex/latex/colortbl/colortbl.sty
Package: colortbl 2020/01/04 v1.0e Color table columns (DPC)
(c:/TeXLive/2020/texmf-dist/tex/latex/tools/array.sty
Package: array 2019/08/31 v2.4l Tabular extension package (FMi)
\col@sep=\dimen136
\ar@mcellbox=\box45
\extrarowheight=\dimen137
\NC@list=\toks25
\extratabsurround=\skip64
\backup@length=\skip65
\ar@cellbox=\box46
```

```
)
\everycr=\toks26
\minrowclearance=\skip66
) (c:/TeXLive/2020/texmf-dist/tex/latex/graphics/graphicx.sty
Package: graphicx 2019/11/30 v1.2a Enhanced LaTeX Graphics (DPC,SPQR)
(c:/TeXLive/2020/texmf-dist/tex/latex/graphics/graphics.sty
Package: graphics 2019/11/30 v1.4a Standard LaTeX Graphics (DPC,SPQR)
(c:/TeXLive/2020/texmf-dist/tex/latex/graphics/trig.sty
Package: trig 2016/01/03 v1.10 sin cos tan (DPC)
) (c:/TeXLive/2020/texmf-dist/tex/latex/graphics-cfg/graphics.cfg
File: graphics.cfg 2016/06/04 v1.11 sample graphics configuration
)
Package graphics Info: Driver file: pdftex.def on input line 105.
)
\Gin@req@height=\dimen138
\Gin@req@width=\dimen139
) (c:/TeXLive/2020/texmf-dist/tex/latex/etoolbox/etoolbox.sty
Package: etoolbox 2019/09/21 v2.5h e-TeX tools for LaTeX (JAW)
\etb@tempcnta=\count178
) (c:/TeXLive/2020/texmf-dist/tex/latex/xpatch/xpatch.sty
(c:/TeXLive/2020/texm
f-dist/tex/latex/l3kernel/expl3.sty
Package: expl3 2020-05-05 L3 programming layer (loader)
(c:/TeXLive/2020/texmf-dist/tex/latex/l3backend/l3backend-pdfmode.def
File: l3backend-pdfmode.def 2020-05-05 L3 backend support: PDF mode
\l__kernel_color_stack_int=\count179
\l__pdf_internal_box=\box47
))
Package: xpatch 2020/03/25 v0.3a Extending etoolbox patching commands
(c:/TeXLive/2020/texmf-dist/tex/latex/l3packages/xparse/xparse.sty
Package: xparse 2020-03-06 L3 Experimental document command parser
\l__xparse_current_arg_int=\count180
\g__xparse_grabber_int=\count181
\l__xparse_m_args_int=\count182
\l__xparse_v_nesting_int=\count183
)) (c:/TeXLive/2020/texmf-dist/tex/latex/environ/environ.sty
Package: environ 2014/05/04 v0.3 A new way to define environments
(c:/TeXLive/2020/texmf-dist/tex/latex/trimspaces/trimspaces.sty
Package: trimspaces 2009/09/17 v1.1 Trim spaces around a token list
)
\@envbody=\toks27
) (c:/TeXLive/2020/texmf-dist/tex/latex/lastpage/lastpage.sty
Package: lastpage 2015/03/29 v1.2m Refers to last page's name (HMM; JPG)
) (c:/TeXLive/2020/texmf-dist/tex/latex/graphics/rotating.sty
Package: rotating 2016/08/11 v2.16d rotated objects in LaTeX
(c:/TeXLive/2020/texmf-dist/tex/latex/base/ifthen.sty
Package: ifthen 2014/09/29 v1.1c Standard LaTeX ifthen package (DPC)
)
\c@r@tfl@t=\count184
\rotFPtop=\skip67
\rotFPbot=\skip68
\rot@float@box=\box48
\rot@mess@toks=\toks28
) (c:/TeXLive/2020/texmf-dist/tex/latex/graphics/lscape.sty
```

```
Package: lscape 2000/10/22 v3.01 Landscape Pages (DPC)
) (c:/TeXLive/2020/texmf-dist/tex/latex/tools/afterpage.sty
Package: afterpage 2014/10/28 v1.08 After-Page Package (DPC)
\AP@output=\toks29
\AP@partial=\box49
\AP@footins=\box50
) (c:/TeXLive/2020/texmf-dist/tex/latex/textpos/textpos.sty
Package: textpos 2019/04/15 v1.9.1
Package: textpos 2019/04/15 1.9.1, absolute positioning of text on the
page
(c:/TeXLive/2020/texmf-dist/tex/latex/ms/everyshi.sty
Package: everyshi 2001/05/15 v3.00 EveryShipout Package (MS)
)
\TP@textbox=\box51
\TP@holdbox=\box52
\TPHorizModule=\dimen140
\TPVertModule=\dimen141
\TP@margin=\dimen142
\TP@absmargin=\dimen143
Grid set 16 x 16 = 37.34424pt x 52.81541pt
\TPboxrulesize=\dimen144
\TP@ox=\dimen145
\TP@oy=\dimen146
\TP@tbargs=\toks30
\TP@prevdepth=\dimen147
TextBlockOrigin set to 0pt x 0pt
) (c:/TeXLive/2020/texmf-dist/tex/latex/url/url.sty
\Urlmuskip=\muskip19
Package: url 2013/09/16  ver 3.4  Verb mode for urls, etc.
) (c:/TeXLive/2020/texmf-dist/tex/latex/newfloat/newfloat.sty
Package: newfloat 2019/09/02 v1.1l Defining new floating environments
(AR)
Package newfloat Info: `rotating' package detected.
) (c:/TeXLive/2020/texmf-dist/tex/latex/mdframed/mdframed.sty
Package: mdframed 2013/07/01 1.9b: mdframed
(c:/TeXLive/2020/texmf-dist/tex/latex/kvoptions/kvoptions.sty
Package: kvoptions 2019/11/29 v3.13 Key value format for package options
(HO)
(c:/TeXLive/2020/texmf-dist/tex/generic/ltxcmds/ltxcmds.sty
Package: ltxcmds 2019/12/15 v1.24 LaTeX kernel commands for general use
(HO)
) (c:/TeXLive/2020/texmf-dist/tex/generic/kvsetkeys/kvsetkeys.sty
Package: kvsetkeys 2019/12/15 v1.18 Key value parser (HO)
)) (c:/TeXLive/2020/texmf-dist/tex/latex/zref/zref-abspage.sty
Package: zref-abspage 2020-03-03 v2.29 Module abspage for zref (HO)
(c:/TeXLive/2020/texmf-dist/tex/latex/zref/zref-base.sty
Package: zref-base 2020-03-03 v2.29 Module base for zref (HO)
(c:/TeXLive/2020/texmf-dist/tex/generic/infwarerr/infwarerr.sty
Package: infwarerr 2019/12/03 v1.5 Providing info/warning/error messages
(HO)
) (c:/TeXLive/2020/texmf-dist/tex/generic/kvdefinekeys/kvdefinekeys.sty
Package: kvdefinekeys 2019-12-19 v1.6 Define keys (HO)
) (c:/TeXLive/2020/texmf-dist/tex/latex/pdftexcmds/pdftexcmds.sty
```

```
Package: pdftexcmds 2019/11/24 v0.31 Utility functions of pdfTeX for
LuaTeX (HO
)
Package pdftexcmds Info: \pdf@primitive is available.
Package pdftexcmds Info: \pdf@ifprimitive is available.
Package pdftexcmds Info: \pdfdraftmode found.
) (c:/TeXLive/2020/texmf-dist/tex/generic/etexcmds/etexcmds.sty
Package: etexcmds 2019/12/15 v1.7 Avoid name clashes with e-TeX commands
(HO)
) (c:/TeXLive/2020/texmf-dist/tex/latex/auxhook/auxhook.sty
Package: auxhook 2019-12-17 v1.6 Hooks for auxiliary files (HO)
)
Package zref Info: New property list: main on input line 763.
Package zref Info: New property: default on input line 764.
Package zref Info: New property: page on input line 765.
) (c:/TeXLive/2020/texmf-dist/tex/generic/atbegshi/atbegshi.sty
Package: atbegshi 2019/12/05 v1.19 At begin shipout hook (HO)
)
\c@abspage=\count185
Package zref Info: New property: abspage on input line 66.
) (c:/TeXLive/2020/texmf-dist/tex/latex/needspace/needspace.sty
Package: needspace 2010/09/12 v1.3d reserve vertical space
)
\mdf@templength=\skip69
\c@mdf@globalstyle@cnt=\count186
\mdf@skipabove@length=\skip70
\mdf@skipbelow@length=\skip71
\mdf@leftmargin@length=\skip72
\mdf@rightmargin@length=\skip73
\mdf@innerleftmargin@length=\skip74
\mdf@innerrightmargin@length=\skip75
\mdf@innertopmargin@length=\skip76
\mdf@innerbottommargin@length=\skip77
\mdf@splittopskip@length=\skip78
\mdf@splitbottomskip@length=\skip79
\mdf@outermargin@length=\skip80
\mdf@innermargin@length=\skip81
\mdf@linewidth@length=\skip82
\mdf@innerlinewidth@length=\skip83
\mdf@middlelinewidth@length=\skip84
\mdf@outerlinewidth@length=\skip85
\mdf@roundcorner@length=\skip86
\mdf@footenotedistance@length=\skip87
\mdf@userdefinedwidth@length=\skip88
\mdf@needspace@length=\skip89
\mdf@frametitleaboveskip@length=\skip90
\mdf@frametitlebelowskip@length=\skip91
\mdf@frametitlerulewidth@length=\skip92
\mdf@frametitleleftmargin@length=\skip93
\mdf@frametitlerightmargin@length=\skip94
\mdf@shadowsize@length=\skip95
\mdf@extratopheight@length=\skip96
\mdf@subtitleabovelinewidth@length=\skip97
\mdf@subtitlebelowlinewidth@length=\skip98
```

```
\mdf@subtitleaboveskip@length=\skip99
\mdf@subtitlebelowskip@length=\skip100
\mdf@subtitleinneraboveskip@length=\skip101
\mdf@subtitleinnerbelowskip@length=\skip102
\mdf@subsubtitleabovelinewidth@length=\skip103
\mdf@subsubtitlebelowlinewidth@length=\skip104
\mdf@subsubtitleaboveskip@length=\skip105
\mdf@subsubtitlebelowskip@length=\skip106
\mdf@subsubtitleinneraboveskip@length=\skip107
\mdf@subsubtitleinnerbelowskip@length=\skip108
(c:/TeXLive/2020/texmf-dist/tex/latex/mdframed/md-frame-0.mdf
File: md-frame-0.mdf 2013/07/01\ 1.9b: md-frame-0
)
\mdf@frametitlebox=\box53
\mdf@footnotebox=\box54
\mdf@splitbox@one=\box55
\mdf@splitbox@two=\box56
\mdf@splitbox@save=\box57
\mdfsplitboxwidth=\skip109
\mdfsplitboxtotalwidth=\skip110
\mdfsplitboxheight=\skip111
\mdfsplitboxdepth=\skip112
\mdfsplitboxtotalheight=\skip113
\mdfframetitleboxwidth=\skip114
\mdfframetitleboxtotalwidth=\skip115
\mdfframetitleboxheight=\skip116
\mdfframetitleboxdepth=\skip117
\mdfframetitleboxtotalheight=\skip118
\mdffootnoteboxwidth=\skip119
\mdffootnoteboxtotalwidth=\skip120
\mdffootnoteboxheight=\skip121
\mdffootnoteboxdepth=\skip122
\mdffootnoteboxtotalheight=\skip123
\mdftotallinewidth=\skip124
\mdfboundingboxwidth=\skip125
\mdfboundingboxtotalwidth=\skip126
\mdfboundingboxheight=\skip127
\mdfboundingboxdepth=\skip128
\mdfboundingboxtotalheight=\skip129
\mdf@freevspace@length=\skip130
\mdf@horizontalwidthofbox@length=\skip131
\mdf@verticalmarginwhole@length=\skip132
\mdf@horizontalspaceofbox=\skip133
\mdfsubtitleheight=\skip134
\mdfsubsubtitleheight=\skip135
\c@mdfcountframes=\count187

****** mdframed patching \endmdf@trivlist

****** -- success******

\mdf@envdepth=\count188
\c@mdf@env@i=\count189
\c@mdf@env@ii=\count190
```

```
\c@mdf@zref@counter=\count191
Package zref Info: New property: mdf@pagevalue on input line 895.
) (c:/TeXLive/2020/texmf-dist/tex/latex/titlesec/titlesec.sty
Package: titlesec 2019/10/16 v2.13 Sectioning titles
\ttl@box=\box58
\beforetitleunit=\skip136
\aftertitleunit=\skip137
\ttl@plus=\dimen148
\ttl@minus=\dimen149
\ttl@toksa=\toks31
\titlewidth=\dimen150
\titlewidthlast=\dimen151
\titlewidthfirst=\dimen152
) (c:/TeXLive/2020/texmf-dist/tex/latex/koma-script/scrextend.sty
Package: scrextend 2020/04/19 v3.30 KOMA-Script package (extend other
classes w
ith features of KOMA-Script classes)
(c:/TeXLive/2020/texmf-dist/tex/latex/koma-script/scrkbase.sty
Package: scrkbase 2020/04/19 v3.30 KOMA-Script package (KOMA-Script-
dependent b
asics and keyval usage)
(c:/TeXLive/2020/texmf-dist/tex/latex/koma-script/scrbase.sty
Package: scrbase 2020/04/19 v3.30 KOMA-Script package (KOMA-Script-
independent
basics and keyval usage)
(c:/TeXLive/2020/texmf-dist/tex/latex/koma-script/scrlfile.sty
Package: scrlfile 2020/04/19 v3.30 KOMA-Script package (loading files)
)))
Package scrextend Info: unexpected definition of `\@makefnmark'.
(scrextend)             Trying to patch it on input line 1589.
Package scrextend Info: patch seems to be successfull on input line 1589.
)

LaTeX Font Warning: Font shape `T1/cmr/m/n' in size <7.5> not available
(Font)              size <7> substituted on input line 65.

(c:/TeXLive/2020/texmf-dist/tex/latex/tools/calc.sty
Package: calc 2017/05/25 v4.3 Infix arithmetic (KKT,FJ)
\calc@Acount=\count192
\calc@Bcount=\count193
\calc@Adimen=\dimen153
\calc@Bdimen=\dimen154
\calc@Askip=\skip138
\calc@Bskip=\skip139
LaTeX Info: Redefining \setlength on input line 80.
LaTeX Info: Redefining \addtolength on input line 81.
\calc@Ccount=\count194
\calc@Cskip=\skip140
) (c:/TeXLive/2020/texmf-dist/tex/latex/geometry/geometry.sty
Package: geometry 2020/01/02 v5.9 Page Geometry
(c:/TeXLive/2020/texmf-dist/tex/generic/iftex/ifvtex.sty
Package: ifvtex 2019/10/25 v1.7 ifvtex legacy package. Use iftex instead.
)
\Gm@cnth=\count195
```

```
\Gm@cntv=\count196
\c@Gm@tempcnt=\count197
\Gm@bindingoffset=\dimen155
\Gm@wd@mp=\dimen156
\Gm@odd@mp=\dimen157
\Gm@even@mp=\dimen158
\Gm@layoutwidth=\dimen159
\Gm@layoutheight=\dimen160
\Gm@layouthoffset=\dimen161
\Gm@layoutvoffset=\dimen162
\Gm@dimlist=\toks32
) (c:/TeXLive/2020/texmf-dist/tex/latex/hyperref/hyperref.sty
Package: hyperref 2020/01/14 v7.00d Hypertext links for LaTeX
(c:/TeXLive/2020/texmf-dist/tex/generic/pdfescape/pdfescape.sty
Package: pdfescape 2019/12/09 v1.15 Implements pdfTeX's escape features
(HO)
) (c:/TeXLive/2020/texmf-dist/tex/latex/hycolor/hycolor.sty
Package: hycolor 2020-01-27 v1.10 Color options for hyperref/bookmark
(HO)
) (c:/TeXLive/2020/texmf-dist/tex/latex/letltxmacro/letltxmacro.sty
Package: letltxmacro 2019/12/03 v1.6 Let assignment for LaTeX macros (HO)
)
\@linkdim=\dimen163
\Hy@linkcounter=\count198
\Hy@pagecounter=\count199
(c:/TeXLive/2020/texmf-dist/tex/latex/hyperref/pd1enc.def
File: pd1enc.def 2020/01/14 v7.00d Hyperref: PDFDocEncoding definition
(HO)
Now handling font encoding PD1 ...
... no UTF-8 mapping file for font encoding PD1
) (c:/TeXLive/2020/texmf-dist/tex/generic/intcalc/intcalc.sty
Package: intcalc 2019/12/15 v1.3 Expandable calculations with integers
(HO)
)
\Hy@SavedSpaceFactor=\count266
Package hyperref Info: Option `colorlinks' set `true' on input line 4421.
Package hyperref Info: Hyper figures OFF on input line 4547.
Package hyperref Info: Link nesting OFF on input line 4552.
Package hyperref Info: Hyper index ON on input line 4555.
Package hyperref Info: Plain pages OFF on input line 4562.
Package hyperref Info: Backreferencing OFF on input line 4567.
Package hyperref Info: Implicit mode ON; LaTeX internals redefined.
Package hyperref Info: Bookmarks ON on input line 4800.
\c@Hy@tempcnt=\count267
LaTeX Info: Redefining \url on input line 5159.
\XeTeXLinkMargin=\dimen164
(c:/TeXLive/2020/texmf-dist/tex/generic/bitset/bitset.sty
Package: bitset 2019/12/09 v1.3 Handle bit-vector datatype (HO)
(c:/TeXLive/2020/texmf-dist/tex/generic/bigintcalc/bigintcalc.sty
Package: bigintcalc 2019/12/15 v1.5 Expandable calculations on big
integers (HO
)
))
\Fld@menulength=\count268
```

```
\Field@Width=\dimen165
\Fld@charsize=\dimen166
Package hyperref Info: Hyper figures OFF on input line 6430.
Package hyperref Info: Link nesting OFF on input line 6435.
Package hyperref Info: Hyper index ON on input line 6438.
Package hyperref Info: backreferencing OFF on input line 6445.
Package hyperref Info: Link coloring ON on input line 6448.
Package hyperref Info: Link coloring with OCG OFF on input line 6455.
Package hyperref Info: PDF/A mode OFF on input line 6460.
LaTeX Info: Redefining \ref on input line 6500.
LaTeX Info: Redefining \pageref on input line 6504.
\Hy@abspage=\count269
\c@Item=\count270
\c@Hfootnote=\count271
)
Package hyperref Info: Driver (autodetected): hpdftex.
(c:/TeXLive/2020/texmf-dist/tex/latex/hyperref/hpdftex.def
File: hpdftex.def 2020/01/14 v7.00d Hyperref driver for pdfTeX
(c:/TeXLive/2020/texmf-dist/tex/latex/atveryend/atveryend.sty
Package: atveryend 2019-12-11 v1.11 Hooks at the very end of document
(HO)
)
\HyAnn@Count=\count272
\Fld@listcount=\count273
\c@bookmark@seq@number=\count274
(c:/TeXLive/2020/texmf-dist/tex/latex/rerunfilecheck/rerunfilecheck.sty
Package: rerunfilecheck 2019/12/05 v1.9 Rerun checks for auxiliary files
(HO)
(c:/TeXLive/2020/texmf-dist/tex/generic/uniquecounter/uniquecounter.sty
Package: uniquecounter 2019/12/15 v1.4 Provide unlimited unique counter
(HO)
)
Package uniquecounter Info: New unique counter `rerunfilecheck' on input
line 2
86.
)
\Hy@SectionHShift=\skip141
) (c:/TeXLive/2020/texmf-dist/tex/latex/preprint/authblk.sty
Package: authblk 2001/02/27 1.3 (PWD)
\affilsep=\skip142
\@affilsep=\skip143
\c@Maxaffil=\count275
\c@authors=\count276
\c@affil=\count277
) (c:/TeXLive/2020/texmf-dist/tex/latex/footmisc/footmisc.sty
Package: footmisc 2011/06/06 v5.5b a miscellany of footnote facilities
\FN@temptoken=\toks33
\footnotemargin=\dimen167
\c@pp@next@reset=\count278
Package footmisc Info: Declaring symbol style bringhurst on input line
855.
Package footmisc Info: Declaring symbol style chicago on input line 863.
Package footmisc Info: Declaring symbol style wiley on input line 872.
```

```
Package footmisc Info: Declaring symbol style lamport-robust on input
line 883.

Package footmisc Info: Declaring symbol style lamport* on input line 903.
Package footmisc Info: Declaring symbol style lamport*-robust on input
line 924
.
) (c:/TeXLive/2020/texmf-dist/tex/latex/fancyhdr/fancyhdr.sty
Package: fancyhdr 2019/01/31 v3.10 Extensive control of page headers and
footer
s
\f@nch@headwidth=\skip144
\f@nch@O@elh=\skip145
\f@nch@O@erh=\skip146
\f@nch@O@olh=\skip147
\f@nch@O@orh=\skip148
\f@nch@O@elf=\skip149
\f@nch@O@erf=\skip150
\f@nch@O@olf=\skip151
\f@nch@O@orf=\skip152
) (c:/TeXLive/2020/texmf-dist/tex/generic/alphalph/alphalph.sty
Package: alphalph 2019/12/09 v2.6 Convert numbers to letters (HO)
)
\c@authorfn=\count279
(c:/TeXLive/2020/texmf-dist/tex/latex/abstract/abstract.sty
Package: abstract 2009/06/08 v1.2a configurable abstracts
\abstitleskip=\skip153
\absleftindent=\skip154
\absrightindent=\skip155
\absparindent=\skip156
\absparsep=\skip157
)
Package newfloat Info: New float `keypoints' with options
`placement=t!,name=kp
t' on input line 286.
\c@keypoints=\count280
\newfloat@ftype=\count281
Package newfloat Info: float type `keypoints'=8 on input line 286.
(c:/TeXLive/2020/texmf-dist/tex/latex/enumitem/enumitem.sty
Package: enumitem 2019/06/20 v3.9 Customized lists
\labelindent=\skip158
\enit@outerparindent=\dimen168
\enit@toks=\toks34
\enit@inbox=\box59
\enit@count@id=\count282
\enitdp@description=\count283
) (c:/TeXLive/2020/texmf-dist/tex/latex/quoting/quoting.sty
Package: quoting 2014/01/28 v0.1c Consolidated environment for displayed
text
\quo@toppartop=\skip159
) (c:/TeXLive/2020/texmf-dist/tex/latex/sttools/stfloats.sty
Package: stfloats 2017/03/27 v3.3 Improve float mechanism and
baselineskip sett
ings
```

```
\@dblbotnum=\count284
\c@dblbotnumber=\count285
) (c:/TeXLive/2020/texmf-dist/tex/latex/booktabs/booktabs.sty
Package: booktabs 2020/01/12 v1.61803398 Publication quality tables
\heavyrulewidth=\dimen169
\lightrulewidth=\dimen170
\cmidrulewidth=\dimen171
\belowrulesep=\dimen172
\belowbottomsep=\dimen173
\aboverulesep=\dimen174
\abovetopsep=\dimen175
\cmidrulesep=\dimen176
\cmidrulekern=\dimen177
\defaultaddspace=\dimen178
\@cmidla=\count286
\@cmidlb=\count287
\@aboverulesep=\dimen179
\@belowrulesep=\dimen180
\@thisruleclass=\count288
\@lastruleclass=\count289
\@thisrulewidth=\dimen181
) (c:/TeXLive/2020/texmf-dist/tex/latex/tools/tabularx.sty
Package: tabularx 2020/01/15 v2.11c `tabularx' package (DPC)
\TX@col@width=\dimen182
\TX@old@table=\dimen183
\TX@old@col=\dimen184
\TX@target=\dimen185
\TX@delta=\dimen186
\TX@cols=\count290
\TX@ftn=\toks35
)
\enitdp@tablenotes=\count291
(c:/TeXLive/2020/texmf-dist/tex/latex/caption/caption.sty
Package: caption 2020/01/03 v3.4h Customizing captions (AR)
(c:/TeXLive/2020/texmf-dist/tex/latex/caption/caption3.sty
Package: caption3 2020/01/03 v1.8h caption3 kernel (AR)
Package caption3 Info: TeX engine: e-TeX on input line 61.
\captionmargin=\dimen187
\captionmargin@=\dimen188
\captionwidth=\dimen189
\caption@tempdima=\dimen190
\caption@indent=\dimen191
\caption@parindent=\dimen192
\caption@hangindent=\dimen193
Package caption Info: Standard document class detected.
)
\c@caption@flags=\count292
\c@continuedfloat=\count293
Package caption Info: hyperref package is loaded.
Package caption Info: rotating package is loaded.
) (c:/TeXLive/2020/texmf-dist/tex/latex/natbib/natbib.sty
Package: natbib 2010/09/13 8.31b (PWD, AO)
\bibhang=\skip160
\bibsep=\skip161
```

```
LaTeX Info: Redefining \cite on input line 694.
\c@NAT@ctr=\count294
)) (c:/TeXLive/2020/texmf-dist/tex/latex/siunitx/siunitx.sty
Package: siunitx 2020/02/25 v2.8b A comprehensive (SI) units package
(c:/TeXLive/2020/texmf-dist/tex/latex/amsmath/amstext.sty
Package: amstext 2000/06/29 v2.01 AMS text
(c:/TeXLive/2020/texmf-dist/tex/latex/amsmath/amsgen.sty
File: amsgen.sty 1999/11/30 v2.0 generic functions
\@emptytoks=\toks36
\ex@=\dimen194
)) (c:/TeXLive/2020/texmf-dist/tex/latex/l3packages/l3keys2e/l3keys2e.sty
Package: l3keys2e 2020-03-06 LaTeX2e option processing using LaTeX3 keys
)
\l__siunitx_tmp_box=\box60
\l__siunitx_tmp_dim=\dimen195
\l__siunitx_tmp_int=\count295
\l__siunitx_number_mantissa_length_int=\count296
\l__siunitx_number_uncert_length_int=\count297
\l__siunitx_round_int=\count298
\l__siunitx_process_decimal_int=\count299
\l__siunitx_process_uncertainty_int=\count300
\l__siunitx_process_fixed_int=\count301
\l__siunitx_process_integer_min_int=\count302
\l__siunitx_process_precision_int=\count303
\l__siunitx_group_min_int=\count304
\l__siunitx_angle_marker_box=\box61
\l__siunitx_angle_unit_box=\box62
\l__siunitx_angle_marker_dim=\dimen196
\l__siunitx_angle_unit_dim=\dimen197
\l__siunitx_unit_int=\count305
\l__siunitx_unit_denominator_int=\count306
\l__siunitx_unit_numerator_int=\count307
\l__siunitx_unit_prefix_int=\count308
\l__siunitx_unit_prefix_base_int=\count309
\l__siunitx_unit_prefix_gram_int=\count310
\l__siunitx_number_product_int=\count311
\c__siunitx_one_fill_skip=\skip162
\l__siunitx_table_unit_align_skip=\skip163
\l__siunitx_table_exponent_dim=\dimen198
\l__siunitx_table_integer_dim=\dimen199
\l__siunitx_table_mantissa_dim=\dimen256
\l__siunitx_table_marker_dim=\dimen257
\l__siunitx_table_result_dim=\dimen258
\l__siunitx_table_uncert_dim=\dimen259
\l__siunitx_table_fill_pre_dim=\dimen260
\l__siunitx_table_fill_post_dim=\dimen261
\l__siunitx_table_fill_mid_dim=\dimen262
\l__siunitx_table_pre_box=\box63
\l__siunitx_table_post_box=\box64
\l__siunitx_table_mantissa_box=\box65
\l__siunitx_table_result_box=\box66
\l__siunitx_table_number_align_skip=\skip164
\l__siunitx_table_text_align_skip=\skip165
(c:/TeXLive/2020/texmf-dist/tex/latex/translator/translator.sty
```

```
Package: translator 2019-05-31 v1.12a Easy translation of strings in
LaTeX
)) (c:/TeXLive/2020/texmf-dist/tex/latex/academicons/academicons.sty
Package: academicons 2018/06/27 v1.8.6-2 Academicons Icons
) (./main.aux)
\openout1 = `main.aux'.

LaTeX Font Info:    Checking defaults for OML/cmm/m/it on input line 46.
LaTeX Font Info:    ... okay on input line 46.
LaTeX Font Info:    Checking defaults for OMS/cmsy/m/n on input line 46.
LaTeX Font Info:    ... okay on input line 46.
LaTeX Font Info:    Checking defaults for OT1/cmr/m/n on input line 46.
LaTeX Font Info:    ... okay on input line 46.
LaTeX Font Info:    Checking defaults for T1/cmr/m/n on input line 46.
LaTeX Font Info:    ... okay on input line 46.
LaTeX Font Info:    Checking defaults for TS1/cmr/m/n on input line 46.
LaTeX Font Info:    ... okay on input line 46.
LaTeX Font Info:    Checking defaults for OMX/cmex/m/n on input line 46.
LaTeX Font Info:    ... okay on input line 46.
LaTeX Font Info:    Checking defaults for U/cmr/m/n on input line 46.
LaTeX Font Info:    ... okay on input line 46.
LaTeX Font Info:    Checking defaults for PD1/pdf/m/n on input line 46.
LaTeX Font Info:    ... okay on input line 46.
LaTeX Font Info:    Trying to load font information for T1+Merriweather-
OsF on
input line 46.
(c:/TeXLive/2020/texmf-dist/tex/latex/merriweather/T1Merriweather-OsF.fd
File: T1Merriweather-OsF.fd 2019/06/02 (autoinst) Font definitions for
T1/Merri
weather-OsF.
)
LaTeX Font Info:    Font shape `T1/Merriweather-OsF/m/n' in size <7.5>
not avai
lable
(Font)              Font shape `T1/Merriweather-OsF/regular/n' tried
instead on
 input line 46.
LaTeX Font Info:    Font shape `T1/Merriweather-OsF/regular/n' will be
(Font)              scaled to size 7.5pt on input line 46.
LaTeX Info: Redefining \microtypecontext on input line 46.
Package microtype Info: Generating PDF output.
Package microtype Info: Character protrusion enabled (level 2).
Package microtype Info: Using default protrusion set `alltext'.
Package microtype Info: Automatic font expansion enabled (level 2),
(microtype)             stretch: 20, shrink: 20, step: 1, non-selected.
Package microtype Info: Using default expansion set `basictext'.
LaTeX Info: Redefining \showhyphens on input line 46.
Package microtype Info: No adjustment of tracking.
Package microtype Info: No adjustment of interword spacing.
Package microtype Info: No adjustment of character kerning.
Package microtype Info: Loading generic protrusion settings for font
family
(microtype)             `Merriweather-OsF' (encoding: T1).
```

```
(microtype)              For optimal results, create family-specific
settings.
(microtype)              See the microtype manual for details.
LaTeX Font Info:    Redeclaring symbol font `operators' on input line 46.
LaTeX Font Info:    Encoding `OT1' has changed to `T1' for symbol font
(Font)              `operators' in the math version `normal' on input
line 46.
LaTeX Font Info:    Overwriting symbol font `operators' in version
`normal'
(Font)                  OT1/cmr/m/n --> T1/Merriweather-OsF/m/up on input
line
46.
LaTeX Font Info:    Encoding `OT1' has changed to `T1' for symbol font
(Font)              `operators' in the math version `bold' on input line
46.
LaTeX Font Info:    Overwriting symbol font `operators' in version `bold'
(Font)                  OT1/cmr/bx/n --> T1/Merriweather-OsF/m/up on
input line
 46.
LaTeX Font Info:    Overwriting symbol font `operators' in version `bold'
(Font)                  T1/Merriweather-OsF/m/up --> T1/Merriweather-
OsF/b/up o
n input line 46.
LaTeX Font Info:    Redeclaring math alphabet \mathbf on input line 46.
LaTeX Font Info:    Overwriting math alphabet `\mathbf' in version
`normal'
(Font)                  OT1/cmr/bx/n --> T1/Merriweather-OsF/b/up on
input line
 46.
LaTeX Font Info:    Overwriting math alphabet `\mathbf' in version `bold'
(Font)                  OT1/cmr/bx/n --> T1/Merriweather-OsF/b/up on
input line
 46.
LaTeX Font Info:    Redeclaring math alphabet \mathsf on input line 46.
LaTeX Font Info:    Overwriting math alphabet `\mathsf' in version
`normal'
(Font)                  OT1/cmss/m/n --> T1/MerriweatherSans-OsF/m/up on
input
line 46.
LaTeX Font Info:    Overwriting math alphabet `\mathsf' in version `bold'
(Font)                  OT1/cmss/bx/n --> T1/MerriweatherSans-OsF/m/up on
input
 line 46.
LaTeX Font Info:    Redeclaring math alphabet \mathit on input line 46.
LaTeX Font Info:    Overwriting math alphabet `\mathit' in version
`normal'
(Font)                  OT1/cmr/m/it --> T1/Merriweather-OsF/m/it on
input line
 46.
LaTeX Font Info:    Overwriting math alphabet `\mathit' in version `bold'
(Font)                  OT1/cmr/bx/it --> T1/Merriweather-OsF/m/it on
input lin
e 46.
LaTeX Font Info:    Redeclaring math alphabet \mathtt on input line 46.
```

```
LaTeX Font Info:    Overwriting math alphabet `\mathtt' in version
`normal'
(Font)                  OT1/cmtt/m/n --> T1/lmtt/m/up on input line 46.
LaTeX Font Info:    Overwriting math alphabet `\mathtt' in version `bold'
(Font)                  OT1/cmtt/m/n --> T1/lmtt/m/up on input line 46.
LaTeX Font Info:    Overwriting math alphabet `\mathsf' in version `bold'
(Font)                  T1/MerriweatherSans-OsF/m/up -->
T1/MerriweatherSans-Os
F/b/up on input line 46.
LaTeX Font Info:    Overwriting math alphabet `\mathit' in version `bold'
(Font)                  T1/Merriweather-OsF/m/it --> T1/Merriweather-
OsF/b/it o
n input line 46.
\c@mv@tabular=\count312
\c@mv@boldtabular=\count313
ABD: EverySelectfont initializing macros
LaTeX Info: Redefining \selectfont on input line 46.
(c:/TeXLive/2020/texmf-dist/tex/context/base/mkii/supp-pdf.mkii
[Loading MPS to PDF converter (version 2006.09.02).]
\scratchcounter=\count314
\scratchdimen=\dimen263
\scratchbox=\box67
\nofMPsegments=\count315
\nofMParguments=\count316
\everyMPshowfont=\toks37
\MPscratchCnt=\count317
\MPscratchDim=\dimen264
\MPnumerator=\count318
\makeMPintoPDFobject=\count319
\everyMPtoPDFconversion=\toks38
) (c:/TeXLive/2020/texmf-dist/tex/latex/epstopdf-pkg/epstopdf-base.sty
Package: epstopdf-base 2020-01-24 v2.11 Base part for package epstopdf
Package epstopdf-base Info: Redefining graphics rule for `.eps' on input
line 4
85.
(c:/TeXLive/2020/texmf-dist/tex/latex/latexconfig/epstopdf-sys.cfg
File: epstopdf-sys.cfg 2010/07/13 v1.3 Configuration of (r)epstopdf for
TeX Liv
e
))
Package lastpage Info: Please have a look at the pageslts package at
(lastpage)             https://www.ctan.org/pkg/pageslts
(lastpage)             ! on input line 46.
ABD: EveryShipout initializing macros
\AtBeginShipoutBox=\box68
*geometry* driver: auto-detecting
*geometry* detected driver: pdftex
*geometry* verbose mode - [ preamble ] result:
* driver: pdftex
* paper: a4paper
* layout: <same size as paper>
* layoutoffset:(h,v)=(0.0pt,0.0pt)
* modes: includefoot twoside
* h-part:(L,W,R)=(54.64pt, 488.22787pt, 54.64pt)
```

```
* v-part:(T,H,B)=(66.0pt, 745.04684pt, 34.0pt)
* \paperwidth=597.50787pt
* \paperheight=845.04684pt
* \textwidth=488.22787pt
* \textheight=715.04684pt
* \oddsidemargin=-17.62999pt
* \evensidemargin=-17.62999pt
* \topmargin=-47.76999pt
* \headheight=17.5pt
* \headsep=24.0pt
* \topskip=10.0pt
* \footskip=30.0pt
* \marginparwidth=48.0pt
* \marginparsep=10.0pt
* \columnsep=18.0pt
* \skip\footins=22.0pt plus 2.0pt
* \hoffset=0.0pt
* \voffset=0.0pt
* \mag=1000
* \@twocolumntrue
* \@twosidetrue
* \@mparswitchtrue
* \@reversemarginfalse
* (1in=72.27pt=25.4mm, 1cm=28.453pt)

Package hyperref Info: Link coloring ON on input line 46.
(c:/TeXLive/2020/texmf-dist/tex/latex/hyperref/nameref.sty
Package: nameref 2019/09/16 v2.46 Cross-referencing by name of section
(c:/TeXLive/2020/texmf-dist/tex/latex/refcount/refcount.sty
Package: refcount 2019/12/15 v3.6 Data extraction from label references
(HO)
) (c:/TeXLive/2020/texmf-
dist/tex/generic/gettitlestring/gettitlestring.sty
Package: gettitlestring 2019/12/15 v1.6 Cleanup title references (HO)
)
\c@section@level=\count320
)
LaTeX Info: Redefining \ref on input line 46.
LaTeX Info: Redefining \pageref on input line 46.
LaTeX Info: Redefining \nameref on input line 46.
(./main.out) (./main.out)
\@outlinefile=\write3
\openout3 = `main.out'.

\@gscitedetails=\box69
\@gscitedetailsheight=\skip166
\@gsheadbox=\box70
\@gsheadboxheight=\skip167
LaTeX Font Info:    Font shape `T1/Merriweather-OsF/b/n' in size <6.5>
not avai
lable
(Font)              Font shape `T1/Merriweather-OsF/bold/n' tried instead
on in
put line 46.
```

```
LaTeX Font Info:    Font shape `T1/Merriweather-OsF/bold/n' will be
(Font)              scaled to size 6.5pt on input line 46.
LaTeX Font Info:    Calculating math sizes for size <7.5> on input line
46.

LaTeX Font Warning: Font shape `T1/Merriweather-OsF/m/up' undefined
(Font)              using `T1/Merriweather-OsF/m/n' instead on input line
46.

LaTeX Font Info:    Font shape `T1/Merriweather-OsF/m/up' in size
<6.24973> not
 available
(Font)              Font shape `T1/Merriweather-OsF/regular/n' tried
instead on
 input line 46.
LaTeX Font Info:    Font shape `T1/Merriweather-OsF/regular/n' will be
(Font)              scaled to size 6.24973pt on input line 46.
LaTeX Font Info:    Font shape `T1/Merriweather-OsF/m/up' in size
<5.24997> not
 available
(Font)              Font shape `T1/Merriweather-OsF/regular/n' tried
instead on
 input line 46.
LaTeX Font Info:    Font shape `T1/Merriweather-OsF/regular/n' will be
(Font)              scaled to size 5.24997pt on input line 46.
LaTeX Font Info:    Trying to load font information for U+eur on input
line 46.

(c:/TeXLive/2020/texmf-dist/tex/latex/amsfonts/ueur.fd
File: ueur.fd 2013/01/14 v3.01 Euler Roman
) (c:/TeXLive/2020/texmf-dist/tex/latex/microtype/mt-eur.cfg
File: mt-eur.cfg 2006/07/31 v1.1 microtype config. file: AMS Euler Roman
(RS)
)

LaTeX Font Warning: Font shape `OMS/cmsy/m/n' in size <7.5> not available
(Font)              size <7> substituted on input line 46.

LaTeX Font Info:    External font `cmex10' loaded for size
(Font)              <7.5> on input line 46.
LaTeX Font Info:    External font `cmex10' loaded for size
(Font)              <6.24973> on input line 46.
LaTeX Font Info:    External font `cmex10' loaded for size
(Font)              <5.24997> on input line 46.
LaTeX Font Info:    Trying to load font information for U+euf on input
line 46.

(c:/TeXLive/2020/texmf-dist/tex/latex/amsfonts/ueuf.fd
File: ueuf.fd 2013/01/14 v3.01 Euler Fraktur
) (c:/TeXLive/2020/texmf-dist/tex/latex/microtype/mt-euf.cfg
File: mt-euf.cfg 2006/07/03 v1.1 microtype config. file: AMS Euler
Fraktur (RS)

)
```

```
LaTeX Font Info:    Trying to load font information for U+eus on input
line 46.

(c:/TeXLive/2020/texmf-dist/tex/latex/amsfonts/ueus.fd
File: ueus.fd 2013/01/14 v3.01 Euler Script
) (c:/TeXLive/2020/texmf-dist/tex/latex/microtype/mt-eus.cfg
File: mt-eus.cfg 2006/07/28 v1.2 microtype config. file: AMS Euler Script
(RS)
)
LaTeX Font Info:    Trying to load font information for U+euex on input
line 46
.
(c:/TeXLive/2020/texmf-dist/tex/latex/amsfonts/ueuex.fd
File: ueuex.fd 2013/01/14 v3.01 Euler extra symbols
)

LaTeX Font Warning: Font shape `OML/cmm/m/it' in size <7.5> not available
(Font)              size <7> substituted on input line 46.

LaTeX Font Info:    Font shape `T1/Merriweather-OsF/m/n' in size
<6.24973> not
available
(Font)              Font shape `T1/Merriweather-OsF/regular/n' tried
instead on
 input line 46.
LaTeX Font Info:    Font shape `T1/Merriweather-OsF/regular/n' will be
(Font)              scaled to size 6.24973pt on input line 46.
LaTeX Font Info:    Font shape `T1/Merriweather-OsF/m/n' in size
<5.24997> not
available
(Font)              Font shape `T1/Merriweather-OsF/regular/n' tried
instead on
 input line 46.
LaTeX Font Info:    Font shape `T1/Merriweather-OsF/regular/n' will be
(Font)              scaled to size 5.24997pt on input line 46.
LaTeX Font Info:    Font shape `T1/Merriweather-OsF/m/it' in size <7.5>
not ava
ilable
(Font)              Font shape `T1/Merriweather-OsF/regular/it' tried
instead o
n input line 46.
LaTeX Font Info:    Font shape `T1/Merriweather-OsF/regular/it' will be
(Font)              scaled to size 7.5pt on input line 46.
LaTeX Font Info:    Font shape `T1/Merriweather-OsF/m/it' in size
<6.24973> not
 available
(Font)              Font shape `T1/Merriweather-OsF/regular/it' tried
instead o
n input line 46.
LaTeX Font Info:    Font shape `T1/Merriweather-OsF/regular/it' will be
(Font)              scaled to size 6.24973pt on input line 46.
LaTeX Font Info:    Font shape `T1/Merriweather-OsF/m/it' in size
<5.24997> not
 available
```

```
(Font)              Font shape `T1/Merriweather-OsF/regular/it' tried
instead o
n input line 46.
LaTeX Font Info:    Font shape `T1/Merriweather-OsF/regular/it' will be
(Font)              scaled to size 5.24997pt on input line 46.
LaTeX Font Info:    Font shape `T1/Merriweather-OsF/m/n' in size <8> not
availa
ble
(Font)              Font shape `T1/Merriweather-OsF/regular/n' tried
instead on
 input line 46.
LaTeX Font Info:    Font shape `T1/Merriweather-OsF/regular/n' will be
(Font)              scaled to size 8.0pt on input line 46.
LaTeX Font Info:    Font shape `T1/Merriweather-OsF/m/it' in size <8> not
avail
able
(Font)              Font shape `T1/Merriweather-OsF/regular/it' tried
instead o
n input line 46.
LaTeX Font Info:    Font shape `T1/Merriweather-OsF/regular/it' will be
(Font)              scaled to size 8.0pt on input line 46.
LaTeX Font Info:    Font shape `T1/Merriweather-OsF/b/it' in size <8> not
avail
able
(Font)              Font shape `T1/Merriweather-OsF/bold/it' tried
instead on i
nput line 46.
LaTeX Font Info:    Font shape `T1/Merriweather-OsF/bold/it' will be
(Font)              scaled to size 8.0pt on input line 46.
Package caption Info: Begin \AtBeginDocument code.
Package caption Info: End \AtBeginDocument code.

(c:/TeXLive/2020/texmf-dist/tex/latex/translator/translator-basic-
dictionary-En
glish.dict
Dictionary: translator-basic-dictionary, Language: English
) (c:/TeXLive/2020/texmf-dist/tex/latex/siunitx/siunitx-abbreviations.cfg
File: siunitx-abbreviations.cfg 2017/11/26 v2.7k siunitx: Abbreviated
units
)
LaTeX Font Info:    Trying to load font information for
T1+MerriweatherSans-OsF
 on input line 46.
(c:/TeXLive/2020/texmf-dist/tex/latex/merriweather/T1MerriweatherSans-
OsF.fd
File: T1MerriweatherSans-OsF.fd 2019/06/02 (autoinst) Font definitions
for T1/M
erriweatherSans-OsF.
)
LaTeX Font Info:    Font shape `T1/MerriweatherSans-OsF/m/n' in size
<7.5> not
available
(Font)              Font shape `T1/MerriweatherSans-OsF/regular/n' tried
instea
```

```
d on input line 46.
LaTeX Font Info:    Font shape `T1/MerriweatherSans-OsF/regular/n' will
be
(Font)              scaled to size 7.5pt on input line 46.
Package microtype Info: Loading generic protrusion settings for font
family
(microtype)             `MerriweatherSans-OsF' (encoding: T1).
(microtype)             For optimal results, create family-specific
settings.
(microtype)             See the microtype manual for details.
LaTeX Font Info:    Font shape `T1/MerriweatherSans-OsF/m/n' in size
<6.24973>
not available
(Font)              Font shape `T1/MerriweatherSans-OsF/regular/n' tried
instea
d on input line 46.
LaTeX Font Info:    Font shape `T1/MerriweatherSans-OsF/regular/n' will
be
(Font)              scaled to size 6.24973pt on input line 46.
LaTeX Font Info:    Font shape `T1/MerriweatherSans-OsF/m/n' in size
<5.24997>
not available
(Font)              Font shape `T1/MerriweatherSans-OsF/regular/n' tried
instea
d on input line 46.
LaTeX Font Info:    Font shape `T1/MerriweatherSans-OsF/regular/n' will
be
(Font)              scaled to size 5.24997pt on input line 46.
LaTeX Font Info:    Trying to load font information for T1+lmtt on input
line 4
6.
(c:/TeXLive/2020/texmf-dist/tex/latex/lm/t1lmtt.fd
File: t1lmtt.fd 2009/10/30 v1.6 Font defs for Latin Modern
)
Package microtype Info: Loading generic protrusion settings for font
family
(microtype)             `lmtt' (encoding: T1).
(microtype)             For optimal results, create family-specific
settings.
(microtype)             See the microtype manual for details.
TextBlockOrigin set to 4pc+6.64pt x 4pc+6pt

Overfull \hbox (54.64pt too wide) in paragraph at lines 66--66
[][]
 []

LaTeX Font Info:    Font shape `T1/Merriweather-OsF/m/n' in size <14> not
avail
able
(Font)              Font shape `T1/Merriweather-OsF/regular/n' tried
instead on
 input line 66.
LaTeX Font Info:    Font shape `T1/Merriweather-OsF/regular/n' will be
(Font)              scaled to size 14.0pt on input line 66.
```

```
LaTeX Font Info:    Font shape `T1/Merriweather-OsF/m/n' in size
<8.99997> not
available
(Font)              Font shape `T1/Merriweather-OsF/regular/n' tried
instead on
 input line 66.
LaTeX Font Info:    Font shape `T1/Merriweather-OsF/regular/n' will be
(Font)              scaled to size 8.99997pt on input line 66.
LaTeX Font Info:    Calculating math sizes for size <14> on input line
66.
LaTeX Font Info:    Font shape `T1/Merriweather-OsF/m/up' in size <14>
not avai
lable
(Font)              Font shape `T1/Merriweather-OsF/regular/n' tried
instead on
 input line 66.
LaTeX Font Info:    Font shape `T1/Merriweather-OsF/regular/n' will be
(Font)              scaled to size 14.0pt on input line 66.
LaTeX Font Info:    Font shape `T1/Merriweather-OsF/m/up' in size
<11.66617> no
t available
(Font)              Font shape `T1/Merriweather-OsF/regular/n' tried
instead on
 input line 66.
LaTeX Font Info:    Font shape `T1/Merriweather-OsF/regular/n' will be
(Font)              scaled to size 11.66617pt on input line 66.
LaTeX Font Info:    Font shape `T1/Merriweather-OsF/m/up' in size
<9.79996> not
 available
(Font)              Font shape `T1/Merriweather-OsF/regular/n' tried
instead on
 input line 66.
LaTeX Font Info:    Font shape `T1/Merriweather-OsF/regular/n' will be
(Font)              scaled to size 9.79996pt on input line 66.
LaTeX Font Info:    External font `cmex10' loaded for size
(Font)              <14> on input line 66.
LaTeX Font Info:    External font `cmex10' loaded for size
(Font)              <11.66617> on input line 66.
LaTeX Font Info:    External font `cmex10' loaded for size
(Font)              <9.79996> on input line 66.
LaTeX Font Info:    Font shape `T1/Merriweather-OsF/m/n' in size
<11.66617> not
 available
(Font)              Font shape `T1/Merriweather-OsF/regular/n' tried
instead on
 input line 66.
LaTeX Font Info:    Font shape `T1/Merriweather-OsF/regular/n' will be
(Font)              scaled to size 11.66617pt on input line 66.
LaTeX Font Info:    Font shape `T1/Merriweather-OsF/m/n' in size
<9.79996> not
available
(Font)              Font shape `T1/Merriweather-OsF/regular/n' tried
instead on
 input line 66.
```

```
LaTeX Font Info:    Font shape `T1/Merriweather-OsF/regular/n' will be
(Font)              scaled to size 9.79996pt on input line 66.
LaTeX Font Info:    Font shape `T1/Merriweather-OsF/m/it' in size <14>
not avai
lable
(Font)              Font shape `T1/Merriweather-OsF/regular/it' tried
instead o
n input line 66.
LaTeX Font Info:    Font shape `T1/Merriweather-OsF/regular/it' will be
(Font)              scaled to size 14.0pt on input line 66.
LaTeX Font Info:    Font shape `T1/Merriweather-OsF/m/it' in size
<11.66617> no
t available
(Font)              Font shape `T1/Merriweather-OsF/regular/it' tried
instead o
n input line 66.
LaTeX Font Info:    Font shape `T1/Merriweather-OsF/regular/it' will be
(Font)              scaled to size 11.66617pt on input line 66.
LaTeX Font Info:    Font shape `T1/Merriweather-OsF/m/it' in size
<9.79996> not
 available
(Font)              Font shape `T1/Merriweather-OsF/regular/it' tried
instead o
n input line 66.
LaTeX Font Info:    Font shape `T1/Merriweather-OsF/regular/it' will be
(Font)              scaled to size 9.79996pt on input line 66.
LaTeX Font Info:    Font shape `T1/MerriweatherSans-OsF/m/n' in size <14>
not a
vailable
(Font)              Font shape `T1/MerriweatherSans-OsF/regular/n' tried
instea
d on input line 66.
LaTeX Font Info:    Font shape `T1/MerriweatherSans-OsF/regular/n' will
be
(Font)              scaled to size 14.0pt on input line 66.
LaTeX Font Info:    Font shape `T1/MerriweatherSans-OsF/m/n' in size
<11.66617>
 not available
(Font)              Font shape `T1/MerriweatherSans-OsF/regular/n' tried
instea
d on input line 66.
LaTeX Font Info:    Font shape `T1/MerriweatherSans-OsF/regular/n' will
be
(Font)              scaled to size 11.66617pt on input line 66.
LaTeX Font Info:    Font shape `T1/MerriweatherSans-OsF/m/n' in size
<9.79996>
not available
(Font)              Font shape `T1/MerriweatherSans-OsF/regular/n' tried
instea
d on input line 66.
LaTeX Font Info:    Font shape `T1/MerriweatherSans-OsF/regular/n' will
be
(Font)              scaled to size 9.79996pt on input line 66.
```

```
LaTeX Font Info:    Font shape `T1/Merriweather-OsF/b/n' in size <18> not avail
able
(Font)              Font shape `T1/Merriweather-OsF/bold/n' tried instead on in
put line 66.
LaTeX Font Info:    Font shape `T1/Merriweather-OsF/bold/n' will be
(Font)              scaled to size 18.0pt on input line 66.
LaTeX Font Info:    Font shape `T1/Merriweather-OsF/m/n' in size <13> not avail
able
(Font)              Font shape `T1/Merriweather-OsF/regular/n' tried instead on
 input line 66.
LaTeX Font Info:    Font shape `T1/Merriweather-OsF/regular/n' will be
(Font)              scaled to size 13.0pt on input line 66.

! LaTeX Error: Encoding scheme `TU' unknown.

See the LaTeX manual or LaTeX Companion for explanation.
Type  H <return>  for immediate help.
 ...

l.66 \end{frontmatter}

Your command was ignored.
Type  I <command> <return>  to replace it with another command,
or  <return>  to continue without it.

LaTeX Font Info:    Trying to load font information for T1+academicons on input
 line 66.
LaTeX Font Info:    No file T1academicons.fd. on input line 66.

LaTeX Font Warning: Font shape `T1/academicons/m/n' undefined
(Font)              using `T1/cmr/m/n' instead on input line 66.


LaTeX Font Warning: Font shape `T1/cmr/m/n' in size <13> not available
(Font)              size <12> substituted on input line 66.

(c:/TeXLive/2020/texmf-dist/tex/latex/microtype/mt-cmr.cfg
File: mt-cmr.cfg 2013/05/19 v2.2 microtype config. file: Computer Modern Roman
(RS)
)
! Bad character code (59865).
<to be read again>
                   \relax
l.66 \end{frontmatter}

A character number must be between 0 and 255.
I changed this one to zero.
```

```
LaTeX Font Info:    Calculating math sizes for size <13> on input line
66.
LaTeX Font Info:    Font shape `T1/Merriweather-OsF/m/up' in size <13>
not avai
lable
(Font)              Font shape `T1/Merriweather-OsF/regular/n' tried
instead on
 input line 66.
LaTeX Font Info:    Font shape `T1/Merriweather-OsF/regular/n' will be
(Font)              scaled to size 13.0pt on input line 66.
LaTeX Font Info:    Font shape `T1/Merriweather-OsF/m/up' in size
<10.83287> no
t available
(Font)              Font shape `T1/Merriweather-OsF/regular/n' tried
instead on
 input line 66.
LaTeX Font Info:    Font shape `T1/Merriweather-OsF/regular/n' will be
(Font)              scaled to size 10.83287pt on input line 66.
LaTeX Font Info:    Font shape `T1/Merriweather-OsF/m/up' in size
<9.09996> not
 available
(Font)              Font shape `T1/Merriweather-OsF/regular/n' tried
instead on
 input line 66.
LaTeX Font Info:    Font shape `T1/Merriweather-OsF/regular/n' will be
(Font)              scaled to size 9.09996pt on input line 66.

LaTeX Font Warning: Font shape `OMS/cmsy/m/n' in size <13> not available
(Font)              size <12> substituted on input line 66.

LaTeX Font Info:    External font `cmex10' loaded for size
(Font)              <13> on input line 66.
LaTeX Font Info:    External font `cmex10' loaded for size
(Font)              <10.83287> on input line 66.
LaTeX Font Info:    External font `cmex10' loaded for size
(Font)              <9.09996> on input line 66.

LaTeX Font Warning: Font shape `OML/cmm/m/it' in size <13> not available
(Font)              size <12> substituted on input line 66.

LaTeX Font Info:    Font shape `T1/Merriweather-OsF/m/n' in size
<10.83287> not
 available
(Font)              Font shape `T1/Merriweather-OsF/regular/n' tried
instead on
 input line 66.
LaTeX Font Info:    Font shape `T1/Merriweather-OsF/regular/n' will be
(Font)              scaled to size 10.83287pt on input line 66.
LaTeX Font Info:    Font shape `T1/Merriweather-OsF/m/n' in size
<9.09996> not
available
(Font)              Font shape `T1/Merriweather-OsF/regular/n' tried
instead on
 input line 66.
```

```
LaTeX Font Info:    Font shape `T1/Merriweather-OsF/regular/n' will be
(Font)              scaled to size 9.09996pt on input line 66.
LaTeX Font Info:    Font shape `T1/Merriweather-OsF/m/it' in size <13>
not avai
lable
(Font)              Font shape `T1/Merriweather-OsF/regular/it' tried
instead o
n input line 66.
LaTeX Font Info:    Font shape `T1/Merriweather-OsF/regular/it' will be
(Font)              scaled to size 13.0pt on input line 66.
LaTeX Font Info:    Font shape `T1/Merriweather-OsF/m/it' in size
<10.83287> no
t available
(Font)              Font shape `T1/Merriweather-OsF/regular/it' tried
instead o
n input line 66.
LaTeX Font Info:    Font shape `T1/Merriweather-OsF/regular/it' will be
(Font)              scaled to size 10.83287pt on input line 66.
LaTeX Font Info:    Font shape `T1/Merriweather-OsF/m/it' in size
<9.09996> not
 available
(Font)              Font shape `T1/Merriweather-OsF/regular/it' tried
instead o
n input line 66.
LaTeX Font Info:    Font shape `T1/Merriweather-OsF/regular/it' will be
(Font)              scaled to size 9.09996pt on input line 66.
LaTeX Font Info:    Font shape `T1/MerriweatherSans-OsF/m/n' in size <13>
not a
vailable
(Font)              Font shape `T1/MerriweatherSans-OsF/regular/n' tried
instea
d on input line 66.
LaTeX Font Info:    Font shape `T1/MerriweatherSans-OsF/regular/n' will
be
(Font)              scaled to size 13.0pt on input line 66.
LaTeX Font Info:    Font shape `T1/MerriweatherSans-OsF/m/n' in size
<10.83287>
 not available
(Font)              Font shape `T1/MerriweatherSans-OsF/regular/n' tried
instea
d on input line 66.
LaTeX Font Info:    Font shape `T1/MerriweatherSans-OsF/regular/n' will
be
(Font)              scaled to size 10.83287pt on input line 66.
LaTeX Font Info:    Font shape `T1/MerriweatherSans-OsF/m/n' in size
<9.09996>
not available
(Font)              Font shape `T1/MerriweatherSans-OsF/regular/n' tried
instea
d on input line 66.
LaTeX Font Info:    Font shape `T1/MerriweatherSans-OsF/regular/n' will
be
(Font)              scaled to size 9.09996pt on input line 66.
```

```
LaTeX Font Info:    Trying to load font information for TS1+Merriweather-
OsF on
 input line 66.
(c:/TeXLive/2020/texmf-dist/tex/latex/merriweather/TS1Merriweather-OsF.fd
File: TS1Merriweather-OsF.fd 2019/06/02 (autoinst) Font definitions for
TS1/Mer
riweather-OsF.
)
LaTeX Font Info:    Font shape `TS1/Merriweather-OsF/m/n' in size
<10.83287> no
t available
(Font)              Font shape `TS1/Merriweather-OsF/regular/n' tried
instead o
n input line 66.
LaTeX Font Info:    Font shape `TS1/Merriweather-OsF/regular/n' will be
(Font)              scaled to size 10.83287pt on input line 66.
Package microtype Info: Loading generic protrusion settings for font
family
(microtype)             `Merriweather-OsF' (encoding: TS1).
(microtype)             For optimal results, create family-specific
settings.
(microtype)             See the microtype manual for details.

! LaTeX Error: Encoding scheme `TU' unknown.

See the LaTeX manual or LaTeX Companion for explanation.
Type  H <return>  for immediate help.
 ...

l.66 \end{frontmatter}

Your command was ignored.
Type  I <command> <return>  to replace it with another command,
or  <return>  to continue without it.


LaTeX Font Warning: Font shape `T1/academicons/m/n' in size <13> not
available
(Font)              size <12> substituted on input line 66.

Package microtype Info: Loading generic protrusion settings for font
family
(microtype)             `academicons' (encoding: T1).
(microtype)             For optimal results, create family-specific
settings.
(microtype)             See the microtype manual for details.
! Bad character code (59865).
<to be read again>
                   \relax
l.66 \end{frontmatter}

A character number must be between 0 and 255.
I changed this one to zero.
```

```
! LaTeX Error: Encoding scheme `TU' unknown.

See the LaTeX manual or LaTeX Companion for explanation.
Type  H <return>  for immediate help.
 ...

l.66 \end{frontmatter}

Your command was ignored.
Type  I <command> <return>  to replace it with another command,
or  <return>  to continue without it.

! Bad character code (59865).
<to be read again>
                   \relax
l.66 \end{frontmatter}

A character number must be between 0 and 255.
I changed this one to zero.

LaTeX Font Info:    Font shape `T1/Merriweather-OsF/m/n' in size <9> not
availa
ble
(Font)              Font shape `T1/Merriweather-OsF/regular/n' tried
instead on
 input line 66.
LaTeX Font Info:    Font shape `T1/Merriweather-OsF/regular/n' will be
(Font)              scaled to size 9.0pt on input line 66.
LaTeX Font Info:    Font shape `T1/Merriweather-OsF/m/up' in size <9> not
avail
able
(Font)              Font shape `T1/Merriweather-OsF/regular/n' tried
instead on
 input line 66.
LaTeX Font Info:    Font shape `T1/Merriweather-OsF/regular/n' will be
(Font)              scaled to size 9.0pt on input line 66.
LaTeX Font Info:    Font shape `T1/Merriweather-OsF/m/up' in size <7> not
avail
able
(Font)              Font shape `T1/Merriweather-OsF/regular/n' tried
instead on
 input line 66.
LaTeX Font Info:    Font shape `T1/Merriweather-OsF/regular/n' will be
(Font)              scaled to size 7.0pt on input line 66.
LaTeX Font Info:    Font shape `T1/Merriweather-OsF/m/up' in size <5> not
avail
able
(Font)              Font shape `T1/Merriweather-OsF/regular/n' tried
instead on
 input line 66.
LaTeX Font Info:    Font shape `T1/Merriweather-OsF/regular/n' will be
(Font)              scaled to size 5.0pt on input line 66.
LaTeX Font Info:    External font `cmex10' loaded for size
```

```
(Font)              <9> on input line 66.
LaTeX Font Info:    External font `cmex10' loaded for size
(Font)              <7> on input line 66.
LaTeX Font Info:    External font `cmex10' loaded for size
(Font)              <5> on input line 66.
LaTeX Font Info:    Font shape `T1/Merriweather-OsF/m/n' in size <7> not
availa
ble
(Font)              Font shape `T1/Merriweather-OsF/regular/n' tried
instead on
 input line 66.
LaTeX Font Info:    Font shape `T1/Merriweather-OsF/regular/n' will be
(Font)              scaled to size 7.0pt on input line 66.
LaTeX Font Info:    Font shape `T1/Merriweather-OsF/m/n' in size <5> not
availa
ble
(Font)              Font shape `T1/Merriweather-OsF/regular/n' tried
instead on
 input line 66.
LaTeX Font Info:    Font shape `T1/Merriweather-OsF/regular/n' will be
(Font)              scaled to size 5.0pt on input line 66.
LaTeX Font Info:    Font shape `T1/Merriweather-OsF/m/it' in size <9> not
avail
able
(Font)              Font shape `T1/Merriweather-OsF/regular/it' tried
instead o
n input line 66.
LaTeX Font Info:    Font shape `T1/Merriweather-OsF/regular/it' will be
(Font)              scaled to size 9.0pt on input line 66.
LaTeX Font Info:    Font shape `T1/Merriweather-OsF/m/it' in size <7> not
avail
able
(Font)              Font shape `T1/Merriweather-OsF/regular/it' tried
instead o
n input line 66.
LaTeX Font Info:    Font shape `T1/Merriweather-OsF/regular/it' will be
(Font)              scaled to size 7.0pt on input line 66.
LaTeX Font Info:    Font shape `T1/Merriweather-OsF/m/it' in size <5> not
avail
able
(Font)              Font shape `T1/Merriweather-OsF/regular/it' tried
instead o
n input line 66.
LaTeX Font Info:    Font shape `T1/Merriweather-OsF/regular/it' will be
(Font)              scaled to size 5.0pt on input line 66.
LaTeX Font Info:    Font shape `T1/MerriweatherSans-OsF/m/n' in size <9>
not av
ailable
(Font)              Font shape `T1/MerriweatherSans-OsF/regular/n' tried
instea
d on input line 66.
LaTeX Font Info:    Font shape `T1/MerriweatherSans-OsF/regular/n' will
be
(Font)              scaled to size 9.0pt on input line 66.
```

```
LaTeX Font Info:    Font shape `T1/MerriweatherSans-OsF/m/n' in size <7>
not av
ailable
(Font)              Font shape `T1/MerriweatherSans-OsF/regular/n' tried
instea
d on input line 66.
LaTeX Font Info:    Font shape `T1/MerriweatherSans-OsF/regular/n' will
be
(Font)              scaled to size 7.0pt on input line 66.
LaTeX Font Info:    Font shape `T1/MerriweatherSans-OsF/m/n' in size <5>
not av
ailable
(Font)              Font shape `T1/MerriweatherSans-OsF/regular/n' tried
instea
d on input line 66.
LaTeX Font Info:    Font shape `T1/MerriweatherSans-OsF/regular/n' will
be
(Font)              scaled to size 5.0pt on input line 66.
LaTeX Font Info:    Font shape `T1/Merriweather-OsF/m/n' in size <6.5>
not avai
lable
(Font)              Font shape `T1/Merriweather-OsF/regular/n' tried
instead on
 input line 66.
LaTeX Font Info:    Font shape `T1/Merriweather-OsF/regular/n' will be
(Font)              scaled to size 6.5pt on input line 66.
LaTeX Font Info:    Calculating math sizes for size <6.5> on input line
66.
LaTeX Font Info:    Font shape `T1/Merriweather-OsF/m/up' in size <6.5>
not ava
ilable
(Font)              Font shape `T1/Merriweather-OsF/regular/n' tried
instead on
 input line 66.
LaTeX Font Info:    Font shape `T1/Merriweather-OsF/regular/n' will be
(Font)              scaled to size 6.5pt on input line 66.
LaTeX Font Info:    Font shape `T1/Merriweather-OsF/m/up' in size
<5.41643> not
 available
(Font)              Font shape `T1/Merriweather-OsF/regular/n' tried
instead on
 input line 66.
LaTeX Font Info:    Font shape `T1/Merriweather-OsF/regular/n' will be
(Font)              scaled to size 5.41643pt on input line 66.
LaTeX Font Info:    Font shape `T1/Merriweather-OsF/m/up' in size
<4.54997> not
 available
(Font)              Font shape `T1/Merriweather-OsF/regular/n' tried
instead on
 input line 66.
LaTeX Font Info:    Font shape `T1/Merriweather-OsF/regular/n' will be
(Font)              scaled to size 4.54997pt on input line 66.

LaTeX Font Warning: Font shape `OMS/cmsy/m/n' in size <6.5> not available
```

```
(Font)              size <6> substituted on input line 66.


LaTeX Font Warning: Font shape `OMS/cmsy/m/n' in size <5.41643> not
available
(Font)              size <5> substituted on input line 66.


LaTeX Font Warning: Font shape `OMS/cmsy/m/n' in size <4.54997> not
available
(Font)              size <5> substituted on input line 66.

LaTeX Font Info:    External font `cmex10' loaded for size
(Font)              <6.5> on input line 66.
LaTeX Font Info:    External font `cmex10' loaded for size
(Font)              <5.41643> on input line 66.
LaTeX Font Info:    External font `cmex10' loaded for size
(Font)              <4.54997> on input line 66.

LaTeX Font Warning: Font shape `OML/cmm/m/it' in size <6.5> not available
(Font)              size <6> substituted on input line 66.


LaTeX Font Warning: Font shape `OML/cmm/m/it' in size <5.41643> not
available
(Font)              size <5> substituted on input line 66.


LaTeX Font Warning: Font shape `OML/cmm/m/it' in size <4.54997> not
available
(Font)              size <5> substituted on input line 66.

LaTeX Font Info:    Font shape `T1/Merriweather-OsF/m/n' in size
<5.41643> not
available
(Font)              Font shape `T1/Merriweather-OsF/regular/n' tried
instead on
 input line 66.
LaTeX Font Info:    Font shape `T1/Merriweather-OsF/regular/n' will be
(Font)              scaled to size 5.41643pt on input line 66.
LaTeX Font Info:    Font shape `T1/Merriweather-OsF/m/n' in size
<4.54997> not
available
(Font)              Font shape `T1/Merriweather-OsF/regular/n' tried
instead on
 input line 66.
LaTeX Font Info:    Font shape `T1/Merriweather-OsF/regular/n' will be
(Font)              scaled to size 4.54997pt on input line 66.
LaTeX Font Info:    Font shape `T1/Merriweather-OsF/m/it' in size <6.5>
not ava
ilable
(Font)              Font shape `T1/Merriweather-OsF/regular/it' tried
instead o
n input line 66.
```

```
LaTeX Font Info:    Font shape `T1/Merriweather-OsF/regular/it' will be
(Font)              scaled to size 6.5pt on input line 66.
LaTeX Font Info:    Font shape `T1/Merriweather-OsF/m/it' in size
<5.41643> not
 available
(Font)              Font shape `T1/Merriweather-OsF/regular/it' tried
instead o
n input line 66.
LaTeX Font Info:    Font shape `T1/Merriweather-OsF/regular/it' will be
(Font)              scaled to size 5.41643pt on input line 66.
LaTeX Font Info:    Font shape `T1/Merriweather-OsF/m/it' in size
<4.54997> not
 available
(Font)              Font shape `T1/Merriweather-OsF/regular/it' tried
instead o
n input line 66.
LaTeX Font Info:    Font shape `T1/Merriweather-OsF/regular/it' will be
(Font)              scaled to size 4.54997pt on input line 66.
LaTeX Font Info:    Font shape `T1/MerriweatherSans-OsF/m/n' in size
<6.5> not
available
(Font)              Font shape `T1/MerriweatherSans-OsF/regular/n' tried
instea
d on input line 66.
LaTeX Font Info:    Font shape `T1/MerriweatherSans-OsF/regular/n' will
be
(Font)              scaled to size 6.5pt on input line 66.
LaTeX Font Info:    Font shape `T1/MerriweatherSans-OsF/m/n' in size
<5.41643>
not available
(Font)              Font shape `T1/MerriweatherSans-OsF/regular/n' tried
instea
d on input line 66.
LaTeX Font Info:    Font shape `T1/MerriweatherSans-OsF/regular/n' will
be
(Font)              scaled to size 5.41643pt on input line 66.
LaTeX Font Info:    Font shape `T1/MerriweatherSans-OsF/m/n' in size
<4.54997>
not available
(Font)              Font shape `T1/MerriweatherSans-OsF/regular/n' tried
instea
d on input line 66.
LaTeX Font Info:    Font shape `T1/MerriweatherSans-OsF/regular/n' will
be
(Font)              scaled to size 4.54997pt on input line 66.
LaTeX Font Info:    Font shape `TS1/Merriweather-OsF/m/n' in size
<5.41643> not
 available
(Font)              Font shape `TS1/Merriweather-OsF/regular/n' tried
instead o
n input line 66.
LaTeX Font Info:    Font shape `TS1/Merriweather-OsF/regular/n' will be
(Font)              scaled to size 5.41643pt on input line 66.
```

```
Overfull \hbox (54.64pt too wide) in paragraph at lines 66--66
[][][]
 []

LaTeX Font Info:    Font shape `T1/Merriweather-OsF/b/n' in size <10> not
avail
able
(Font)              Font shape `T1/Merriweather-OsF/bold/n' tried instead
on in
put line 66.
LaTeX Font Info:    Font shape `T1/Merriweather-OsF/bold/n' will be
(Font)              scaled to size 10.0pt on input line 66.
LaTeX Font Info:    Font shape `T1/Merriweather-OsF/b/n' in size <8> not
availa
ble
(Font)              Font shape `T1/Merriweather-OsF/bold/n' tried instead
on in
put line 66.
LaTeX Font Info:    Font shape `T1/Merriweather-OsF/bold/n' will be
(Font)              scaled to size 8.0pt on input line 66.

Overfull \hbox (54.64pt too wide) in paragraph at lines 66--66
[][][]
 []


Underfull \vbox (badness 10000) has occurred while \output is active []



pdfTeX warning: pdflatex.exe (file ./Fig1.pdf): PDF inclusion: found PDF
versio
n <1.7>, but at most version <1.5> allowed
<Fig1.pdf, id=68, 782.925pt x 542.025pt>
File: Fig1.pdf Graphic file (type pdf)
<use Fig1.pdf>
Package pdftex.def Info: Fig1.pdf  used on input line 77.
(pdftex.def)             Requested size: 414.99666pt x 287.30496pt.
LaTeX Font Info:    Font shape `T1/Merriweather-OsF/m/n' in size <6> not
availa
ble
(Font)              Font shape `T1/Merriweather-OsF/regular/n' tried
instead on
 input line 79.
LaTeX Font Info:    Font shape `T1/Merriweather-OsF/regular/n' will be
(Font)              scaled to size 6.0pt on input line 79.
LaTeX Font Info:    Font shape `T1/Merriweather-OsF/b/n' in size <6> not
availa
ble
(Font)              Font shape `T1/Merriweather-OsF/bold/n' tried instead
on in
put line 79.
LaTeX Font Info:    Font shape `T1/Merriweather-OsF/bold/n' will be
(Font)              scaled to size 6.0pt on input line 79.
```

```
LaTeX Font Info:    Font shape `T1/lmtt/bx/n' in size <6> not available
(Font)              Font shape `T1/lmtt/b/n' tried instead on input line
79.
LaTeX Font Info:    Font shape `T1/Merriweather-OsF/m/it' in size <6> not
avail
able
(Font)              Font shape `T1/Merriweather-OsF/regular/it' tried
instead o
n input line 79.
LaTeX Font Info:    Font shape `T1/Merriweather-OsF/regular/it' will be
(Font)              scaled to size 6.0pt on input line 79.
LaTeX Font Info:    Font shape `T1/Merriweather-OsF/b/n' in size <8.5>
not avai
lable
(Font)              Font shape `T1/Merriweather-OsF/bold/n' tried instead
on in
put line 85.
LaTeX Font Info:    Font shape `T1/Merriweather-OsF/bold/n' will be
(Font)              scaled to size 8.5pt on input line 85.

Underfull \vbox (badness 10000) has occurred while \output is active []

LaTeX Font Info:    Font shape `T1/Merriweather-OsF/m/n' in size <7.8>
not avai
lable
(Font)              Font shape `T1/Merriweather-OsF/regular/n' tried
instead on
 input line 89.
LaTeX Font Info:    Font shape `T1/Merriweather-OsF/regular/n' will be
(Font)              scaled to size 7.8pt on input line 89.
LaTeX Font Info:    Font shape `T1/Merriweather-OsF/b/n' in size <7.8>
not avai
lable
(Font)              Font shape `T1/Merriweather-OsF/bold/n' tried instead
on in
put line 89.
LaTeX Font Info:    Font shape `T1/Merriweather-OsF/bold/n' will be
(Font)              scaled to size 7.8pt on input line 89.
[1{c:/TeXLive/2020/texmf-var/fonts/map/pdftex/updmap/pdftex.map}


]

pdfTeX warning: pdflatex.exe (file ./Fig2.pdf): PDF inclusion: found PDF
versio
n <1.7>, but at most version <1.5> allowed
<Fig2.pdf, id=95, 341.35529pt x 654.40488pt>
File: Fig2.pdf Graphic file (type pdf)
<use Fig2.pdf>
Package pdftex.def Info: Fig2.pdf  used on input line 98.
(pdftex.def)             Requested size: 235.11394pt x 450.74101pt.
<Fig3.pdf, id=96, 578.16pt x 829.19785pt>
File: Fig3.pdf Graphic file (type pdf)
<use Fig3.pdf>
```

```
Package pdftex.def Info: Fig3.pdf  used on input line 108.
(pdftex.def)             Requested size: 235.11394pt x 337.18976pt.

Underfull \vbox (badness 2469) has occurred while \output is active []


Underfull \vbox (badness 1043) has occurred while \output is active []

LaTeX Font Info:    Font shape `T1/Merriweather-OsF/m/it' in size <7.8>
not ava
ilable
(Font)              Font shape `T1/Merriweather-OsF/regular/it' tried
instead o
n input line 127.
LaTeX Font Info:    Font shape `T1/Merriweather-OsF/regular/it' will be
(Font)              scaled to size 7.8pt on input line 127.
[2 <./Fig1.pdf>]
LaTeX Font Info:    Font shape `T1/Merriweather-OsF/m/up' in size <7.5>
not ava
ilable
(Font)              Font shape `T1/Merriweather-OsF/regular/n' tried
instead on
 input line 132.
LaTeX Font Info:    Font shape `T1/Merriweather-OsF/regular/n' will be
(Font)              scaled to size 7.5pt on input line 132.

Underfull \vbox (badness 4859) has occurred while \output is active []


Underfull \hbox (badness 5133) in paragraph at lines 137--138
[][]\T1/Merriweather-OsF/regular/n/7.5 Long-read-tools.org[][] con-tains
478 to
ols at the time of
 []

[3 <./Fig2.pdf> <./Fig3.pdf

pdfTeX warning: pdflatex.exe (file ./Fig3.pdf): PDF inclusion: multiple
pdfs wi
th page group included in a single page
>]
Underfull \hbox (badness 3058) in paragraph at lines 158--159
[]\T1/Merriweather-OsF/regular/n/7.5 Other bioin-for-matic fields have
ex-pe-ri
-enced a sim-i-lar
 []


Underfull \hbox (badness 4467) in paragraph at lines 160--161
\T1/Merriweather-OsF/regular/n/7.5 Workflow-Frameworks-Platforms Google
Sheets
[[]] list,
 []
```

```
LaTeX Font Info:    Font shape `TS1/Merriweather-OsF/m/n' in size <7.5>
not ava
ilable
(Font)              Font shape `TS1/Merriweather-OsF/regular/n' tried
instead o
n input line 167.
LaTeX Font Info:    Font shape `TS1/Merriweather-OsF/regular/n' will be
(Font)              scaled to size 7.5pt on input line 167.

Underfull \hbox (badness 10000) in paragraph at lines 169--170
[]\T1/Merriweather-OsF/regular/n/7.5 Source code avail-abil-ity:
 []


Underfull \hbox (badness 10000) in paragraph at lines 169--170
[][]\T1/Merriweather-OsF/regular/n/7.5
https://github.com/shaniAmare/long_read_
tools[][]. An
 []


Underfull \hbox (badness 3229) in paragraph at lines 172--173
[]\T1/Merriweather-OsF/regular/n/7.5 Other re-quire-ments: Ac-ces-si-ble
via an
y mod-ern web
 []

[4]
Underfull \hbox (badness 4981) in paragraph at lines 223--227
[]\T1/Merriweather-OsF/regular/n/7.5 Ho SS, Ur-ban AE, Mills RE.  Struc-
tural v
ari-a-tion
 []


Underfull \hbox (badness 6204) in paragraph at lines 223--227
\T1/Merriweather-OsF/regular/n/7.5 in the se-quenc-ing era.  Na-ture Re-
views G
e-net-ics
 []


Underfull \hbox (badness 10000) in paragraph at lines 223--227
\T1/Merriweather-OsF/regular/n/7.5 2020 mar;21(3):171--189.
[][]$\T1/lmtt/m/n/
7.5 https : / / doi . org / 10 . 1038 /
 []


Underfull \hbox (badness 1803) in paragraph at lines 230--234
[]\T1/Merriweather-OsF/regular/n/7.5 Mitsuhashi S, Mat-sumoto N.  Long-
read se-
quenc-ing
 []
```

```
Underfull \hbox (badness 1152) in paragraph at lines 230--234
\T1/Merriweather-OsF/regular/n/7.5 for rare hu-man ge-netic dis-eases.
Jour-na
l of Hu-man
 []


Underfull \hbox (badness 1152) in paragraph at lines 335--339
\T1/Merriweather-OsF/regular/n/7.5 bioRxiv 2017 apr;p. 126656.
[][]$\T1/lmtt/m
/n/7.5 https : / / doi . org / 10 . 1101 /
 []


Underfull \vbox (badness 10000) has occurred while \output is active []


Underfull \hbox (badness 4391) in paragraph at lines 368--372
[]\T1/Merriweather-OsF/regular/n/7.5 Loman NJ, Quin-lan AR.  Pore-tools:
a tool
kit for
 []


Underfull \hbox (badness 5245) in paragraph at lines 368--372
\T1/Merriweather-OsF/regular/n/7.5 an-a-lyz-ing nanopore se-quence data.
Bioin
-for-mat-ics
 []


Underfull \hbox (badness 5756) in paragraph at lines 368--372
\T1/Merriweather-OsF/regular/n/7.5 2014 08;30(23):3399--3401.
[][]$\T1/lmtt/m/
n/7.5 https : / / doi . org / 10 . 1093 /
 []


Underfull \hbox (badness 1221) in paragraph at lines 386--391
[]\T1/Merriweather-OsF/regular/n/7.5 Li H, Durbin R.  Fast and ac-cu-rate
long-
read align-
 []


Underfull \hbox (badness 1565) in paragraph at lines 386--391
\T1/Merriweather-OsF/regular/n/7.5 ics 2010 jan;26(5):589--595.
[][]$\T1/lmtt/
m/n/7.5 https : / / doi . org / 10 . 1093 /
 []
```

```
Underfull \hbox (badness 10000) in paragraph at lines 424--428
[]\T1/Merriweather-OsF/regular/n/7.5 Elliott TA.  Re-peat_Resources -
Google
 []


Underfull \hbox (badness 10000) in paragraph at lines 424--428
\T1/Merriweather-OsF/regular/n/7.5 Sheets. [][]$\T1/lmtt/m/n/7.5 https :
/ / do
cs . google . com / spreadsheets / d /
 []


Underfull \hbox (badness 10000) in paragraph at lines 424--428
\T1/lmtt/m/n/7.5 1UBK70zExiL0gFVaIAILiGhflCGXAq _ SF _ lymaxTE1pY / edit
#
 []


Underfull \hbox (badness 1173) in paragraph at lines 430--435
[]\T1/Merriweather-OsF/regular/n/7.5 Davis S.  seandavi/awesome-single-
cell: Co
mmunity-
 []


Underfull \hbox (badness 1189) in paragraph at lines 430--435
\T1/Merriweather-OsF/regular/n/7.5 single-cell, in-clud-ing RNA-seq,
ATAC-seq,
etc. [][]$\T1/lmtt/m/n/7.5 https :
 []


Underfull \hbox (badness 6428) in paragraph at lines 438--443
[]\T1/Merriweather-OsF/regular/n/7.5 Zappia L, Phip-son B, Osh-lack A.
Ex-plor
-ing the
 []


Underfull \hbox (badness 5260) in paragraph at lines 438--443
\T1/Merriweather-OsF/regular/n/7.5 tools database.  PLOS Com-pu-ta-tional
Bi-ol
-ogy 2018
 []


Underfull \hbox (badness 7814) in paragraph at lines 438--443
\T1/Merriweather-OsF/regular/n/7.5 jun;14(6):e1006245.
[][]$\T1/lmtt/m/n/7.5 h
ttps : / / doi . org / 10 . 1371 / journal .
 []
```

```
Underfull \hbox (badness 1270) in paragraph at lines 446--450
[]\T1/Merriweather-OsF/regular/n/7.5 Molecular Mi-cro-bi-ol-ogy and In-
fec-tion
 Unit, Uni-ver-sity
 []


Underfull \hbox (badness 2799) in paragraph at lines 446--450
\T1/Merriweather-OsF/regular/n/7.5 of Lis-bon.  B-UMMI/long-read-catalog:
cat-a
-log for
 []


Underfull \hbox (badness 10000) in paragraph at lines 452--457
[]\T1/Merriweather-OsF/regular/n/7.5 Vilella A.  Bioinformatics-Workflow-
Framew
orks-
 []


Underfull \hbox (badness 10000) in paragraph at lines 452--457
\T1/Merriweather-OsF/regular/n/7.5 Platforms.v6.6.6 - Google Sheets.
[][]$\T1/l
mtt/m/n/7.5 https :
 []


Underfull \hbox (badness 10000) in paragraph at lines 452--457
\T1/lmtt/m/n/7.5 / / docs . google . com / spreadsheets / d / 1plkAsT _
 []


Underfull \hbox (badness 10000) in paragraph at lines 452--457
\T1/lmtt/m/n/7.5 S3CzSeb7ivxyjRnHyrK3JclUCXeUMf _ azraY / edit # gid =
 []


AED: lastpage setting LastPage
[5]
Package atveryend Info: Empty hook `BeforeClearDocument' on input line
473.
Package atveryend Info: Empty hook `AfterLastShipout' on input line 473.
(./main.aux)
Package atveryend Info: Executing hook `AtVeryEndDocument' on input line
473.
Package atveryend Info: Executing hook `AtEndAfterFileList' on input line
473.
Package rerunfilecheck Info: File `main.out' has not changed.
(rerunfilecheck)             Checksum:
A645F4227C24E685714B64DC03327060;842.

LaTeX Font Warning: Size substitutions with differences
(Font)              up to 1.0pt have occurred.
```

```
LaTeX Font Warning: Some font shapes were not available, defaults
substituted.

 )
Here is how much of TeX's memory you used:
 18478 strings out of 480681
 337855 string characters out of 5908536
 674929 words of memory out of 5000000
 33819 multiletter control sequences out of 15000+600000
 596455 words of font info for 204 fonts, out of 8000000 for 9000
 1141 hyphenation exceptions out of 8191
 65i,12n,110p,965b,857s stack positions out of
5000i,500n,10000p,200000b,80000s
{c:/TeXLive/2020/texmf-dist/fonts/enc/dvips/lm/lm-
ec.enc}{c:/TeXLive/2020/tex
mf-
dist/fonts/enc/dvips/merriweather/mwth_clyrx2.enc}{c:/TeXLive/2020/texmf-
dis
t/fonts/enc/dvips/merriweather/mwth_l3riwr.enc}{c:/TeXLive/2020/texmf-
dist/font
s/enc/dvips/cm-super/cm-super-t1.enc}<c:/TeXLive/2020/texmf-
dist/fonts/truetype
/sorkin/merriweather/Merriweather-BoldIt.ttf><c:/TeXLive/2020/texmf-
dist/fonts/
type1/sorkin/merriweather/Merriweather-Bold.pfb><c:/TeXLive/2020/texmf-
dist/fon
ts/type1/sorkin/merriweather/Merriweather-
Italic.pfb><c:/TeXLive/2020/texmf-dis
t/fonts/type1/sorkin/merriweather/Merriweather-
Regular.pfb><c:/TeXLive/2020/tex
mf-dist/fonts/type1/public/lm/lmtk10.pfb><c:/TeXLive/2020/texmf-
dist/fonts/type
1/public/lm/lmtt8.pfb><c:/TeXLive/2020/texmf-dist/fonts/type1/public/cm-
super/s
frm1200.pfb>
Output written on main.pdf (5 pages, 1070727 bytes).
PDF statistics:
 2230 PDF objects out of 2487 (max. 8388607)
 1243 compressed objects within 13 object streams
 57 named destinations out of 1000 (max. 500000)
 42104 words of extra memory for PDF output out of 42996 (max. 10000000)
```

reference pdf
Click here to access/download;Manuscript;reference_pdf.pdf

*GigaScience*, 2021, 1–5

doi: xx.xxxx/xxxx
Manuscript in Preparation
Technical Note

TECHNICAL NOTE

# long-read-tools.org: an interactive catalogue of analysis methods for long-read sequencing data

Shanika L. Amarasinghe [1,2*], Matthew E. Ritchie [1,2,3] and Quentin Gouil [1,2*]

[1]Epigenetics and Development Division, The Walter and Eliza Hall Institute of Medical Research, 1G Royal Parade, Parkville, Victoria 3052, Australia and [2]Department of Medical Biology, The University of Melbourne, Parkville, Victoria 3010, Australia and [3]School of Mathematics and Statistics, The University of Melbourne, Parkville, Victoria 3010, Australia

*amarasinghe.s@wehi.edu.au; gouil.q@wehi.edu.au

## Abstract

**Background** The data produced by long-read third-generation sequencers have unique characteristics compared to short-read sequencing data, often requiring tailored analysis tools for tasks ranging from quality control to downstream processing. The rapid growth in software that address these challenges for different genomics applications are difficult to keep track of, which makes it hard for users to choose the most appropriate tool for their analysis goal, and for developers to identify areas of need and existing solutions to benchmark against.
**Findings** We describe the implementation of long-read-tools.org, an open-source database that organises the rapidly expanding collection of long-read data analysis tools and allows its exploration through interactive browsing and filtering. The current database release contains 478 tools across 32 categories. Most tools are developed in Python and the most frequent analysis tasks include basecalling, *de novo* assembly, error-correction, quality checking/filtering, and isoform detection, while long-read single-cell data analysis and transcriptomics are areas with the fewest tools available.
**Conclusion** Continued growth in the application of long-read sequencing in genomics research positions the long-read-tools.org database as an essential resource that allows researchers to keep abreast of both established and emerging software to help guide the selection of the most relevant tool for their analysis needs.

**Key words**: database; long-read sequencing; data analysis; nanopore; PacBio

## Background

Long-read sequencing technologies facilitate versatile exploration of genomes owing to their ability to generate reads spanning several thousand base pairs [1]. Long reads can be *de novo* assembled or mapped to a reference to identify complicated structural variants and novel or complete transcripts that may otherwise be difficult to distinguish with short-read sequencing [2, 3, 4]. Improvements in throughput, error and cost reduction as well as increased interest in tool development for downstream data analyses [5] all contribute to the broadening adoption of long-read data across research fields.

To keep up with the rapid growth in software for long-read analysis, we collated and categorised existing long-read analysis tools at long-read-tools.org. This database enables easy navigation of the available software, allowing users to filter by specific tasks to identify methods that suit their analysis objectives.

## Findings

**Compiled on:** January 12, 2021.
Draft manuscript prepared by the author.

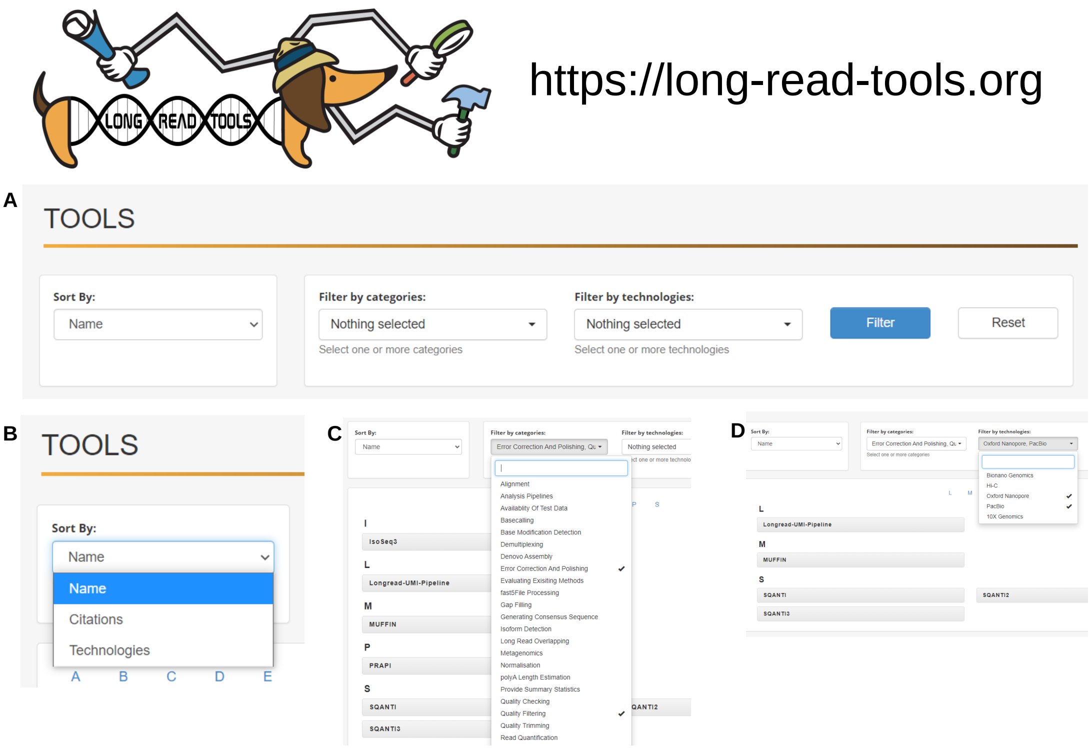


**Figure 1. Example use of the `Tools` tab from long-read-tools.org** A. The custom toolbar for the page. B. Drop-down *Sort By* menu. C. Drop-down *Filter by categories* menu which allows users to select multiple options by clicking on an item or typing the word in the text box. D. Drop-down *Filter by technologies* menu which allows users to select multiple options by clicking on an item or typing the word in the text box. When multiple categories or technologies are selected, the website returns the intersection, not the union, i.e. a tool has to satisfy all the requirements to be reported.

## Data Collection, Database Design & Implementation

The long-read-tools.org database is specifically designed to catalogue analysis tools for long-reads generated from genuine (PacBio and ONT) and synthetic (e.g. Hi-C, 10x, Bionano Genomics) long-read technologies. Up-to-date data is collected from various sources including publications, preprints, social media posts, mining public (GitHub, PyPI, Anaconda, CRAN, Bioconductor) and private repositories and via the tool submissions form accessible from the `Submit` tab.

The data collected in the form of a `.csv` file is processed within the R environment [6]. In this `.csv` file, each tool is categorised with a `TRUE` or `FALSE` value based on its functionality and technology(ies) in focus. Available details of the tools such as the description, publication status, tool licence and programming language are retrieved and stored. Furthermore, the total number of citations for each tool is retrieved via rcrossref (v1.0.0) [7] and stored, while the number of citations from the past year is obtained through the citecorp R package (v0.3.0) [8] from the COCI database [9]. Both citation metrics may serve as an indication of a tool's popularity. Information on arXiv preprints is retrieved through the arXiv package (v0.5.19) [10]. Multiple `JSON` files are generated during the processing step to populate the website. If publicly available, a tool's source code is checked to assess the current status of its code base (e.g. actively maintained or deprecated).

Several analysis-style plots are created to be displayed on the database as well. The original `.csv` input is processed to extract details such as the number of tools across time, the distribution of tools across categories, publication status and the main programming platforms used in tools development to summarise the contents of the database. The plots are created in the R environment using several main packages such as `ggplot2` (v3.3.2) [11] and `plotly` (v4.9.2) [12].

## Database Usage

The long-read-tools.org website consists of several tabs, the first of which is the landing page (`Home`) that provides a summary of the database. The second `Table` tab contains the primary table with information that can be filtered using the search bar on the right. This tab can be used to view and download the required details of the complete database or a set of tools of interest.

Next is the `Tools` tab (Figure 1A), which is the most important section of the database. This tab contains individual details on each software package (e.g. name, description, publication information, number of citations, location of the source code, etc.) and is intuitive to navigate.

If a user requires to sort through software tools by name, number of citations, or technology, one of these options can be selected from the drop-down menu in the left hand corner, which will re-order the tools according to the selected parameter (Figure 1B). This sort function can be used on its own or together with the filtering drop down menus in the middle and the right hand side of the page.

The filtering options allow the user to select multiple items from each of the filtering criteria (i.e. categories and technology) and will report the intersection. The union would be obtained by separate individual searches. For example, if the user wants to identify tools that can do both "error correction and polishing" and "quality filtering", either typing them in the keyword box or clicking on the category item and pressing the filter option will show the filtered subset of tools (Figure 1C). Only seven tools match these criteria; all are pipelines rather than software dedicated to a unique task, as expected for the intersection of error correction and quality filtering functionalities. Of note, *SQANTI1* and 2 are superseded by *SQANTI3* [13], which is indicated when accessing the tools' details. The user

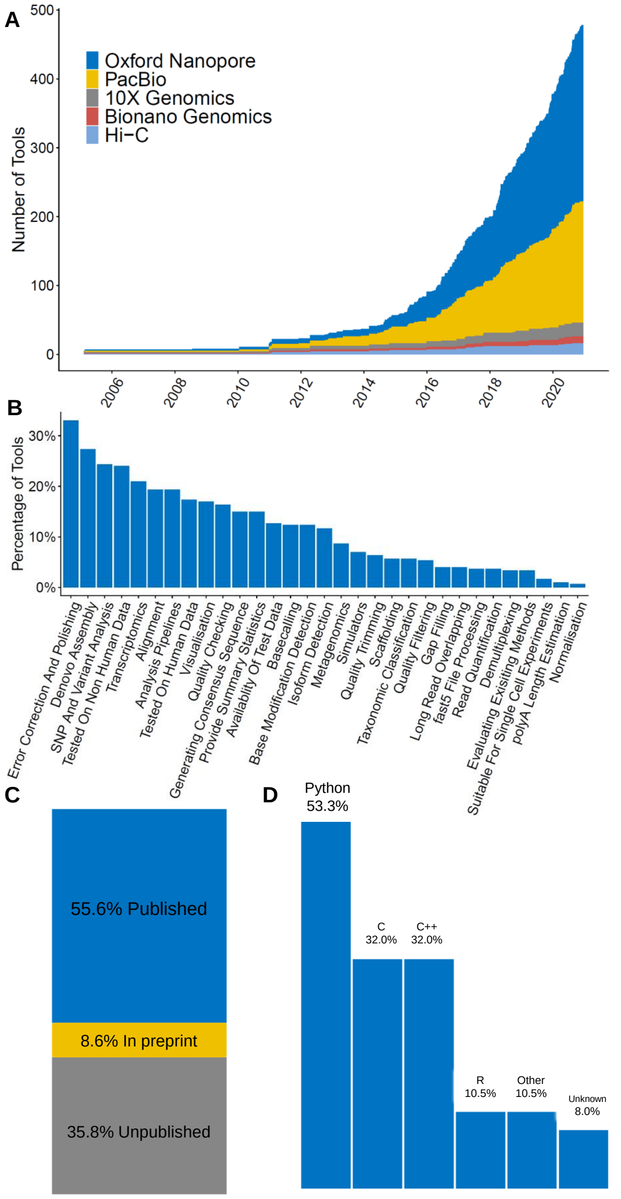


**Figure 2. Summary statistics from long-read-tools.org** A. The number of tools released over time stratified by the long-read technologies they serve. B. The data analysis categories covered by the catalogued tools (ordered from most to least frequent). C. Publication status of the catalogued tools. D. The programming platforms used by the catalogued tools (ordered from most to least frequent). All languages making up at least 10% of a tool's code are reported. These summary plots are available from the `Statistics` tab of the database website and can be easily exported for reuse.

can subset these findings further based on their preferred technology. Selecting Oxford Nanopore and PacBio returns the tools that are confirmed to work with both, thus removing *PRAPI* and *IsoSeq3* that are specialised for PacBio data (Figure 1D). However we note that a tool that has only been tested on one technology, and is thus annotated only with one, may well be applicable to another given the similarities in data characteristics between long-read platforms.

The `Statistics` tab contains summary plots obtained from an analysis of the information contained within the database (Figure 2, e.g. growth in tool development over time, the distribution of tools across analysis tasks, publication status, summary of the programming languages they use, etc.).

The `Submit` tab is where the user can provide new information to the database if they have a tool to submit or modify.

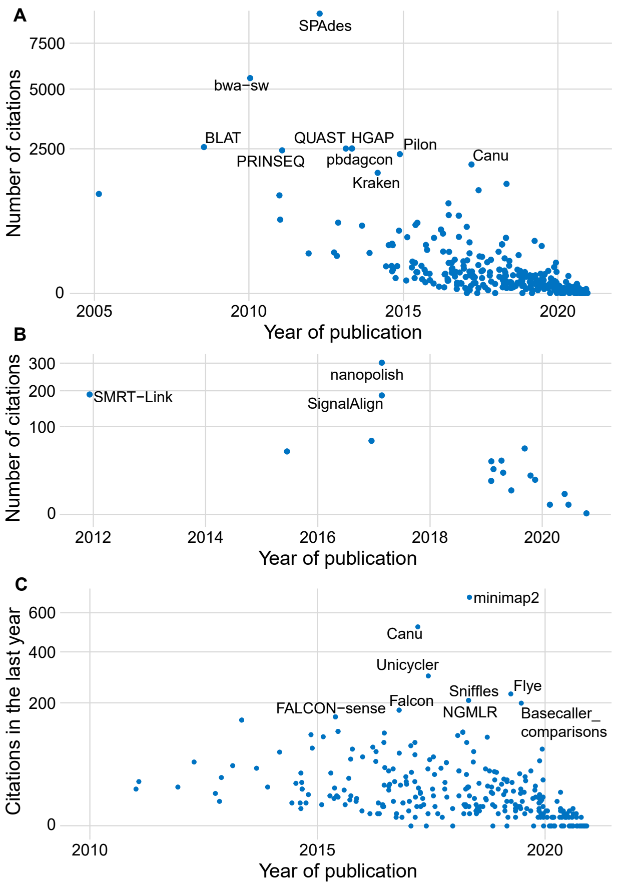


**Figure 3. Popularity of the tools from long-read-tools.org based on publication citations** A. Across the entire database. B. For *base modification detection*. C. Across the entire database for citations in the last year. Each panel shows the year of publication on the x-axis and the square root of the number of citations on the y-axis. If the input set of tools is larger than 50, the 10 most cited tools are labeled, otherwise the 3 most cited tools are labeled

The final tabs (`Updates`, `FAQs` and `Contact Us`) provide a summary of the social media activity of *@long_read_tools* (Twitter), answers to frequently asked questions and a form to contact the database creators to ask general questions, respectively.

## Database Statistics

Long-read-tools.org contains 478 tools at the time of manuscript submission (Figure 2A). These include 229, 155, 20, 15 and 10 tools that can handle ONT, PacBio, 10x, Hi-C, Bionano Genomics data, respectively.

Tools began to appear in publications from the year 2005, although these were not targeted to long-read sequence analysis at that time. Tools focused on short-read alignment such as *Gmap* [14], *Soap-denovo* [15] and *STAR* [16] have made alterations to their algorithms in order to support error-prone long-read sequence alignments. Nevertheless, short-read aligners have also been tested for their ability to work with long reads [17].

Tools specifically focused on long-read sequence analysis became available from 2012, following the commercial release of the PacBio RS sequencer in 2011 (see for example PBcR [18, 19] and LSC [20]). The ONT MinION was commercially released in 2014, and Poretools was published in the same year [21].

Available tools are categorised into 32 different functions (Figure 2B). Of these, "error correction and polishing" and "*de novo* assembly" are the most common. On the other hand,

"polyA length estimation", "suitable for single cell experiments" and "normalisation" have the lowest number of tools, which highlights areas for further research and tool development.

It is also exciting to see the majority of the tools have been published in either a peer-reviewed journal or on a preprint server (Figure 2C). Moreover, tools written in Python outnumber tools implemented in other programming languages (Figure 2D).

In terms of the number of citations, *SPAdes* [22] and *bwa-sw* [23] lead the pack (Figure 3A). However, it should be noted that these tools existed before long-read technologies were popular, and most of these citations will therefore not reflect their popularity in long-read data analysis. The number of citations provides a more accurate indicator of usage for the tools that are unique to long-read analyses (e.g. *nanopolish* [24], *SMRT-Link* [25] and *SignalAlign* [26] in "base modification detection" (Figure 3B). To better capture the popularity of tools in a rapidly-moving field, we also report the number of citations in the past year (Figure 3C). For instance it can be observed that the *Flye* assembler [27] has been highly cited in the past 12 months despite its recent publication date (April 2019).

## Summary and Future Work

Long-read-tools.org is an an up-to-date, user-friendly catalogue that allows efficient searching of software by analysis category. It provides a comprehensive resource for new users to quickly and easily identify the relevant tools for their long-read data type and desired application. Our database illustrates the main areas of focus for existing tools, as well as the lack of software available in other areas (e.g. transcriptomics).

Other bioinformatic fields have experienced a similar growth in the number of available tools, prompting efforts to collate and organise them. These efforts vary from simple spreadsheets that list resources for the analysis of genomic repeats [28], through clickable lists of single-cell data analysis tools hosted on GitHub [29], all the way to dedicated websites offering search functions and statistics, such as scRNA-tools [30] that indexes tools for single-cell transcriptomics.

For long-read data, the long-read-catalog GitHub page [31] collects 40 tools for the analysis of ONT and PacBio data but it has not been updated in the last year. The Bioinformatics-Workflow-Frameworks-Platforms Google Sheets [32] list, among many other things, 84 tools relating to ONT data and 82 applicable to PacBio data. long-read-tools.org is both more comprehensive and easier to navigate than these databases.

We intend to keep increasing the breadth and depth of long-read-tools.org, but this should not come at the cost of making the database overwhelming to browse. Tutorials such as the 'Long-read, long reach Bioinformatics Tutorials' website [33] are helpful in understanding how multiple tools fit into an analysis pipeline. Therefore we are focusing current efforts on facilitating the identification of best practices, validated workflows, and each tool's relative strengths and weaknesses. Four additional entries are already available at tool submission and will be progressively populated: Underlying Algorithms, Underlying Assumptions, Strengths and Weaknesses, and Overall Performance. Furthermore a `Tutorials` tab highlighting common validated workflows and a `Benchmarks` tab featuring benchmarking studies and their results are in development.

## Availability of Source Code and Requirements

- Project name: Long-read-tools.org database
- Project home page: long-read-tools.org
- Source code availability: https://github.com/shaniAmare/long_read_tools. An archival copy of the code is available via the GigaScience database GigaDB [34].
- Operating system(s): Platform independent
- Programming language(s): R/JavaScript/html
- Other requirements: Accessible via any modern web browser
- License: MIT
- SciCrunch RRID:SCR_019116
- Biotools ID: biotools:long-read-tools

long-read-tools.org is a community effort, and we encourage researchers to contribute relevant tools, benchmarks, tutorials and improvements to the database via the `Submit` tab.

## Declarations

### List of Abbreviations

ONT: Oxford Nanopore Technologies
PacBio: Pacific Biosciences
10x: 10x Genomics

### Competing Interests

The authors declare that they have no competing interests.


### Funding

This work was supported by funding from the Chan Zuckerberg Initiative DAF, an advised fund of Silicon Valley Community Foundation (grant number 2019-002443 to MER), a fellowship from the Australian National Health and Medical Research Council (NHMRC, grant number GNT1104924 to MER), Victorian State Government Operational Infrastructure Support and Australian Government NHMRC IRIISS.


### Authors' Contributions

SLA structured the database, developed, implemented and populated it and wrote the manuscript. MER guided the research and wrote the manuscript. QG structured the database, populated and validated entries, and wrote the manuscript. All authors read and approved the final manuscript.


## Acknowledgements

We thank Dr Luke Zappia, the main developer of the scRNA-tools.org database that this work builds upon, for his support in the initial stages of this project, Ms Xueyi Dong and Mr Shian Su for providing constructive feedback on the database, Mr Sujith S. Waduge, Mr Isuru Palliyaguru and Mr Jithendra Sirimanne for their guidance in making the JavaScript underlying the database visualisation more reproducible and user-friendly and Ms Tamara Beck and Ms Ellen Conti for creating the database logo.


## References


1. Logsdon GA, Vollger MR, Eichler EE. Long-read human genome sequencing and its applications. Nature Reviews Genetics 2020 jun;p. 1–18. https://doi.org/10.1038/s41576-020-0236-x.

2. Sakamoto Y, Sereewattanawoot S, Suzuki A. A new era of long-read sequencing for cancer genomics. Journal of Human Genetics 2020 jan;65(1):3–10. https://doi.org/10.1038/s10038-019-0658-5.
3. Ho SS, Urban AE, Mills RE. Structural variation in the sequencing era. Nature Reviews Genetics 2020 mar;21(3):171–189. https://doi.org/10.1038/s41576-019-0180-9.
4. Mitsuhashi S, Matsumoto N. Long-read sequencing for rare human genetic diseases. Journal of Human Genetics 2020 jan;65(1):11–19. https://doi.org/10.1038/s10038-019-0671-8.
5. Pollard MO, Gurdasani D, Mentzer AJ, Porter T, Sandhu MS. Long reads: their purpose and place. Human molecular genetics 2018 aug;27(R2):R234–R241. https://doi.org/10.1093/hmg/ddy177.
6. R Development Core Team. R: A Language and Environment for Statistical Computing. Vienna, Austria: R Foundation for Statistical Computing; 2012, http://www.R-project.org, ISBN 3-900051-07-0.
7. Chamberlain S, Zhu H, Jahn N, Boettiger C, Ram K. rcrossref: Client for Various 'CrossRef' 'APIs'; 2020, https://CRAN.R-project.org/package=rcrossref, r package version 1.0.0.
8. Chamberlain S. citecorp: Client for the Open Citations Corpus; 2020, https://CRAN.R-project.org/package=citecorp, r package version 0.3.0.
9. Heibi I, Peroni S, Shotton D. Software review: COCI, the OpenCitations Index of Crossref open DOI-to-DOI citations. Scientometrics 2019 Nov;121(2):1213–1228. http://link.springer.com/10.1007/s11192-019-03217-6.
10. Ram K, Broman K. aRxiv: Interface to the arXiv API; 2019, https://CRAN.R-project.org/package=aRxiv, r package version 0.5.19.
11. Wickham H. ggplot2: Elegant Graphics for Data Analysis. Springer-Verlag New York; 2016. https://ggplot2.tidyverse.org.
12. Sievert C. Interactive Web-Based Data Visualization with R, plotly, and shiny. Chapman and Hall/CRC; 2020. https://plotly-r.com.
13. Tardaguila M, de la Fuente L, Marti C, Pereira C, Pardo-Palacios FJ, del Risco H, et al. SQANTI: extensive characterization of long-read transcript sequences for quality control in full-length transcriptome identification and quantification. Genome Research 2018 Mar;28(3):396–411. http://genome.cshlp.org/lookup/doi/10.1101/gr.222976.117.
14. Wu TD, Watanabe CK. GMAP: a genomic mapping and alignment program for mRNA and EST sequences. Bioinformatics 2005 may;21(9):1859–1875. https://doi.org/10.1093/bioinformatics/bti310.
15. Luo R, Liu B, Xie Y, Li Z, Huang W, Yuan J, et al. SOAPdenovo2: an empirically improved memory-efficient short-read de novo assembler. GigaScience 2012 dec;1(1):18. https://doi.org/10.1186/2047-217X-1-18.
16. Dobin A, Davis CA, Schlesinger F, Drenkow J, Zaleski C, Jha S, et al. STAR: ultrafast universal RNA-seq aligner. Bioinformatics 2013 jan;29(1):15–21. https://doi.org/10.1093/bioinformatics/bts635.
17. Krizanovic K, Echchiki A, Roux J, Sikic M. Evaluation of tools for long read RNA-seq splice-aware alignment. bioRxiv 2017 apr;p. 126656. https://doi.org/10.1101/126656.
18. Koren S, Schatz MC, Walenz BP, Martin J, Howard JT, Ganapathy G, et al. Hybrid error correction and de novo assembly of single-molecule sequencing reads. Nature Biotechnology 2012 jul;30(7):693–700. https://www.nature.com/articles/nbt.2280.
19. Berlin K, Koren S, Chin CS, Drake JP, Landolin JM, Phillippy AM. Assembling large genomes with single-molecule sequencing and locality-sensitive hashing. Nature Biotechnology 2015 jun;33(6):623–630. https://www.nature.com/articles/nbt.3238.
20. Au KF, Underwood JG, Lee L, Wong WH. Improving PacBio Long Read Accuracy by Short Read Alignment. PLoS ONE 2012 oct;7(10). https://doi.org/10.1371/journal.pone.0046679.
21. Loman NJ, Quinlan AR. Poretools: a toolkit for analyzing nanopore sequence data. Bioinformatics 2014 08;30(23):3399–3401. https://doi.org/10.1093/bioinformatics/btu555.
22. Bankevich A, Nurk S, Antipov D, Gurevich AA, Dvorkin M, Kulikov AS, et al. SPAdes: A New Genome Assembly Algorithm and Its Applications to Single-Cell Sequencing. Journal of Computational Biology 2012 may;19(5):455–477. https://doi.org/10.1089/cmb.2012.0021.
23. Li H, Durbin R. Fast and accurate long-read alignment with Burrows-Wheeler transform. Bioinformatics 2010 jan;26(5):589–595. https://doi.org/10.1093/bioinformatics/btp698.
24. Loman NJ, Quick J, Simpson JT. A complete bacterial genome assembled de novo using only nanopore sequencing data. Nature Methods 2015 jul;12(8):733–735. https://doi.org/10.1038/nmeth.3444.
25. Ardui S, Ameur A, Vermeesch JR, Hestand MS. Single molecule real-time (SMRT) sequencing comes of age: Applications and utilities for medical diagnostics. Nucleic Acids Research 2018;46(5):2159–2168. https://doi.org/10.1093/nar/gky066.
26. Rand AC, Jain M, Eizenga JM, Musselman-Brown A, Olsen HE, Akeson M, et al. Mapping DNA methylation with high-throughput nanopore sequencing. Nature Methods 2017 feb;14(4):411–413. https://doi.org/10.1038/nmeth.4189.
27. Kolmogorov M, Yuan J, Lin Y, Pevzner PA. Assembly of long, error-prone reads using repeat graphs. Nature Biotechnology 2019 May;37(5):540–546. http://www.nature.com/articles/s41587-019-0072-8.
28. Elliott TA. Repeat_Resources - Google Sheets. https://docs.google.com/spreadsheets/d/1UBK70zExiL0gFVaIAILiGhflCGXAq_SF_lymaxTE1pY/edit#gid=1266138738, accessed on 26/08/2020.
29. Davis S. seandavi/awesome-single-cell: Community-curated list of software packages and data resources for single-cell, including RNA-seq, ATAC-seq, etc. https://github.com/seandavi/awesome-single-cell, accessed on 26/08/2020.
30. Zappia L, Phipson B, Oshlack A. Exploring the single-cell RNA-seq analysis landscape with the scRNA-tools database. PLOS Computational Biology 2018 jun;14(6):e1006245. https://doi.org/10.1371/journal.pcbi.1006245.
31. Molecular Microbiology and Infection Unit, University of Lisbon. B-UMMI/long-read-catalog: catalog for long-read sequencing tools. https://github.com/B-UMMI/long-read-catalog, accessed on 26/08/2020.
32. Vilella A. Bioinformatics-Workflow-Frameworks-Platforms.v6.6.6 - Google Sheets. https://docs.google.com/spreadsheets/d/1plkAsT_S3CzSeb7ivxyjRnHyrK3JclUCXeUMf_azraY/edit#gid=471877065, accessed on 26/08/2020.
33. Kahlke T. Long-read, long read bioinformatics tutorials. https://timkahlke.github.io/LongRead_tutorials/, accessed on 26/08/2020.
34. Amarasinghe SL, Ritchie ME, Gouil Q. long-read-tools.org: an interactive catalogue of analysis methods for long-read sequencing data. GigaScience Database 2021. http://dx.doi.org/10.5524/100853.
