# Supplementary material for: long-read-tools.org: an interactive catalogue of analysis methods for long-read sequencing data
Source: Gigascience. 2021 Feb 16;10(2):giab003. doi: 10.1093/gigascience/giab003 (PMC7931822; doi:10.1093/gigascience/giab003)
Supplement: giab003_Response_to_Reviewer_Comments_Original_Submission [file giab003_response_to_reviewer_comments_original_submission.pdf]

Author's Response To Reviewer Comments

Close

Response to editor

Dear Dr Zauner,

We are pleased to submit a revised version of our manuscript “long-read-tools.org: an interactive catalogue of analysis methods for long-read sequencing data”. We have addressed all the reviewers’ comments and made a few additional improvements to the manuscript and database, as outlined in the response to reviewers. The changes to the manuscript pdf are marked in red.
We hope that you find the revised work suitable for publication in Gigascience.

Kind regards,

Quentin Gouil
Senior postdoctoral Researcher
Epigenetics and Development Division
Walter and Eliza Hall Institute

Response to reviewers

We thank the reviewers for their helpful and encouraging comments. In addition to our in-line response to each point, here is a summary of the changes to the manuscript and database:
- we registered long-read-tools.org on bio.tools (biotools:long-read-tools) and SciCrunch (RRID:SCR_019116)
- the “Benchmarks” and “Tutorials” tabs are now live and we hope they will make long-read-tools even more useful and easy to navigate.
- Programming language and license extraction from GitHub repos are now automated, avoiding mistakes and ameliorating formatting consistency
- The database now reports recent citations (from the past year) as well as total citations, making it easier to track a tool’s popularity. We have updated figure 3 with this data.
- We audited all the entries in the database to update DOIs for preprinted work that is now published, check the validity of the links to the code, and annotate deprecated tools.
- Having added a few more tools, the database contains 478 tools as of Dec 20th 2020.
- All figures and statistics have been updated to reflect the state of the database as of Dec 20th 2020.

Reviewer #1:
1) When on the "Tools" tab, if I click on a tool, then on one of its categories, I'm redirected to the "Tools" tab, but the chosen category is not taken into account.
The complete list of tools is thus displayed, and forces the user to re-enter the desired category.
I believe being able to access the tools related to a given category when clicking it from the description of a tool would improve user-friendliness.

We have changed this behaviour so that clicking on a Category within a tool now displays all the tools that fit in the Category, rather than all the tools without filtering. Thank you for suggesting this improvement.

2) Platforms sometimes appear to be wrong.
For instance, CONSENT, which I developed, is listed as "Python", when most of its code is written in C++.
Actually, the GitHub repository contains a Python script in its "bin" folder, but GitHub does report that most of the code is written in C++.

I'm not exactly sure how the platform is inferred (By analyzing the bin folder? The language distribution indicated on GitHub? Manually?), and if other tools are affected, but if it is automatically inferred, I believe it should be verified to keep the database as precise as possible.

Assigning a Language to a tool was until now a manual process during tool submission. This process was prone to errors, as exemplified by the misclassification of CONSENT. In other cases only one language was reported when two or more contributed significantly. Thank you for bringing our attention to this problem. Upon submission of a new tool, if a GitHub repository is provided we now automatically fetch a repository's language distribution via the GitHub API, reporting all languages that amount to at least 10% of the code (excluding code like TeX or Makefiles). We then manually inspect and resolve any differences between the submission and the auto-retrieved data. We now also use the same process to retrieve a tool's license.
Applying this process to the existing database entries allowed us to correct or enrich the language annotations for 138 tools in addition to CONSENT, and we have updated Figure 2D accordingly.

3) The authors state that "For instance, it is clear that all the resulting tools from the above use case can work with PacBio data while only a subset work with ONT data."
However, it does not appear to be entirely clear why the tools would work on PacBio but not on Nanopore.
How is the distinction made exactly? Is it according to the experiments performed in the paper related to the tool, e.g. if it is tested only on PacBio data?
The PacBio and Nanopore reads share a common ground (long sequences, high error-rates), and I don't exactly understand why a given tool, especially for error correction which is underlined in the example, would work on PacBio but not on ONT.
I do understand that some tools were only evaluated on PacBio and perform better on PacBio data, e.g. because of the lower error-rate of PacBio reads compared to ONT, but I don't see why they would not work at all on ONT data.

We have clarified this paragraph. The statement only referred to the specific example of filtering shown in Fig1 C and D, where of the 5 pipelines that could do error correction and quality filtering, 2 were designed specifically for PacBio and not ONT. We have also added a caveat that in general, a tool's annotation with a single technology does not preclude its working with another.
The paragraph now reads:
"For example, if the user wants to identify tools that can do both "error correction and polishing" and "quality filtering", either typing them in the keyword box or clicking on the category item and pressing the filter option will show the filtered subset of tools (Figure 1C). Only seven tools match these criteria; all are pipelines rather than software dedicated to a unique task, as expected for the intersection of error correction and quality filtering functionalities. Of note, SQANTI1 and 2 are superseded by SQANTI3 [13], which is indicated when accessing the tools' details. The user can subset these findings further based on their preferred technology. Selecting Oxford Nanopore and PacBio returns the tools that are confirmed to work with both, thus removing PRAPI and IsoSeq3 that are specialised for PacBio data (Figure 1D). However
we note that a tool that has only been tested on one technology, and is thus annotated only with one, may well be applicable to another given the similarities in data characteristics between long-read platforms."

4) The authors mention that "Tools specifically focused on long-read sequence analysis became available around 2013, coinciding with the commercial release of PacBio Biosciences and Oxford Nanopore Technologies [15] sequencing platforms."
However, tools specifically designed for long-reads actually stated to emerge in 2012, see for instance error-correction tools PBcR (http://doi.org/10.1038/nbt.2280) and LSC (http://doi.org/10.1371/journal.pone.0046679).

We have corrected this inaccurate statement, thank you for bringing it to our attention. The text now reads:
"Tools specifically focused on long-read sequence analysis became available from 2012, following the commercial release of the PacBio RS sequencer in 2011 (see for example PBcR [18, 19] and LSC [20]). The ONT MinION was commercially released in 2014, and Poretools was published in the same year [21]."

Typos / Bibliography remarks :

1) "Moreover, the number of tools written in Python outnumber tools implemented in other programming languages (Figure 2D)."
I believe this sentence should be rewritten, e.g. simply "Moreover, tools written in Python outnumber tools implemented in other programming languages".

This has been corrected.

2) "long-read-tools.orgis both more comprehensive and easier to navigate than these databases."
A space is missing between "long-read-tools.org" and "is"

Fixed.

3) Citation 6 (R Development Core Team. R: A Language and Environment for Statistical Computing. Vienna, Austria: R Foundation for Statistical Computing; 2012, http://www.R-project.org, ISBN 3-900051-07-0, http://www.R-project.org.)
The link to the R project is reported two times. I believe one of the links could be omitted.

Fixed.

4) Citation 19 (Loman NJ, Quick J, Simpson JT. A complete bacterial genome assembled de novo using only nanopore sequencing data. Nature Methods 2015 jul;12(8):733-735.)
The mentioned citation has a DOI (https://doi.org/10.1038/nmeth.3444). I believe it should be mentioned, instead of or in addition to the GitHub page, in order to maintain consistency with other citations of published papers/softwares.

Fixed, thank you.

Level of interest:An article of importance in its field
Quality of written English: Good
Statistical review:No, the manuscript does not need to be seen by a statistician.

Reviewer #2:
1. The authors state that the data is collected from publications, preprints, social media posts, and public and private repositories. Is this a manual effort, or is there a database crawler that's implemented to identify new tools? If it is manual, how will the authors ensure that the database is continually updated to reflect the latest tools?

Updating the database is still a manual process. In time we hope to develop a base of regular contributors from the community of users to ensure the longevity of the project. This model has so far been successful for the scRNA-tools database, created 3 years ago and now maintained by a team of volunteers around the world.

2. While the number of citations is a helpful indicator of widely-used tools, it can often be dominated by those tools that have been around longer than others and, therefore, can be misleading for those who want to use the latest and most helpful tools. One suggestion is to create a second metric that is the "number of citations within the last six months" (or one year—whichever is most practical), which can give the user an idea of tools that are gaining popularity as opposed to those that are becoming outdated. Another way to implement this idea would be to generate an interactive plot of the number of citations for each tool over time.

Thank you for the suggestions, we now report the number of citations in the past year, obtained through the citecorp R package. We have updated Figure 3 to illustrate this functionality and added a description in the Database implementation section.

3. In searching the database myself, I found a number of tools that are either misnamed or missing:

Misnamed: Isoseq4 (this should be Isoseq3)
Missing: HiFiasm, GraphAligner, MosaicFlye, TandemFlye, TandemTools, Winnowmap

Each of the missing tools are on the cutting edge of genome assembly and quality assessment and have

gained popularity in recent months. This underscores the necessity for a database crawler that is more comprehensive than manual cataloging.

We have made the corrections and additions that were raised, thank you.

4. I also found an instance where the same category is listed twice for one tool. The tool "minoTour" has the following categories listed with it: "Alignment, Alignment, Quality Checking, SNP And Variant Analysis, Visualisation, Provide Summary Statistics, Analysis Pipelines, Metagenomics". There should only be one instance of "Alignment".

Thank you for pointing this out, it has been corrected.

5. Are there any types of tools that are excluded from the database? If so, the article and website should provide a description of those that are not included.

We do not currently exclude any tools. We debated whether tools that are introduced in preprints without a link to the code should be excluded, but they are very rare cases so we include them with a warning in the description.

6. Typo in the abstract: "320" should be "32".

Fixed.

Close
