# Supplementary material for: long-read-tools.org: an interactive catalogue of analysis methods for long-read sequencing data
Source: Gigascience. 2021 Feb 16;10(2):giab003. doi: 10.1093/gigascience/giab003 (PMC7931822; doi:10.1093/gigascience/giab003)
Supplement: giab003_Response_to_Reviewer_Comments_Revision_1 [file giab003_response_to_reviewer_comments_revision_1.pdf]

Author's Response To Reviewer Comments

Close

Dear Dr Zauner,

We are delighted to hear that our manuscript is accepted for publication, in principle. We have included the reference to the GigaDB repository as requested. Please also note a minor correction to Figure 3A, where the tool SMARTdenovo had the wrong doi and therefore erroneous citation counts. We have replotted the figure and removed a mention of SMARTdenovo in the text.
The manuscript files required for production are included in the resubmission. Thank you for your competent handling of our manuscript, it has been a smooth experience.
All the best,

Quentin Gouil
Senior postdoctoral Researcher
Epigenetics and Development Division
Walter and Eliza Hall Institute

Close
