# Supplementary material for: long-read-tools.org: an interactive catalogue of analysis methods for long-read sequencing data
Source: Gigascience. 2021 Feb 16;10(2):giab003. doi: 10.1093/gigascience/giab003 (PMC7931822; doi:10.1093/gigascience/giab003)
Supplement: giab003_Response_to_Reviewer_Comments_Revision_2 [file giab003_response_to_reviewer_comments_revision_2.pdf]

Author's Response To Reviewer Comments

Close

Dear Dr Zauner,

1) I'm often mystified by the Editorial Manager's LaTeX behaviour! It looks like the pdf compiled without errors but the log is shown rather than the output pdf. I have tried with a different filename, changing the type from main LaTeX file to Manuscript, uploading the full zipped folder,... I couldn't work it out. I have been using the Gigascience template so hopefully it will be straightforward for the production team. It should just be a bug in EM and not with the LaTeX document.
2) Ref 34 now shows the doi.
Apologies for the back and forth. All the best,
Quentin

Close
