# Supplementary material for: long-read-tools.org: an interactive catalogue of analysis methods for long-read sequencing data
Source: Gigascience. 2021 Feb 16;10(2):giab003. doi: 10.1093/gigascience/giab003 (PMC7931822; doi:10.1093/gigascience/giab003)
Supplement: giab003_Reviewer_1_Report_Original_Submission — Pierre Morisse, Ph.D -- 9/11/2020 Reviewed [file giab003_reviewer_1_report_original_submission.pdf]

Reviewer Report

Title: long-read-tools.org: an interactive catalogue of analysis methods for long-read sequencing data

Version: Original Submission **Date:** 9/11/2020

Reviewer name: Pierre Morisse, Ph.D

Reviewer Comments to Author:

The authors describe long-read-tools.org, an open-source a highly complete database that gathers a large collection of long-read data analysis tools. The tools presented in the database represent a wide variety of applications, including error-correction, de novo assembly and alignement, to name a few. The database is extremely complete, providing information about more than 438 different tools as of today. Gathering and collecting information for such an amount of tools represents a massive amount of work.

I strongly believe such a database as a high potential and will be useful to the whole community.

The "Table" tab presents detailed and complete information about each tool, and filters can be applied to display additional information such as the strengths and weaknesses, the underlying algorithms, and the overall performance of the tools. Category filters can also be applied, e.g. to only display tools related to de novo assembly.

The database is very user-friendly. Developers can easily submit or modify their tools. I tried submitting a tool that was missing myself, and the process was easy and clear.

The authors also mention interesting future works for improvement, such as a "Qucik-Start" tab highlighting validated workflows, and a "Benchmark" tab, featuring benchmarking studies and their results. I believe such features would be of high-interest.

Moreover, the GitHub project's README is clear and complete, and each category is briefly explained.

I don't have any major comments about the manuscript nor the database, but list some remarks and minor comments below.

1) When on the "Tools" tab, if I click on a tool, then on one of its categories, I'm redirected to the "Tools" tab, but the chosen category is not taken into account.

The complete list of tools is thus displayed, and forces the user to re-enter the desired category.

I believe being able to access the tools related to a given category when clicking it from the description of a tool would improve user-friendliness.

2) Platforms sometimes appear to be wrong.

For instance, CONSENT, which I developed, is listed as "Python", when most of its code is written in C++. Actually, the GitHub repository contains a Python script in its "bin" folder, but GitHub does report that most of the code is written in C++.

I'm not exactly sure how the platform is inferred (By analyzing the bin folder? The language distribution indicated on GitHub? Manually?), and if other tools are affected, but if it is automatically inferred, I believe it should be verified to keep the database as precise as possible.

3) The authors state that "For instance, it is clear that all the resulting tools from the above use case can work with PacBio data while only a subset work with ONT data."

However, it does not appear to be entirely clear why the tools would work on PacBio but not on Nanopore.
How is the distinction made exactly? Is it according to the experiments performed in the paper related to the tool, e.g. if it is tested only on PacBio data?
The PacBio and Nanopore reads share a common ground (long sequences, high error-rates), and I don't exactly understand why a given tool, especially for error correction which is underlined in the example, would work on PacBio but not on ONT.
I do understand that some tools were only evaluated on PacBio and perform better on PacBio data, e.g. because of the lower error-rate of PacBio reads compared to ONT, but I don't see why they would not work at all on ONT data.
4) The authors mention that "Tools specifically focused on long-read sequence analysis became available around 2013, coinciding with the commercial release of PacBio Biosciences and Oxford Nanopore Technologies [15] sequencing platforms."
However, tools specifically designed for long-reads actually stated to emerge in 2012, see for instance error-correction tools PBcR (http://doi.org/10.1038/nbt.2280) and LSC (http://doi.org/10.1371/journal.pone.0046679).
Typos / Bibliography remarks :
1) "Moreover, the number of tools written in Python outnumber tools implemented in other programming languages (Figure 2D)."
I believe this sentence should be rewritten, e.g. simply "Moreover, tools written in Python outnumber tools implemented in other programming languages".
2) "long-read-tools.orgis both more comprehensive and easier to navigate than these databases."
A space is missing between "long-read-tools.org" and "is"
3) Citation 6 (R Development Core Team. R: A Language and Environment for Statistical Computing. Vienna, Austria: R Foundation for Statistical Computing; 2012, http://www.R-project.org, ISBN 3-900051-07-0, http://www.R-project.org.)
The link to the R project is reported two times. I believe one of the links could be omitted.
4) Citation 19 (Loman NJ, Quick J, Simpson JT. A complete bacterial genome assembled de novo using only nanopore sequencing data. Nature Methods 2015 jul;12(8):733-735.)
The mentioned citation has a DOI (https://doi.org/10.1038/nmeth.3444). I believe it should be mentioned, instead of or in addition to the GitHub page, in order to maintain consistency with other citations of published papers/softwares.
Level of interest:An article of importance in its field
Quality of written English: Good
Statistical review:No, the manuscript does not need to be seen by a statistician.

Level of Interest

Please indicate how interesting you found the manuscript: Choose an item.

Quality of Written English

Please indicate the quality of language in the manuscript: Choose an item.

Declaration of Competing Interests

Please complete a declaration of competing interests, considering the following questions:

- Have you in the past five years received reimbursements, fees, funding, or salary from an organisation that may in any way gain or lose financially from the publication of this manuscript, either now or in the future?
- Do you hold any stocks or shares in an organisation that may in any way gain or lose financially from the publication of this manuscript, either now or in the future?
- Do you hold or are you currently applying for any patents relating to the content of the manuscript?
- Have you received reimbursements, fees, funding, or salary from an organization that holds or has applied for patents relating to the content of the manuscript?
- Do you have any other financial competing interests?
- Do you have any non-financial competing interests in relation to this paper?

If you can answer no to all of the above, write 'I declare that I have no competing interests' below. If your reply is yes to any, please give details below.

I declare that I have no competing interests

I agree to the open peer review policy of the journal. I understand that my name will be included on my report to the authors and, if the manuscript is accepted for publication, my named report including any attachments I upload will be posted on the website along with the authors' responses. I agree for my report to be made available under an Open Access Creative Commons CC-BY license (http://creativecommons.org/licenses/by/4.0/). I understand that any comments which I do not wish to be included in my named report can be included as confidential comments to the editors, which will not be published.

Choose an item.

To further support our reviewers, we have joined with Publons, where you can gain additional credit to further highlight your hard work (see: https://publons.com/journal/530/gigascience). On publication of this paper, your review will be automatically added to Publons, you can then choose whether or not to claim your Publons credit. I understand this statement.

Yes Choose an item.
