# Supplementary material for: long-read-tools.org: an interactive catalogue of analysis methods for long-read sequencing data
Source: Gigascience. 2021 Feb 16;10(2):giab003. doi: 10.1093/gigascience/giab003 (PMC7931822; doi:10.1093/gigascience/giab003)
Supplement: giab003_Reviewer_2_Report_Original_Submission — Glennis Logsdon, Ph.D. -- 9/29/2020 Reviewed [file giab003_reviewer_2_report_original_submission.pdf]

Reviewer Report

Title: long-read-tools.org: an interactive catalogue of analysis methods for long-read sequencing data

Version: Original Submission **Date:** 9/29/2020

Reviewer name: Glennis Logsdon, Ph.D.

Reviewer Comments to Author:

In this manuscript, Amarasinghe et al. describe their open-source database (long-read-tools.org) that catalogs and organizes long-read data analysis tools ranging from basecalling to error-correction to assembly to isoform detection. These tools are not limited to traditional long-read sequencing data types (i.e., Pacific Biosciences and Oxford Nanopore Technologies) but also include synthetic long-read sequencing data types such as Hi-C, 10X, and Bionano Genomics. Their online database is easy to use, versatile, and more comprehensive than existing long-read tool databases, and it has the potential to be quite useful for novices who are starting to work with long-read sequencing data as well as more experienced users who are switching from one data type to another. The utility of the website will increase with the addition of tutorials and recommendations that the authors are currently planning to implement. These new additions will be important to help users navigate the 400+ tools that are currently catalogued. I recommend some minor revisions, as detailed below.

Minor criticisms

1. The authors state that the data is collected from publications, preprints, social media posts, and public and private repositories. Is this a manual effort, or is there a database crawler that's implemented to identify new tools? If it is manual, how will the authors ensure that the database is continually updated to reflect the latest tools?
2. While the number of citations is a helpful indicator of widely-used tools, it can often be dominated by those tools that have been around longer than others and, therefore, can be misleading for those who want to use the latest and most helpful tools. One suggestion is to create a second metric that is the "number of citations within the last six months" (or one yearâ€"whichever is most practical), which can give the user an idea of tools that are gaining popularity as opposed to those that are becoming outdated. Another way to implement this idea would be to generate an interactive plot of the number of citations for each tool over time.
3. In searching the database myself, I found a number of tools that are either misnamed or missing:
Misnamed: Isoseq4 (this should be Isoseq3)
Missing: HiFiasm, GraphAligner, MosaicFlye, TandemFlye, TandemTools, Winnowmap
Each of the missing tools are on the cutting edge of genome assembly and quality assessment and have gained popularity in recent months. This underscores the necessity for a database crawler that is more comprehensive than manual cataloging.
4. I also found an instance where the same category is listed twice for one tool. The tool "minoTour" has the following categories listed with it: "Alignment, Alignment, Quality Checking, SNP And Variant Analysis, Visualisation, Provide Summary Statistics, Analysis Pipelines, Metagenomics". There should only be one instance of "Alignment".

5. Are there any types of tools that are excluded from the database? If so, the article and website should provide a description of those that are not included.
6. Typo in the abstract: "320" should be "32".

Level of Interest

Please indicate how interesting you found the manuscript: Choose an item.

Quality of Written English

Please indicate the quality of language in the manuscript: Choose an item.

Declaration of Competing Interests

Please complete a declaration of competing interests, considering the following questions:

- Have you in the past five years received reimbursements, fees, funding, or salary from an organisation that may in any way gain or lose financially from the publication of this manuscript, either now or in the future?
- Do you hold any stocks or shares in an organisation that may in any way gain or lose financially from the publication of this manuscript, either now or in the future?
- Do you hold or are you currently applying for any patents relating to the content of the manuscript?
- Have you received reimbursements, fees, funding, or salary from an organization that holds or has applied for patents relating to the content of the manuscript?
- Do you have any other financial competing interests?
- Do you have any non-financial competing interests in relation to this paper?

If you can answer no to all of the above, write 'I declare that I have no competing interests' below. If your reply is yes to any, please give details below.

I declare that I have no competing interests.

I agree to the open peer review policy of the journal. I understand that my name will be included on my report to the authors and, if the manuscript is accepted for publication, my named report including any attachments I upload will be posted on the website along with the authors' responses. I agree for my report to be made available under an Open Access Creative Commons CC-BY license (http://creativecommons.org/licenses/by/4.0/). I understand that any comments which I do not wish to be included in my named report can be included as confidential comments to the editors, which will not be published.

Choose an item.

To further support our reviewers, we have joined with Publons, where you can gain additional credit to further highlight your hard work (see: https://publons.com/journal/530/gigascience). On publication of this paper, your review will be automatically added to Publons, you can then choose whether or not to claim your Publons credit. I understand this statement.

Yes Choose an item.
